# Mechanistic Promiscuity in Cobalt‐Mediated CO_2_ Reduction Reaction: One‐ Versus Two‐Electron Reduction Process

**DOI:** 10.1002/anie.202503705

**Published:** 2025-06-30

**Authors:** Ayan Bera, Sarah Bimmermann, Philipp Gerschel, Dibya Jyoti Barman, Leon Gerndt, Thomas Lohmiller, Kaltum Abdiaziz, Alexander Schnegg, Maylis Orio, Dennis G. H. Hetterscheid, Kara L. Bren, Michael Roemelt, Ulf‐Peter Apfel, Kallol Ray

**Affiliations:** ^1^ Institut für Chemie, Humboldt‐Universität zu Berlin Brook‐Taylor‐Str. 2 12489 Berlin Germany; ^2^ Faculty of Chemistry & Biochemistry, Ruhr‐Universität Bochum, Universitätsstraße 150 44801 Bochum Germany; ^3^ EPR4Energy Joint Lab, Department Spins in Energy Conversion and Quantum Information Science Helmholtz Zentrum Berlin für Materialien und Energie GmbH Albert‐Einstein‐Straße 16 12489 Berlin Germany; ^4^ Max Planck Institute for Chemical Energy Conversion 34‐36 Stiftstraße 45470 Mülheim an der Ruhr Germany; ^5^ Aix‐Marseille Univ, CNRS, Centrale Marseille iSm2 Marseille France; ^6^ Leiden Institute of Chemistry Leiden University P.O. Box 9502 Leiden 2300 RA The Netherlands; ^7^ Department of Chemistry University of Rochester Rochester New York 14627‐0216 USA; ^8^ Department of Electrosynthesis Fraunhofer UMSICHT Osterfelder Straße 3 46047 Oberhausen Germany

**Keywords:** CO_2_ reduction, Electrocatalysis, H‐bonding, Oxalate, Redox non‐innocent ligands

## Abstract

We compare the carbon dioxide reduction (CO_2_RR) activity and selectivity of the complexes [**(Hbbpya)Co^II^**]**^2+^** and [**(Mebbpya)Co^II^**]**^2+^**, which contain two 2,2′‐bipyridine chelating groups linked by ‐NH or ‐NCH_3_ moieties, respectively. Whereas [**(Hbbpya)Co^II^**]**^2+^** forms CO under electrocatalytic conditions in presence of phenol (PhOH) with high selectivity, [**(Mebbpya)Co^II^**]**^2+^** shows higher hydrogen evolution reaction activity and low selectivity for CO production. The molecular origin of the difference in product selectivity was analysed based on spectroscopic trapping of reactive intermediates and detailed kinetic and theoretical studies. A difference in mechanism is evident; whereas an efficient proton relay mediated by the ‐NH group initiates a two‐electron reduction of CO_2_ in the case of [**(Hbbpya)Co^II^**]**^2+^**, one‐electron chemistry prevails for [**(Mebbpya)Co^II^**]**^2+^**. Under stopped‐flow conditions, we trapped the one‐electron reduced CO_2_ radical anion in [**(Mebbpya)Co^I^(CO_2_^−•^)**], which forms oxalate under aprotic conditions. This study underlines the importance of subtle electronic and protonation changes in controlling the CO_2_RR product selectivity.

## Graphical abstract

The cobalt complexes [**(Hbbpya)Co^II^**]^2^⁺, and [**(Mebbpya)Co^II^**]^2^⁺ show contrasting CO₂ reduction behaviors. [**(Hbbpya)Co^II^**]^2^⁺ selectively forms CO via a fast, two‐electron pathway aided by a ‐NH proton relay, while **[(Mebbpya)Co^II^]^2^⁺** favours hydrogen evolution due to a slower, competitive one‐electron mechanism. Stopped‐flow studies reveal a reactive CO₂ radical anion, offering insights into their distinct catalytic pathways.

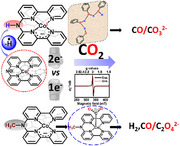

## Introduction

The transformation of CO_2_ into valuable carbon‐based fuels through reduction presents a sustainable approach to addressing global energy demands and reducing greenhouse gas emissions.^[^ ^1^, ^2^, ^3^, ^4^ ^]^ Efficient catalysts are necessary that can selectively perform CO_2_ reduction reactions (CO_2_RR) to various C_1_ and C_2_ products over the kinetically and thermodynamically competitive hydrogen evolution reaction (HER).^[^ ^5^ ^]^ Despite advances in the development of molecular catalysts with highly specialized ligand scaffolds, including cyclams,^[^ ^6^, ^7^, ^8^, ^9^, ^10^, ^11^, ^12^, ^13^, ^14^ ^]^ porphyrins,^[^ ^15^, ^16^, ^17^, ^18^, ^19^, ^20^, ^21^, ^22^ ^]^ phthalocyanines,^[^ ^23^, ^24^ ^]^ corroles,^[^ ^25^, ^26^ ^]^ and polypyridines,^[^ ^27^, ^28^, ^29^, ^30^, ^31^, ^32^, ^33^ ^]^ spectroscopic trapping of the key reactive intermediates are lacking in many cases,^[^ ^34^, ^35^, ^36^ ^]^ which makes the mechanism ambiguous.

**Scheme 1:**
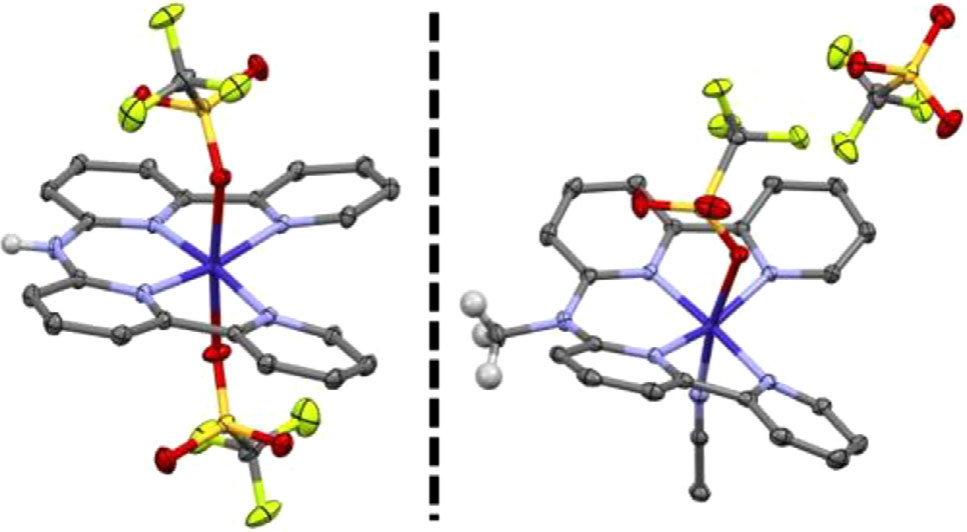
Molecular structures of [**(Hbbpya)Co^II^**]**^2+^** (left), and [**(Mebbpya)Co^II^**]**^2+^** (right) as obtained from single crystal X‐ray diffraction (XRD) measurements. Thermal ellipsoids are drawn at the 50% probability level. All hydrogen atoms except for N‐H and N‐CH_3_ have been omitted for clarity.

Herein, we compare the electrocatalytic CO_2_RR activities and selectivities of two molecular catalysts, [**(Hbbpya)Co^II^**]^2+^ and [**(Mebbpya)Co^II^**]^2+^ (Scheme 1) that contain a Co(II)‐center that is chelated by two redox‐active 2,2′‐bipyridine (bpy) groups linked by ‐NH or ‐N(CH_3_) groups, respectively. Interestingly, although both [**(Hbbpya)Co^II^**]^2+^ and [**(Mebbpya)Co^II^**]^2+^ can perform CO_2_RR with moderate overpotential (500 mV), only [**(Hbbpya)Co^II^**]^2+^ exhibits a high selectivity for CO production (92%) in acetonitrile (MeCN), employing PhOH as a proton donor. The difference in selectivity is attributed to the requirement for both an efficient proton shuttle, where the N‐H group acts as an anchor point for a phenol network, and also a redox non‐innocent ligand for selective CO_2_‐to‐CO conversion. While ligand non‐innocence^[^ ^37^, ^38^, ^39^, ^40^, ^41^ ^]^ and proton shuttles^[^ ^27^, ^42^, ^43^, ^44^, ^45^, ^46^, ^47^, ^48^, ^49^, ^50^, ^51^ ^]^ involving secondary coordination spheres^[^ ^52^ ^]^ and water networks^[^ ^43^, ^45^ ^]^ are known to separately impact the catalytic efficiency of both HER and CO_2_RR, we show in this study the concurrent effect of both of these factors in controlling the two‐ versus one‐electron reduction of CO_2_. Most importantly, in the case of [**(Mebbpya)Co^II^**]^2+^, we have trapped a transient intermediate arising from the rare one‐electron reduction of CO_2_ under stopped‐flow conditions, which under aprotic conditions enables a symmetric coupling of two CO_2_ molecules to form the oxalate dianion. Notably, conversion of CO_2_ to the oxalate anion, which has been controversially^[^ ^53^ ^]^ discussed in the literature, provides an ideal model reaction to investigate the energy‐relevant C─C bond formation reactions in chemistry and biology.

## Results and Discussion

### Synthesis and Characterization of the Co(II) Complexes

The ligands *N*,*N*‐bis(2,2′‐bipyrid‐6‐yl)amine (Hbbpya), and N‐([2,2′‐bipyridin]‐6‐yl)‐N‐methyl‐[2,2′‐bipyridin]‐6‐amine (Mebbpya) and the corresponding cobalt complexes have been synthesized as previously described^[^ ^54^ ^]^ and characterized thoroughly (Schemes S1–S3, Figures S1–S10). The solid‐state structures of [**(Hbbpya)Co^II^**]^2+^ and [**(Mebbpya)Co^II^**]^2+^ exhibit a distorted octahedral geometry with four N atoms from the bipyridine moieties coordinated at the equatorial positions, and either the triflate counteranions or one triflate and one solvent molecule are bound in the two axial positions (Scheme 1). Both of the complexes show similar structural properties; the difference in Co‐N bond lengths between the structures is below 1 pm (Table S7), and the N‐N‐N‐N dihedral angles amount to 9.7 and 11.2 degrees for [**(Hbbpya)Co^II^**]^2+^ and [**(Mebbpya)Co^II^**]^2+^, respectively (Figure S11). Furthermore, both complexes are stabilized in their *S *= 1/2 ground state, as evident from their X‐band EPR spectra (Figure S12). DFT calculations reveal that the unpaired electron in [**(Hbbpya)Co^II^**]^2+^ and [**(Mebbpya)Co^II^**]^2+^ is located in a d_z_^2^‐type orbital of the Co center (Figure S13 for [**(Hbbpya)Co^II^**]^2+^) and the computed *g* values reproduce the experimentally observed values well (Table S8).

### Synthesis and Characterization of the Reduced Complexes

**Figure 1:**
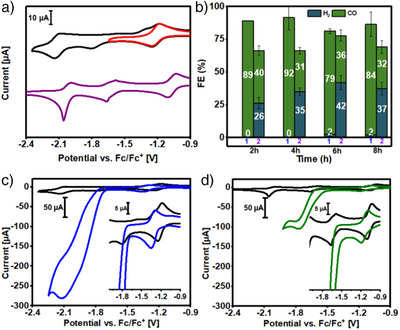
a) CV data of 0.5 mM solutions of [**(Hbbpya)Co^II^**]^2+^ (black trace), and [**(Mebbpya)Co^II^**]^2+^ (purple trace) under Ar in dry MeCN with 0.1 M TBAPF_6_ at a scan rate of 100 mV s^−1^. The red trace shows the CV of [**(Hbbpya)Co^II^**]^2+^ when scanned up to −1.65 V. b) Results of the product analysis of the long‐term electrolysis of CO_2_ saturated MeCN solutions of [**(Hbbpya)Co^II^**]^2+^ (1), and [**(Mebbpya)Co^II^**]^2+^ (2) at −1.80 V, and −1.75 V respectively, in presence of 3 M PhOH. CVs of 0.5 mM (c) [**(Hbbpya)Co^II^**]^2+^, and (d) [**(Mebbpya)Co^II^**]^2+^ under Ar (black) and CO_2_ with 3 M PhOH (blue trace, [**(Hbbpya)Co^II^**]^2+^; green trace [**(Mebbpya)Co^II^**]^2+^; scan rate 100 mV s^−1^). The insets show expansions of −0.9  to −1.8 V.

**Figure 2:**
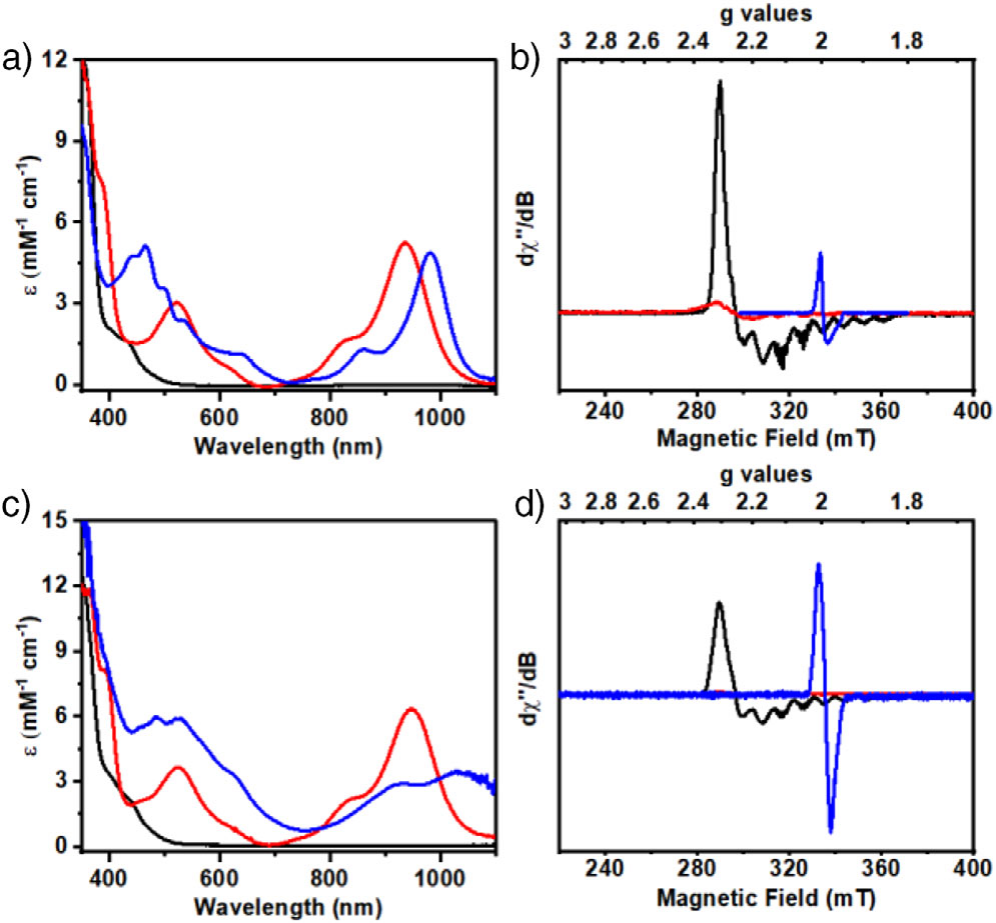
Comparison of the a) UV‐Vis and b) X‐band EPR (at 13 K) spectra of 0.25 mM [**(Hbbpya)Co^II^**]^2+^ (black) with chemically generated [**(Hbbpya)Co^I^**]^+^ (red), and mixture (blue; see text) of [**(Hbbpya^• –^)Co^I^**]^0^ (16%) and [**(bbpya^−^)Co^I^**]^0^ (84%), in butyronitrile at 25 °C. The corresponding c) UV‐Vis and d) EPR spectra of the [**(Mebbpya)Co^II^**]^2+^ (black), [**(Mebbpya)Co^I^**]^+^ (red) [λ_max_ (ε, M^−1^ cm^−1^) = 532 (3600), 826 (2000), and 945 (6200) nm], and [**(Mebbpya^.−^)Co^I^**]^0^ (blue) [λ_max_ (ε, M^−1^ cm^−1^) = 483 (6000), 921 (2800), and 1035 (3300) nm] series are also shown for comparison purpose.

The black and red traces in Figure 1a represent cyclic voltammograms of [**(Hbbpya)Co^II^**]^2+^ in MeCN solution containing 0.1 M [N(n‐Bu)_4_]PF_6_ as the supporting electrolyte. Complex [**(Hbbpya)Co^II^**]^2+^ features a reversible (Figure S14A–B) cobalt‐based one‐electron reduction at *E*_1/2_ = −1.21 V and irreversible (see Figure S14C–F) ligand‐based reduction processes at *E*_red_ = −1.78  and −2.11 V (all potentials are referenced vs. Fc/Fc^+^). The assignment of the redox processes (Co vs. ligand based) is made by comparison with the CV of [**(Hbbpya)Zn^II^**]^2+^ (Figure S15), which has been also extensively characterized (Figures S16–20). The UV‐Vis spectrum of *S* = 1/2 [**(Hbbpya)Co^II^**]^2+^ in MeCN is featureless in the visible region (Figure 2a, black trace). Chemical or electrochemical one‐electron reductions of [**(Hbbpya)Co^II^**]^2+^ lead to EPR‐silent [**(Hbbpya)Co^I^**]^+^, with characteristic UV‐Vis absorptions at λ_max_ (ε, M^−1^ cm^−1^): 527 (3000), 827 (1600), and 937 (5200) (Figure 2a,b, red trace; Figure S21).

The NIR features presumably originate from a Co(I)‐to‐ligand charge transfer transition in [**(Hbbpya)Co^I^**]^+^, which corroborate the Co(II)‐ligand radical state being close in energy relative to the ground low‐spin *S* = 0 Co(I) configuration (Table S5). Indeed, initial studies with multireference (MR) electronic structure methods indicate that the electronic ground state of [**(Hbbpya)Co^I^**]^+^ is multiconfigurational in nature, which has previously been observed for other cobalt complexes with redox non‐innocent ligands.^[^ ^55^, ^56^ ^]^

The 527‐nm band is blue‐shifted to 465 nm (5100 M^−1^ cm^−1^) and the NIR bands are red‐shifted to 860 (1200 M^−1^ cm^−1^) and 968 nm (5000 M^−1^ cm^−1^) upon further reduction of [**(Hbbpya)Co^I^**]^+^ (Figure 2a, blue trace; see also Figures S22, S23 for electrochemical generation). In EPR, it reveals a diagnostic ligand radical signal corresponding to the formation of [**(Hbbpya^• –^)Co^I^**]^0^ (**Hbbpya^• –^** represents the one‐electron reduced ligand; Figure 2b, blue trace; Figure S24A) in 10% yield (based on spin quantification; Figure S25). DFT calculation predicts a low‐spin Co(I) ground state that features a single unpaired electron delocalized over the entire ligand (Figure S26); the experimental EPR parameters could be nicely reproduced in the calculation (Table S8). However, [**(Hbbpya^• –^)Co^I^**]^0^ is metastable, and a majority of it decays to an EPR‐silent species, which is characterized by XRD to be [**(bbpya ^–^)Co^I^**]^0^ that contains a deprotonated Hbbpya ligand (Figure S27), and is formed by the release of 0.5 equivalents of hydrogen (Scheme S4; see Figure S28 for the GC‐MS detection of H_2_).

The redox chemistry of [**(Mebbpya)Co^II^**]^2+^ is similar to [**(Hbbpya)Co^II^**]^2+^ (see Figure 1a, purple trace and Figure S29 for CV data). The electronic structures of [**(Mebbpya)Co^II^**]^2+^ and the (electro)chemically generated one‐ and two‐electron reduced species are found to be analogous to the [**(Hbbpya)Co**]**^2+/+/0^** complexes, as evident from UV‐Vis and EPR studies (Figure 2c,d), and also supported by theoretical calculations (Tables S9, S10). Crystals suitable for single‐crystal XRD were obtained for the one‐electron reduced species [**(Mebbpya)Co^I^**]^+^ (Figure S30). Interestingly, the average Co‐N_bpy_ distance in [**(Mebbpya)Co^I^**]^+^ is 1.887(7) Å, which is significantly shorter than that in [**(Mebbpya)Co^II^**]^2+^ (1.946(1) Å), consistent with a stronger Co(I)‐ to‐ligand back‐bonding interaction in [**(Mebbpya)Co^I^**]^+^ (Table S4). Furthermore, the two‐electron reduced species [**(Mebbpya^• –^)Co^I^**]^0^ is more stable compared to [**(Hbbpya^• –^)Co^I^**]**^0^**, and could be generated in near‐quantitative yields (Figures 2D and S31 for spin quantification).

### Electrocatalytic Reduction of CO_2_ Catalyzed by [(Hbbpya)Co^II^]^2+^ and [(Mebbpya)Co^II^]^2+^ in MeCN

In spite of their similar electronic and geometric structures, [**(Hbbpya)Co^II^**]^2+^ and [**(Mebbpya)Co^II^**]^2+^ exhibit different reactivities and product selectivity in electrocatalytic CO_2_ reduction reactions. The different CO_2_RR activities are reflected in the CVs of [**(Hbbpya)Co^II^**]^2+^ and [**(Mebbpya)Co^II^**]^2+^ in presence of both CO_2_ and PhOH (Figure 1c,d). For [**(Hbbpya)Co^II^**]^2+^, at a scan rate of 100 mV s^−1^ (Figure 1c), the emergence of significant catalytic current with an onset potential of about −1.65 V is observed, which reaches a maximum at −2.1 V; this, together with the non‐reactivity of the chemically generated [**(Hbbpya)Co^I^**]^+^ toward CO_2_ and PhOH (as confirmed by UV‐Vis experiment; data not shown), indicates that a second one‐electron reduction is necessary to generate the reactive intermediate responsible for CO_2_RR activity. The current increases approximately linearly with increasing phenol concentration and reaches a plateau at 2.5 M (100 mV s^−1^ scan rate; Figure S32) or 1.0 M phenol (3 mV s^−1^ scan rate; Figure S33). This increase in current can be attributed to efficient CO_2_ reduction activity of [**(Hbbpya)Co^II^**]^2+^ in the presence of an acid source (3.0 M PhOH) with i_cat_/i_p_ ≈ 28 (100 mV s^−1^) or ≈ 75 (3 mV s^−1^). Notably, for [**(Mebbpya)Co^II^**]^2+^, the magnitude of the catalytic current under similar conditions (Figure 1D) is significantly smaller (i_cat_/i_p_ ≈ 10; scan rate of 100 mV s^−1^ or i_cat_/i_p_ ≈ 35; scan rate of 5 mV s^−1^; Figures S34, S35).

To verify our findings regarding the trends of CO_2_RR activity of [**(Hbbpya)Co^II^**]^2+^ and [**(Mebbpya)Co^II^**]^2+^ from CV experiments, controlled potential coulometry (CPC) was conducted over 8 h using a 0.5 mM complex solution in MeCN with 3 M PhOH as a proton source at a constant CO_2_ pressure (1 atm). Post catalysis analysis confirmed that there was no electrodeposition after 8 h and the solution concentration of the catalysts did not alter significantly (see SI for details), thereby corroborating the molecular origin of catalysis. The gaseous products were analysed in 2‐h intervals for the entire 8‐h period by injection into a gas chromatograph with a barrier discharge ionization detector (GC‐BID). At a potential of −1.8 V, [**(Hbbpya)Co^II^**]^2+^ shows a faradaic efficiency (FE) of 92(±8) % for CO after 4 h of electrolysis (Figure 1b). The whole 8 h of catalysis produced only negligible amounts of H_2_ (FE of < 2%), while the FE for the production of CO remained stable between 80% and 90%. The replacement of the −NH group in [**(Hbbpya)Co^II^**]^2+^ by ‐NCH_3_ in [**(Mebbpya)Co^II^**]^2+^, however, led to a significant decrease in selectivity for CO production and also in overall activity (CO + H_2_ production all together). After 2 h of electrocatalysis by [**(Mebbpya)Co^II^**]^2+^ at −1.75 V, FEs of 40% and 26% are measured for CO and H_2_, respectively. The extent of HER increases with time and H_2_ becomes the predominant product (over 60% selectivity for H_2_ production) between 4 and 8 h of electrocatalysis. For the two catalysts, a product analysis via GC‐MS was carried out after 8 h electrolysis to detect any formic acid formed. Only a small amount of formic acid was detected (3% for [**(Hbbpya)Co^II^**]^2+^ and 0.8% for [**(Mebbpya)Co^II^**]^2+^). Thus, the remaining charge most likely flows into decomposition processes such as solvent decomposition, which is visible by deposition of small amounts of material on the electrode. To further analyse the electrocatalytic stability of the complexes, the electrolyte solutions were analysed by UV‐Vis spectroscopy after electrolysis (see SI; page S39). The measurements show that the complexes are largely retained, so that electrochemical deposition processes are not significant.

### Kinetic Analysis of Electrochemical CO_2_RR Catalyzed by [(Hbbpya)Co^II^]^2+^ and [(Mebbpya)Co^II^]^2+^

Encouraged by the high selectivity of [**(Hbbpya)Co^II^**]^2+^ for electrochemical CO_2_RR and the difference in selectivity compared to [**(Mebbpya)Co^II^**]^2+^ towards competing HER, we sought to evaluate the kinetic factors leading to the different selectivities in the two complexes. Accordingly, CV studies were conducted using increasing PhOH and PhOD concentrations (0.5 M–3/5 M) (Figures S32–S35). Kinetic isotope effect (KIE) values were determined from the linear plots of (i_cat_/i_p_) with respect to PhOH or PhOD concentrations. A KIE value of 1.11 was calculated for [**(Hbbpya)Co^II^**]^2+^ (Figures S32, S33), which is consistent with the involvement of a rate‐determining hydrogen‐bond mediated proton transfer step from PhOH in the catalytic process to yield CO.^[^ ^57^, ^58^ ^]^ Interestingly, a significantly higher KIE of 8.50 was determined for [**(Mebbpya)Co^II^**]^2+^ (Figures S34,S35), which suggests the involvement of a Co‐H intermediate^[^ ^59^, ^60^, ^61^, ^62^, ^63^ ^]^ leading to competing HER, consistent with the CPC results. The KIE values were found to be independent of the scan rates employed. Furthermore, the plot of catalytic current density versus [**(Hbbpya)Co^II^**]^2+^ concentration shows a linear correlation (Figure S36), indicating a predominantly mononuclear mechanism^[^ ^64^, ^65^ ^]^ that is first‐order in [**(Hbbpya)Co^II^**]^2+^.

### Stoichiometric Reactions of [(Hbbpya^.−^)Co^I^]^0^ and [(Mebbpya^.−^)Co^I^]^0^ with H^+^ and CO_2_

The conclusions derived from the kinetic analysis of electrocatalytic CO_2_RR are also supported by the kinetics of the stochiometric reactions of CO_2_ and PhOH with chemically generated doubly reduced [**(Hbbpya^• –^)Co^I^**]^0^ and [**(Mebbpya^• –^)Co^I^**]° complexes. Notably, a solution containing [**(Hbbpya^• –^)Co^I^**]^0^ (16%) and its decay product [**(bbpya ^–^)Co^I^**]^0^ react with CO_2_ at room temperature as evident from the decay of the NIR band at 968 nm (Figure S37) along with the concomitant formation of CO in 61% yield (based on Co; Figure S38). The EPR spectrum of the resultant solution reveals a *S* = 1/2 signal (Figures S37B, S39) corresponding to the formation of [**(Hbbpya)Co^II^(CO_3_^2−^)**] (see Figure S40 for the molecular structure determined by SC‐XRD) in 15% yield, which matches the starting concentration of [**(Hbbpya^• –^)Co^I^**]^0^. The rest of the cobalt species is EPR silent, and presumably corresponds to non‐isolated [**(bbpya^−^)Co^III^(CO_3_^2−^)**] generated by two‐electron oxidation of [**(bbpya ^–^)Co^I^**]^0^ in the presence of CO_2_ (Scheme S5). Under stopped‐flow conditions, a pseudo‐first order rate constant of 18.51 s^−1^ was determined (Figure S41) in CO_2_‐saturated MeCN (0.28 M CO_2_).^[^ ^66^ ^]^ In the presence of PhOH, the NIR band at 968 nm undergoes a blue shift to 936 nm and decays at a rate constant (0.022 s^−1^; Figure S42) that is three orders of magnitude slower than with CO_2_. In the presence of both PhOH and CO_2_ (data not shown), the reaction becomes too fast to be followed even under stopped‐flow conditions; exclusive formation of CO is observed in near quantitative yield.

**Figure 3:**
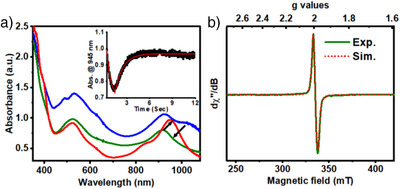
a) Changes in UV‐Vis spectra during the reaction of [**(Mebbpya^• –^)Co^I^**]^0^ (blue trace) with 0.28 M CO_2_ in MeCN to form the one electron reduced [**(Mebbpya)Co^I^**]^+^ (red trace) species via a transient intermediate [**(Mebbpya)Co^I^‐CO_2_^•−^**] (green trace) under stopped‐flow conditions. (Inset) Time trace of the band at 945 nm, which shows an initial decrease due to generation of [**(Mebbpya)Co^I^‐CO_2_^•−^**] with a rate constant of 2.31 s^−1^ and the eventual formation of [**(Mebbpya)Co^I^**]**^+^** with a rate‐constant of 0.95 s^−1^. Black square and red trace represent experimental data and first order fits, respectively. b) Experimental (green) and simulated (red dashed) EPR spectra of [**(Mebbpya)Co^I^‐CO_2_^•−^**] obtained at 0.2 s after addition of CO_2_ in butyronitrile at 14 K. The simulated spectra employed the following parameters: *g*_x_ = 2.008, *g*_y_ = 1.994, *g*_z_ = 1.972.

For [**(Mebbpya^• –^)Co^I^**]^0^, the reactivity trend is reversed; a faster reaction with PhOH (7.76 s^−1^) relative to CO_2_ (2.31 s^−1^) is observed, leading to the stoichiometric formation of H_2_ (Figures S43, S45). Furthermore, in contrast to [**(Hbbpya^• –^)Co^I^**]^0^ or [**(bbpya ^–^)Co^I^**]^0^, which act as two‐ electron reductants for CO_2_, [**(Mebbpya^• –^)Co^I^**]° can only transfer one electron to CO_2_, forming the one‐electron oxidized [**(Mebbpya)Co^I^**]^+^ species, as evident from the generation of the UV‐Vis band at 945 nm (Figure 3a, red trace) corresponding to [**(Mebbpya)Co^I^**]^+^. The conversion of [**(Mebbpya^• –^)Co^I^**]^0^ to [**(Mebbpya)Co^I^**]^+^ in the presence of CO_2_, however, involves the generation of an intermediate, as evident from stopped‐flow studies (Figure 3a; green trace). We tentatively assign the intermediate to [**(Mebbpya)Co^I^‐CO_2_^• –^**] species, which shows UV‐Vis absorption features at λ_max_ (ε, M^−1^ cm^−1^): 522 nm (2120) and 918 nm (1400), and a typical radical‐like EPR feature with [*g_x_*, *g_y_*, *g_z_*] = [2.008, 1.994, 1.972] (Figure 3b). When ¹^3^CO₂ is used in the reaction, additional broadening is observed in the EPR spectrum, which could be simulated by incorporating weak anisotropic hyperfine coupling from the ¹^3^C nucleus with [*A*x, *A*y, *A*z] = [0, 21, 69] MHz (Figure S46). Under aprotic conditions, [**(Mebbpya)Co^I^‐CO_2_^• –^**] undergoes a bimolecular decay to yield oxalate and [**(Mebbpya)Co^I^**]^+^. The formation of oxalate anion was confirmed on the basis of ^13^C NMR and IR spectroscopy and by comparison with authentic H_2_C_2_O_4_, K_2_C_2_O_4_, and K_2_CO_3_ (Figures S47–S51) samples. The origin of the oxalate was confirmed to be CO₂, as evidenced by the shift in the C‐O stretching vibration upon ^13^CO_2_ labelling, which corresponds to the formation of ¹^3^C‐labeled oxalate (Figure S51).

### Proposed Mechanism Supported by DFT Calculations

Two mechanisms consistent with the experimental observations include intramolecular phenol‐assisted proton transfer from the pendant amine to CO_2_ or intermolecular pendant amine‐assisted proton transfer from phenol to CO_2_. However, based on the geometric and energetic considerations, we exclude the intramolecular mechanism. It would warrant a direct proton transfer from the equatorial amine to the axially coordinated CO_2_ ligand, which according to our geometrical analysis is expected to be strongly hindered. The relevant N─H bond of the equatorial amine points away from the CO_2_ ligand, as evidenced by the H_eq. amine_–N_eq. amine_–Co angle of 178.8 degrees and the relatively large distance between this hydrogen and the nearest CO_2_ oxygen (approx. 4.14 Å, see Figure S52a). Moreover, we evaluated the transformation of **[(Hbbpya)CoCO_2_]^0^** to **[(bbpya)CoCO_2_H]^0^** energetically. According to our results at the DFT level of theory, the intramolecular proton transfer is endergonic by approximately +10.7 kcal mol^−1^, further suggesting that it is unfavourable.

**Scheme 2:**
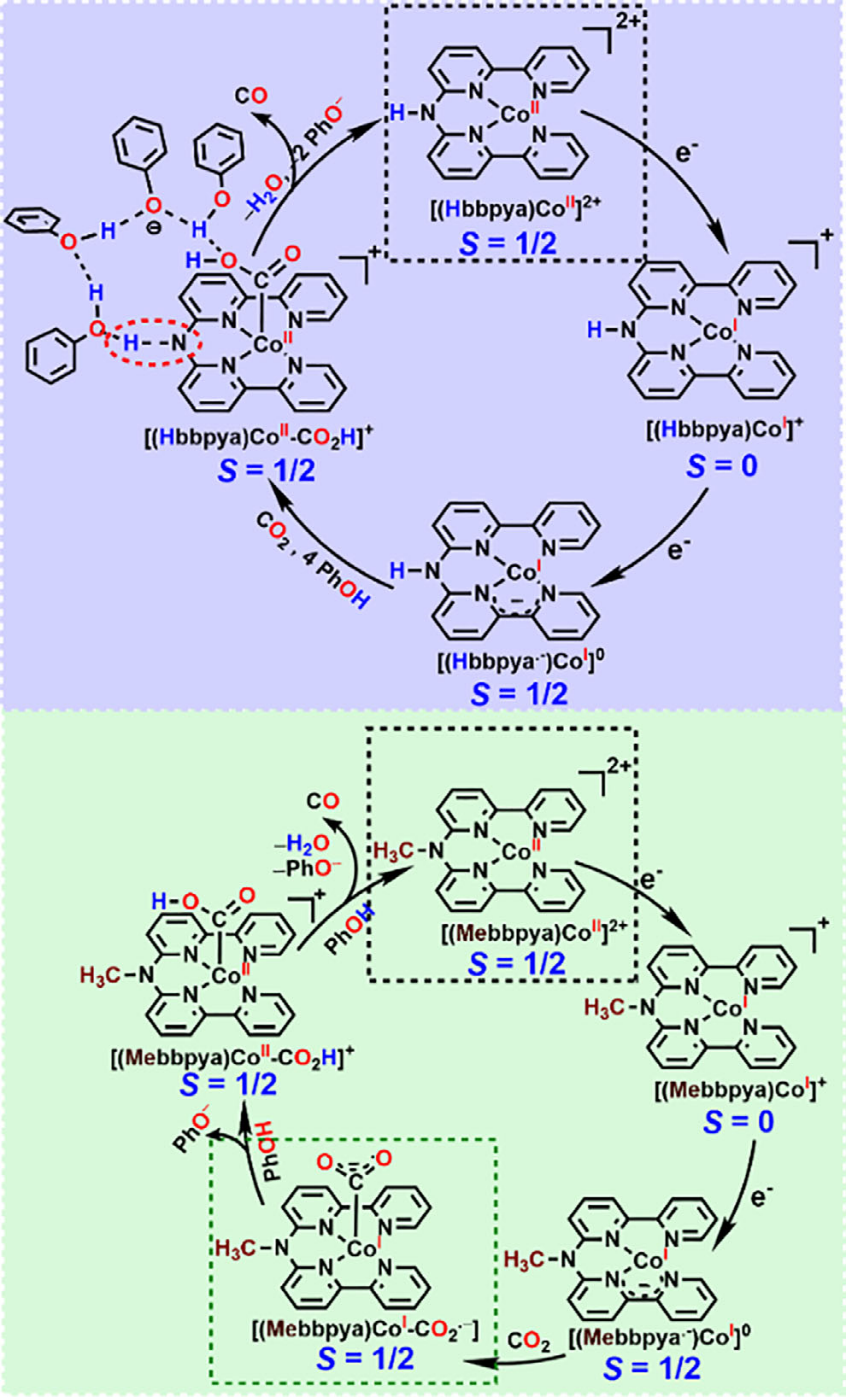
Proposed mechanism for electrocatalytic CO_2_ reduction by [**(Hbbpya)Co^II^**]^2+^ (top) and [**(Mebbpya)Co^II^**]^2+^ (bottom).

**Figure 4:**
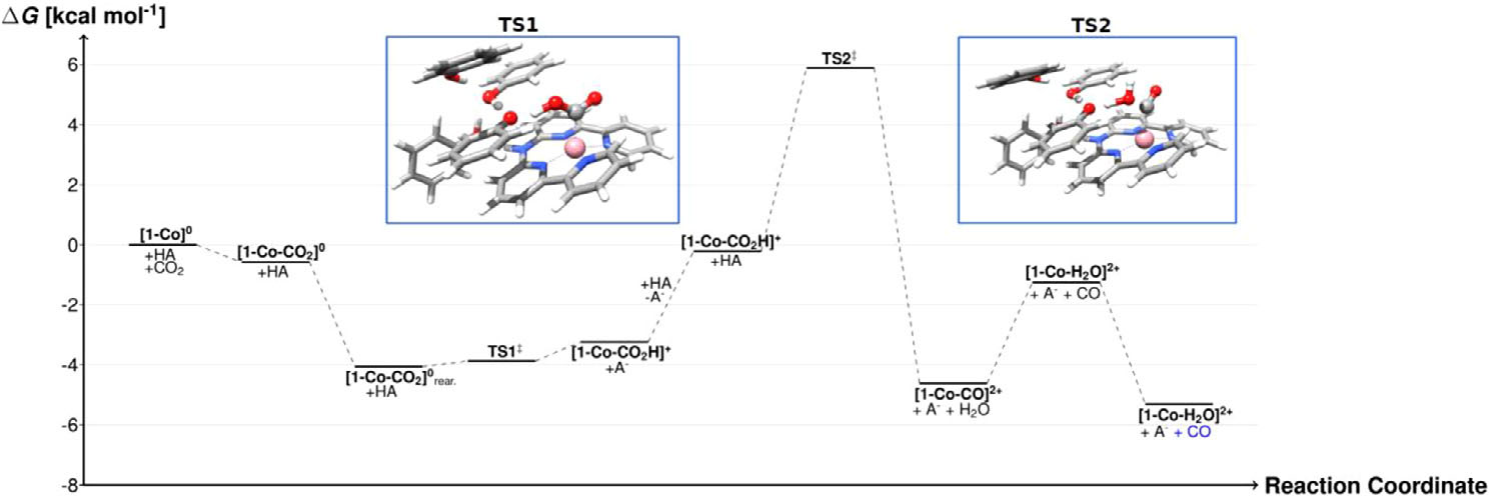
Free energy profile of CO_2_ reduction by [**(Hbbpya^•−^)Co^I^**]**^0^ [1‐ Co]^0^** under catalytic conditions as obtained from DFT calculations (Tables S16–S19). TS1 and TS2 correspond to transition states on the electronic potential energy surface (PES) involving a network (HA) containing 4 PhOH molecules. For the structures of **[1‐Co‐CO_2_**]^o^ and **[1‐Co‐CO_2_**]^o^_rear._ see Figure S52b. Due to subtle differences in the thermochemical corrections (translational, rotational, vibrational entropy, and enthalpy), the energy of TS1 is by ∼ 1 kcal mol^−1^ lower than that of [**(Hbbpya)Co^II^‐CO_2_H**]**^+^** (**[1‐Co‐CO_2_H]^+^)**. The CO highlighted in blue indicates that the CO molecule has been computed separately leading to a considerable gain in translational entropy. A^−^ is a cluster of 3 PhOH with one phenolate.

Our calculations rather support an intermolecular proton‐transfer mechanism as shown in Scheme 2 and Figure 4. The starting [**(Hbbpya)Co^II^**]^2+^ must initially undergo a two‐electron reduction process to form [**(Hbbpya^• –^)Co^I^**]^0^ or [**(bbpya ^–^)Co^I^**]^0^, both of which are capable of performing two‐electron reduction of CO_2_. Under catalytic conditions with excess PhOH and CO_2_ the formation of [**(bbpya ^–^)Co^I^**]^0^ is likely suppressed and the sole reactive intermediate is [**(Hbbpya^• –^)Co^I^**]^0^. DFT calculations suggest that the ‐NH group in [**(Hbbpya^• –^)Co^I^**]^0^ interacts via a hydrogen bond to a PhOH moiety and thereby acts as a structural anchor to organize further PhOH molecules that form a hydrogen‐bonded network (Scheme 2, top), which facilitates the binding of CO_2_ to the Co center. When four PhOH molecules are assembled in the hydrogen‐bonded network and a CO_2_ molecule approaches the Co center, formation of [**(Hbbpya)Co^II^‐CO_2_^2−^**], where CO_2_ is doubly reduced to CO_2_^2−^, is exergonic by ∆*G* = −4.1 kcal mol^−1^ (Figure 4). The influence of phenol on CO_2_ binding can best be quantified by the energy difference of −3.5 kcal mol^−1^ between **[1‐Co‐CO_2_]^o^** and **[1‐Co‐CO_2_]^o^_rear_**_._ (see Figures 4 and S52b). The former refers to the phenol cluster loosely bound to the Co centre on the opposite side of the CO_2_ and the latter to the phenol cluster hydrogen bonded to CO_2,_ both on the same side of the complex. In **[1‐Co‐CO_2_]^o^_rear_**,_._ the bound substrate interacts via hydrogen bonding with one of the present phenol molecules. The same phenol can readily transfer its proton to the carboxylate group (with a low energy barrier of <1 kcal mol^−1^) and the resulting phenolate is then stabilized by three neighbouring phenol molecules, one of which is polarized by a hydrogen bond to the NH group of the Hbbpya ligand.

The PhOH network also helps in the subsequent protonation of [**(Hbbpya)Co^II^‐CO_2_^2−^**]. If only a single PhOH acts as proton source, the reaction is predicted to be endergonic by Δ*G* = +5.2 kcal mol^−1^. After one proton is transferred, we assume that the PhOH group is readily recharged by either the excess PhOH or water that is being formed during the reaction. The subsequent second protonation step that leads to the formation of [**(Hbbpya)Co^II^‐CO**]^2+^ and water is again facilitated by a phenol‐mediated proton transfer to the CO_2_H group. However, owing to the C–O bond breaking this step is associated with a reaction barrier of ∆*G* ^‡^ = 6.0 kcal mol^−1^. At this point, we would like to emphasize that our results rather provide a “proof of principle” for the sequential proton transfer, stabilization of the phenolate, and the involvement of the NH moiety as an external polarizer and directing group. Of course, larger networks of phenols as well as networks that also involve water molecules (which are progressively formed during the CO_2_ reduction reaction) can facilitate the proton transfer. In [**(Mebbpya^.−^)Co^I^**]^0^, in the absence of any anchor for a H‐bonded phenol network, stepwise electron transfer and proton transfer events take place en route to CO formation (Scheme 2, bottom), which make the kinetics slower and the whole process less efficient. Furthermore, both CO_2_ and H^+^ compete for binding to the Co centre, leading to the observed loss of selectivity.

## Conclusion

In nature, the enzyme CO‐dehydrogenase (CODH) catalyzes the selective and reversible (2e^−^ + 2H^+^) conversion of CO_2_ to CO, whereby efficient CO_2_ reduction and protonation at a binuclear NiFe cluster is mediated by hydrogen bonding interactions from appropriately positioned amino acid residues, as revealed by structural studies of the active site.^[^ ^2^, ^67^, ^68^ ^]^ Pendant proton donors also facilitate catalysis of HER in nature, for example hydrogen evolution at the diiron site in FeFe‐hydrogenase.^[^ ^2^, ^69^ ^]^ These studies have inspired the development of biomimetic CO_2_RR and HER catalysts that involve a transition‐metal centre surrounded by ligands with pendant proton donors.^[^ ^27^, ^69^, ^70^, ^71^, ^72^, ^73^, ^74^, ^75^, ^76^, ^77^ ^]^ In the present report of the electrocatalytic CO_2_RR mediated by [**(Hbbpya)Co^II^**]^2+^, we demonstrate the combined role of ligand non‐innocence and secondary H‐bonding effects in controlling the two versus one‐electron reduction of CO_2_. The reaction of the active [**(Hbbpya^• –^)Co^I^**]^0^ species with CO_2_ leads to the formation of the two‐electron oxidation product **[(Hbbpya)Co^II^‐CO_3_^2−^]**, with the concomitant release of CO. The two protonation steps necessary for the release of CO occur very fast from PhOH to CO_2_ and mediated by an efficient H‐bonding network involving a network of PhOH moieties and ‐NH group of the Hbbpya ligand, as corroborated by the relatively lower PhOH/PhOD KIE of 1.11. Notably, consistent with our proposed mechanism, a similar KIE of 1.4 was determined for a pendant‐amine assisted intermolecular proton transfer from trifluoroethanol under homogeneous condition to a Co‐CO_2_H moiety to form CO,^[^ ^72^ ^]^ and a H‐bonded phenol is shown by DFT calculation to facilitate easy proton delivery and reduction of CO_2_ at an iron(I) porphyrinate center to form CO and water efficiently.^[^ ^78^ ^]^ The absence of any H‐bonding network in [**(Mebbpya^• –^)Co^I^**]^0^, in contrast, raises the barrier for CO_2_ binding, so that the formation of cobalt‐hydride competes with CO_2_ binding, as evidenced by the large PhOH/PhOD KIE of 8.5 and formation of both H_2_ and CO products. Furthermore, one‐electron reduction of CO_2_ occurs to form [**(Mebbpya)Co^I^‐CO_2_^•−^**], which under stoichiometric conditions releases oxalate ion in an aprotic medium. The CO_2_RR mechanism in [**(Hbbpya)Co^II^**]^2+^ is, therefore, reminiscent of the proposed two‐electron reduction of CO_2_ at the Ni(0)Fe(II) center in NiFe‐CODH to form Ni^II^‐(CO_2_^2−^)Fe^II^, where the CO_2_^2−^ moiety is stabilized by H‐bonding interactions involving the protein residues.^[^ ^68^ ^]^ It provides a relevant model for biological systems, and offers a framework for tuning the effect of the second coordination sphere on CO_2_ reduction, and more generally, on multielectron, multiproton reduction reactions. In addition, [**(Mebbpya)Co^II^**]^2+^ represents a unique system where a stepwise one‐electron transfer to the CO₂ molecule generates a transient [M‐CO₂•⁻] intermediate, subsequently leading to the formation of a C─C coupled product, oxalate. The knowledge gained from this work holds significant potential for guiding the rational design of molecular catalysts for C─C bond formation, a key focus of ongoing investigations in our laboratory.

## Supporting Information

The authors have cited additional references within the Supporting Information^[^ ^54^, ^61^, ^79^, ^80^, ^81^, ^82^, ^83^, ^84^, ^85^, ^86^, ^87^, ^88^, ^89^, ^90^, ^91^, ^92^, ^93^, ^94^, ^95^, ^96^, ^97^, ^98^, ^99^, ^100^, ^101^, ^102^, ^103^, ^104^, ^105^, ^106^, ^107^, ^108^, ^109^ ^]^ Additional details on the DFT calculations including optimized coordinates are available via the following link. https://scm.cms.hu‐berlin.de/roemelt_group_research_data/bera_acie_2025

## Conflict of Interests

The authors declare no conflict of interest.

## Supporting information



Supporting information

## Data Availability

The data that support the findings of this study are available in the Supporting Information of this article.
