# Supplementary material for: Mechanistic Promiscuity in Cobalt‐Mediated CO2 Reduction Reaction: One‐ Versus Two‐Electron Reduction Process
Source: Angew Chem Int Ed Engl. 2025 Jun 30;64(31):e202503705. doi: 10.1002/anie.202503705 (PMC12304851; doi:10.1002/anie.202503705)
Supplement: Supplementary file 1 — Supporting information [file ANIE-64-e202503705-s001.pdf]

Supporting Information

Mechanistic Promiscuity in Cobalt Mediated CO_2 Reduction Reaction: One- versus Two-Electron Reduction Process

Ayan Bera,[a] Sarah Bimmermann,[b] Philipp Gerschel,[b] Dibya Jyoti Barman,[a] Leon Gerndt,[a] Thomas Lohmiller,[a] [c] Kaltum Abdiaziz,[d] Alexander Schnegg,[d] Maylis Orio,[e] Dennis G. H. Hetterscheid,[f] Kara L. Bren,[g] Michael Roemelt,*[a] Ulf-Peter Apfel,*[b] [h] and Kallol Ray*[a]

Table of contents

1. Experimental section 3

1.1 Materials 3

1.2 Instrumentation and experimental methods 3

2 Synthesis of the ligands 7

2.1 Scheme S1: Synthesis of N, N-bis(2,2'-bipyrid-6-yl) amine (Hbbpya) 7

2.2 Scheme S2: Synthesis of N, N-bis(2,2'-bipyrid-6-yl) amide(bbpya) 8

2.3 Scheme S3: Synthesis of N-([2,2'-bipyridin]-6-yl)-N-methyl-[2,2'-bipyridin]-6-amine (Mebbpya) 9

3 Synthesis of the complexes 9

3.1 Synthesis of $[(Hbbpya)Co^{II}]^{2+}$: 9

3.2 Synthesis of $[(Hbbpya)Zn^{II}]^{2+}$: 10

3.3 Synthesis of $[(Mebbpya)Co^{II}]^{2+}$: 10

3.4 Synthesis of $[(Mebbpya)Zn^{II}]^{2+}$: 11

4 Scheme S4: Schematic overview of the electronic structures of the complexes formed upon sequential reduction of $[(Hbbpya)Co^{II}]^{2+}$ 11

5 Scheme S5: Schematic overview for the reaction of $[(Hbbpya^{\bullet-})Co^{I}]^{0}$ /$[(bbpya^{-})Co^{I}]^{0}$ with CO_2 . 11

6 Supplementary Figures 12

7 Supplementary Tables 54

8 DFT calculations 58

9 References 74

1. Experimental section

1.1 Materials

The chemicals employed were purchased from the companies ABCR, ACROS, SIGMA-ALDRICH, TCI, Alfa Aesar, BLDpharm and used without further purification. Anhydrous solvents (acetonitrile, dimethyl formamide, diethyl ether, THF, butyronitrile, dichloromethane) were purchased from CARL-ROTH GmbH under the tradename ROTIDRY (>99.5%, < 50 ppm H_2O) and degassed by freeze-pump-thaw methods and dried over activated molecular sieve (3 Å or 4 Å) prior to use. Dried and degassed acetone was purchased from THERMO SCIENTIFIC under the trade name ACRO SEAL (>99.8%, extra dry). Deuterated solvents were purchased from EURISO-TOP. Preparation and handling of air or water sensitive compounds were performed under an inert atmosphere using either Schlenk techniques or a glovebox GS111717 filled with N_2 from GS GLOVEBOX SYSTEMTECHNIK. Argon (Ar), nitrogen (N_2), and carbon dioxide (CO_2) of quality 5.0 were used for this purpose and were purchased from AIR LIQUIDE.

1.2 Instrumentation and experimental methods

Attenuated total reflection Fourier-transform infrared spectroscopy (ATR-FTIR). IR spectra were measured at an AGILENT Cary 630 FTIR spectrometer using a either a Diamond ATR sampling accessory with a type IIa diamond crystal or a DialPath sampling accessory equipped with ZnSe optical windows with a pathlength of 30, 50 or 1000 microns (depending on the measurement).

UV-Vis spectroscopy. The UV-Vis absorption spectra were recorded with an 8453 UV-Visible Spectroscopy system from Agilent. The measurements were carried out in 10 mm precision cuvettes made of SUPRASIL® quartz glass, the closures of which were equipped with a septum. The measurements at low temperatures were carried out by cooling the cuvette holder using a cooling thermostat USP-203-A from Unisoku Scientific Instruments. The analysis of the spectra was carried out with the software UV-Visible Chemstation from Agilent.

Stopped-flow UV-Vis spectroscopy. Stopped-flow UV-Vis measurements were performed with an SFM4000 from BioLogic SAS using a cryo-stopped-flow set-up, which is equipped with a HamatsuL10290 high power UV/vis fiber light source and a TIDAS S 300K VIS/NIR 3011 spectrometer. Instrument tuning and data handling were performed with BioKine Version 4.73 supplied by Biologic. The pathlength of the installed cuvette is 1 mm. The dead time lies in between 1 to 3 ms depending on flow rate (8–12 ml/s) and integration time.

Elemental analysis. All elemental analyses were performed by the analytical service of the Institut für Chemie of the Humboldt-Universität zu Berlin. The percentages of Carbon, Hydrogen, Nitrogen and Sulphur were determined using an HEKAtech EURO EA 3000 analyzer. The reported values are the result of an average of two independent measurements.

EPR spectroscopy. CW EPR spectra were collected by using a Bruker EMXplus Instrument at a frequency of ca. 9.35 GHz (X-Band) in perpendicular mode. All samples were measured at an average temperature of 13 K as frozen solutions (powder spectra) by the use of a liq. helium recirculating cooling system provided by ColdEdge. Spin-Hamiltonian-based simulations of EPR spectra were performed using the EasySpin toolbox.[1] Quantification of the concentration of the paramagnetic species against a copper(II) standard of known concentration was carried out by calculation of the respective double integrals upon polynomial baseline correction.

Nuclear magnetic resonance spectroscopy. All NMR spectra were recorded at 20 °C using BRUKER NMR spectrometer (Avance DPX 300 MHz, Avance 400 MHz, Avance III 500 MHz) equipped with a cryostat. ^{1}H and ^{13}C NMR were recorded in deuterated solvents and chemical shifts (δ, ppm) referenced against residual protic solvent peaks. ^{13}C NMR which were recorded in D_2O is referenced using DSS (4,4-dimethyl-4-silapentane-1-sulfonic acid, δ = 0 ppm). The ^{19}F NMR is referenced with respect to trichlorofluoromethane ($CFCl_3$).

Electrospray ionization mass spectrometry. ESI-MS spectra in solution were recorded by using an ADVION EXPRESSION CMS spectrometer; acetonitrile was used as an eluent and the sample was directly injected into the instruments. The analysis of the data was carried out with the ADVION DATA EXPRESS Version 6.0.11.3. Simulation of the isotopic pattern of the experimental base peak was performed by the use of ISOTOPE DISTRIBUTION CALCULATOR AND MASS SPEC PLOTTER software provided by SIS (Scientific Instrument Services).

Single crystal X-ray structure determinations. Data collections were performed at 100 K on a BRUKER D8 VENTURE diffractometer by using Mo Kα radiation (λ = 0.71073 Å). The structure was solved with the XT[2] solution program using Intrinsic Phasing methods and by using Olex2 1.5[3] as the graphical interface. The model was refined with XL using full matrix least squares minimisation on F^2.

Spectro-Electrochemistry. Spectro-electrochemical measurements were performed with a Metrohm Autolab PGSTAT 204 potentiostat/Galvanostat equipped with a Pt net as a working electrode, Glassy carbon as counter electrode and Ag wire as pseudo-reference electrode. The supporting electrolyte tetrabutylammonium hexafluorophosphate (>99 %, Sigma) was used as received.

UV-Vis absorption Spectro electrochemistry measurements in parallel configuration (the light beam passes parallel and close to the electrode surface) were performed using a AUTOLAB Spectrophotometer UA in the UV/VIS/NIR wavelength range (from 200 nm to 1100 nm). UV-Vis spectrometer was properly synchronized with the Metrohm AUTOLAB PGSTAT 204 potentiostat. All the UV-Vis data were supported by AVANTES AVASOFT 8.11 and the Electrochemical data were handled by NOVA 2.1.5 Software.

The light beam, supplied by a light source (AUTOLAB D/HAL Light Source, METROHM), was conducted to the Spectro electrochemical cell by a 200 µm bare optical fiber (METROHM), and collected from the Spectro electrochemical cell to the spectrometer by a 200 µm bare optical fiber. (The standard dimensions of the optical fibers is 2 m in length and 200 µm in diameter. The fibers are fitted with SMA-905 connectors on both ends).

Spectro-electrochemical EPR experiments. The sample was first subjected to the electrochemical process in a glovebox (H_2O < 0.5 ppm, O_2 < 0.5 ppm), and then removed from the electrochemical cell for EPR spectroscopic analysis. The electrochemistry (Biologic Potentiostat SP-300) was performed in a standard electrochemical two-compartment setup with a glass frit separating the two compartments. The potential bias, chosen from the cyclic voltammogram of the complexes, was applied for 5 min or until a colour change was observed. Once achieved, the sample (400 µL) was transferred to an EPR tube (O.D 4.0 mm, I.D 2.8 mm), frozen and transferred to the spectrometer for EPR measurements. The working electrode consisted of a gold wire (diameter – 1 cm, length – 25 cm) with the lower 3 cm flattened to increase its surface area, and the remaining wire was insulated using heat shrink PTFE tubing. The active working area has an overall surface area of 1.22 cm². The counter electrode was a coiled platinum wire with diameter – 1 cm, length – 25 cm and the pseudo-reference was an insulated Ag wire. The working electrode, reference electrode and the electrolyte with the complex were placed in one compartment and the counter electrode with electrolyte in the other compartment. For cyclic voltammogram measurements, all the electrodes were placed in one compartment with the electrolyte and complex. X-Band continuous wave (CW) EPR measurements were performed using a Bruker E500 ELEXSYS spectrometer equipped with a Bruker dual-mode resonator (ER 4116DM, Bruker, Germany),

Oxford Instruments helium flow cryostat (ESR 900) and Mercury iTC temperature controller (Oxford Instruments, UK).

Controlled-potential coulometry (CPC). CPC measurements were performed for 8h at defined potentials in a sealed one-compartment cell using a PalmSens3 or PalmSens4 potentiostat. The electrolyte consists of 0.5 mM complex, 3 M phenol and 0.1 M $[(^nBu)_4N]PF_6$ in acetonitrile. A standard three-electrode set-up using a glassy carbon electrode as working electrode, a Pt wire as counter electrode and an Ag wire as pseudo-reference electrode. The working electrode was polished using 1.0, 0.3 and 0.1 μm sandpaper and sonicated for 10 min. Prior to the electrolysis the cell was degassed for 10 min and the electrolyte solution was purged for 10 min using CO_2. The electrochemical cell contained 2 mL of electrolyte solution and a headspace of 61.6 mL, 71.1 mL, 67.7 mL, 62.2 mL, 65.9 mL or 57.7 mL.

Quantification of the product. Quantification of the headspace gas composition of the electrochemical cell was performed using a Shimadzu GC-2010 Pro equipped with a Shimadzu BID-2010 Plus barrier discharge ionization detector (BID). Gas separation was performed *via* hand injection using a SPL injector. A sH-Rxi-1ms fused silica capillary GC column (L x LD 30 m x 0.32 mm, average thickness 1.0 μm) was used to separate solvent and gases. The gases were further separated using a Carboxen 1010 PLOT fused silica capillary GC column (L x LD 30 m x 0.32 mm, average thickness 15 μm). Helium was used as carrier gas and the product amount was determined by a calibration curve. The following gases products were assayed *via* the GC-BID system: H_2, O_2, N_2, CO, CH_4 C_2H_4 and C_2H_6.

Quantification of the liquid phase for formate/formic acid of the electrochemical experiments were performed using a Shimadzu GCMS-QP2020 system equipped with as MS detector and a Shimadzu HS-20 autosampler. Helium was used as carrier gas for all measurements. For sample analysis of the formate/formic acid, derivatization to the corresponding ester was performed. Therefore, 400 μL sample, 500 μL n-propanol and 100 μL of a 10% aqueous solution of p-toluenesulfonic acid were heated to 60 °C for 10 min. The samples were pressurized using helium at 200 kPa for 1 min and injected onto a SH-Rtx 200 ms fused silica capillary GC column (L × I.D. 30 m × 0.25 mm, average thickness 1 μm) with a constant helium pressure of 110 kPa, a split ratio of 1:70 and isothermally separated at 70 °C for 6 min. The temperature of the interface of the MS was 200 °C and of the ion source 230 °C. The concentrations of the liquid phase were calibrated by using standard solutions with defined concentrations. The obtained data were analyzed with the Shimadzu GCMS Postrun Analysis software.

The Shimadzu GC-2014 gas chromatograph was used for CO or H_2 detection after the stoichiometric reaction of the catalyst with a thermal conductivity detector and a Resteks ShinCarbon packed column ST 80/100(2 m, 1/8" outer diameter, 2 mm inner diameter). The injector temperature was set to 200 °C, the detector temperature set to 300 °C and the gases were separated according to a temperature-time program on the column. CO and persistent gases such as Ar and N_2 were separated at the early stages of the measurement at an oven temperature of 40 °C and after 20 min CO_2 was eluted at the later stages.

Kinetic Isotope Effect (KIE) determination. For the determination of the KIE values a procedure described by Artero and co-workers[4] was used. Therefore, the i_{cat}/i_p was plotted against the concentration of PhOH and PhOD measured during a CV at 100 mV s^{-1}.

Since, Electrochemical rate constants could be derived from the formula $\frac{i_{cat}}{i_P} = 2.24 \times n\sqrt{\frac{RTk_{obs}}{Fv}}$

Where, k_{obs} is the rate constant; n is the number electron transferred in one catalytic turnover, and is equal to 2 for CO_2 to CO; F is Faraday's constant (F= 96500 C mol^{-1}); R is the universal gas constant (R = 8.314 J K^{-1} mol^{-1}), T is temperature (298 K) and ν is the scan rate.

Since, $(k_{obs})^{1/2}$ is related to i_{cat}/i_p, the slopes of PhOH/PhOD plots are related to KIE by the equation,

$$\text{KIE} = \frac{k_{PhOH}}{k_{PhOD}} = \left(\frac{slope_{PhOH}}{slope_{PhOD}}\right)^2$$

Generation of Reduced Species and Sample Preparation. In a typical experiment, 1 mM solution of **[(Hbbpya)Co^{II}]$^{2+}$** or **[(Mebbpya)Co^{II}]$^{2+}$** in MeCN or, butyronitrile was taken in an air-tight UV-Vis cuvette. Requisite amounts of reductant ($CoCp_2$ or $CoCp_2^*$) were added to generate different reduced intermediates and followed by UV-Vis spectroscopy. After confirming the generation of the different reduced intermediates, the samples were transferred to an EPR tube under Ar and frozen immediately in liq. N_2 for the measurement of EPR spectroscopy.

Stoichiometric reaction of CO_2 by [(Mebbpya)Co^{II}]$^{2+}$.

A 20 mL Schlenk flask was charged with 5 mL MeCN and 40 mg (0.054 mmol) of **[(Mebbpya)Co^{II}]$^{2+}$** inside a N_2 filled glovebox. Then, 2.2 equiv. of KC_8 (16 mg, 0.11 mmol) or, $CoCp^*_2$ (38.8 mg, 0.11 mmol) with KPF_6 (21.7 mg, 0.11 mmol) was added to it. The whole reaction was stirred for 60 min inside the glovebox. Afterwards the reaction mixture was filtered through a plug of celite and transferred to another Schlenk flask. The Schlenk flask was removed from the glovebox and attached to a vacuum manifold. After subjecting the solution to three freeze-pump-thaw cycles, the reaction mixture was exposed to 1 atm. of dried CO_2 for 2 min at room temperature, which resulted in the immediate generation of a precipitate. The stopcock valve was occasionally opened to CO_2 flow to equilibrate the internal pressure in the flask. The reaction was allowed to stir under static CO_2 atmosphere for 24h. The reaction flask was returned to the glovebox, filtered through a ground glass fritted funnel, and the filtrate was collected. The solid precipitate was extracted with distilled water. After removing water under vacuum, the residue was assayed by ^{13}C-NMR and IR spectroscopy. For the quantification of oxalate, the precipitate was treated with 1 M HCl to form the oxalic acid and titrated with standardised $KMnO_4$.

2 Synthesis of the ligands

2.1 Scheme S1: Synthesis of N, N-bis(2,2'-bipyrid-6-yl) amine (Hbbpya)

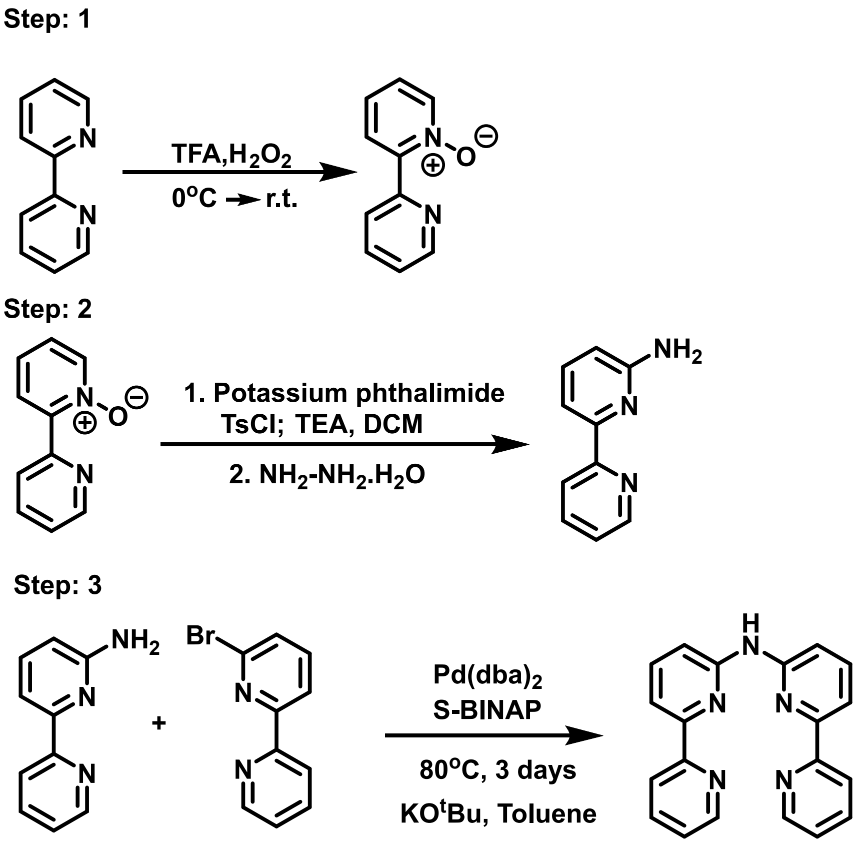

Step 1: 2,2'-bipyridine N-oxide[5-6]

2,2'- bipyridine (10 g, 63.4 mmol, 1 equiv.) was dissolved in 50 mL trifluoracetic acid and cooled to 0 °C. H_2O_2 (35 wt%, 6 mL, 69.7 mmol, 1.1 equiv.) was added dropwise to the solution at 0 °C. The whole reaction mixture was stirred for 24h at room temperature. Subsequently, the whole mixture was neutralized with NaOH bead up to a pH of 9~10. The aqueous phase was extracted multiple times with $CHCl_3$. The combined organic phase was dried with Na_2SO_4, filtered and concentrated in vacuo to yield the product as a white powder (10.7 g, 62.2 mmol, 98%). ^{1}H NMR (500 MHz, $CDCl_3$) δ 8.90 (ddd, J_1 = 8.1, $J_2 \approx J_3$ = 1.1 Hz, 1H, H1), 8.73 (ddd, J = 4.8, 1.8, 0.9 Hz, 1H, H4), 8.32 (dd, J = 6.5, 1.3 Hz, 1H, H8), 8.19 (dd, J = 8.1, 2.2 Hz, 1H, H5), 7.84 (ddd, appearing as pseudo td, $J_1 \approx J_2$ = 7.6, J_3 = 1.8 Hz, 1H, H2), 7.40 – 7.33 (m, 2H, H3, H6), 7.30 – 7.26 (m, 1H, H7).

Step 2: 6-amino-2,2'-bipyridine[7]

Bipyridine-N-oxide (2.49 g, 0.014 mol, 1 equiv.), Potassium Phthalimide (5.357 g, 0.018 mol, 2 equiv.) and triethyl amine (4.8 mL, 2 equiv.) were dissolved in 60 mL dry and degassed DCM under N_2. The whole suspension was cooled to 0 °C using an ice bath. Tosyl chloride (5.509 g, 0.0289 mol, 2 equiv.) was added to the mixture portion-wise with a span of 30 min. The temperature was strictly maintained at 0 °C during the addition of tosyl chloride. Once the addition of tosyl chloride was finished, the ice bath was removed and the whole mixture was kept stirring for 3 days. After 3 days, the conversion was checked by TLC with respect to the starting bipyridine-N-oxide. If the conversion was not finished, it was kept stirring for one day more (it will turn light orange). Once the reaction was completed by TLC, the whole residue was dried in vacuo.

Now, hydrazine hydrate (80%) (3 mL, 5 equiv. with respect to bipyridine-N-oxide) was added to it. The whole mixture was diluted by adding 80 mL of distilled water. The suspension was stirred at 80 °C for 12h, when it will turn to a light red clear solution. This was extracted by $CHCl_3$ for 3 times. All the organic phase was collected and washed again with 100 mL 1 M NaOH and then it was dried over Na_2SO_4 and filtered. Evaporation of the solvent yielded the desired product (97 % yield). ^{1}H NMR (500 MHz, $CDCl_3$) δ 8.66 (ddd, J = 4.8, 1.8, 0.9 Hz, 1H, H1), 8.27 (ddd, appearing as pseudo dt, J_1 = 8.0, $J_2 \approx J_3$ = 1.1 Hz, 1H, H4), 7.78 (ddd, appearing as pseudo td, $J_1 \approx J_2$ = 7.7, J_3 = 1.8 Hz, 1H, H3), 7.71 (dd, J = 7.6, 0.9 Hz, 1H, H5), 7.58 (t, J = 7.8 Hz, 1H, H6), 7.30 – 7.24 (m, 1H, H2), 6.55 (dd, J = 8.1, 0.9 Hz, 1H, H7), 4.62 (s, 2H, H8).

Step 3: N, N-bis(2,2′-bipyrid-6-yl) amine (Hbbpya)[8]

6-bromo-2,2'-bipyridine (0.50 g, 2.13 mmol, 1 equiv.) and $Pd(dba)_2$ (0.031 g, 0.054 mmol, 0.025 equiv.) were placed in a flame dried flask under a N_2 atmosphere. (S)-(-)-2,2'-bis(diphenylphosphino)-1,1'-binaphthyl (0.087 g, 0.140 mmol, 0.06 equiv.), KO^{t}Bu (1.07 g, 9.5 mmol, 4.5 equiv.) and 40 mL of dry and degassed toluene were added, and the mixture was stirred for 20 min. Subsequently, 6-amino-2,2'-bipyridine (0.43 g, 2.49 mmol, 1.2 equiv.) was added, and the resultant mixture was heated for 4 days at 80 °C, after which it was cooled down to room temperature and quenched with 20 mL of water. The mixture was stirred vigorously for 1 h and was then extracted three times with DCM. The combined organic layers were dried with Na_2SO_4, filtered and concentrated in vacuo resulting in a brown oil. Upon addition of cold MeOH, a brown solid precipitated from the brown oil. The solid was isolated by filtration, washed with cold MeOH and dried under vacuum at 40 °C to yield the desired product (0.39 g, 1.19 mmol, 56%). ^{1}H NMR (300 MHz, DMSO-d^6) δ 9.90 (s, 1H, H1), 8.70 (ddd, J = 4.7, 1.8, 0.9 Hz, 2H, H8), 8.39 (ddd, appearing as pseudo dt, J_1 = 7.8, $J_2 \approx J_3$ = 1.1 Hz, 2H, H5), 8.00 (ddd, appearing as pseudo td, $J_1 \approx J_2$ = 7.7, J_3 = 1.8 Hz, 2H, H7), 7.94 (ddd, appearing as pseudo dt, J_1 = 5.4, $J_2 \approx J_3$ = 2.7 Hz, 2H, H4), 7.90 (d, J = 2.6 Hz, 4H, H2-H3), 7.46 (ddd, J = 7.5, 4.7, 1.2 Hz, 2H, H6).

^{13}C NMR (75 MHz, DMSO-d^6) δ 155.94, 154.33, 153.90, 149.75, 139.19, 137.73, 124.46, 120.84, 113.31, 112.78.

2.2 Scheme S2: Synthesis of N, N-bis(2,2'-bipyrid-6-yl) amide(bbpya)

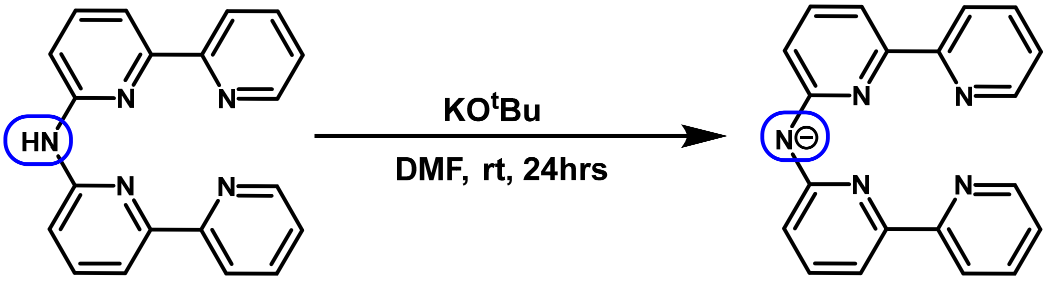

N, N-bis(2,2′-bipyrid-6-yl) amine (Hbbpya) (300 mg, 0.921 mmol, 1 equiv.) was dissolved in 10 mL dry and degassed DMF under Ar in flame dried round bottom flask. KO^{t}Bu (217 mg, 1.94 mmol, 2.1 equiv.) was added to the solution and the colour changed immediately to orangish-red, which was stirred under argon for 5h. DMF was removed in Schlenk under high vacuum and the resulting yellowish residue washed twice with Et_2O and hexane. The residue was dried overnight under high vacuum to yield orange powder of the desired product (276 mg, 0.86 mmol, 93.37%). ^{1}H NMR (300 MHz, DMSO-d^6) δ 8.60 – 8.52 (m, 2H, H7), 8.30 (d, J = 8.0 Hz, 2H, H4), 7.83 (ddd, appearing as pseudo td, $J_1 \approx J_2$ = 7.6, J_3 = 1.7 Hz, 2H, H6), 7.32 – 7.20 (m, 8H, H1,2,3,5).

^{13}C NMR (75 MHz, DMSO-d^6) δ 164.07, 158.10, 152.86, 148.53, 136.45, 135.12, 122.51, 119.90, 115.96, 105.34.

2.3 Scheme S3: Synthesis of N-([2,2'-bipyridin]-6-yl)-N-methyl-[2,2'-bipyridin]-6-amine (Mebbpya)

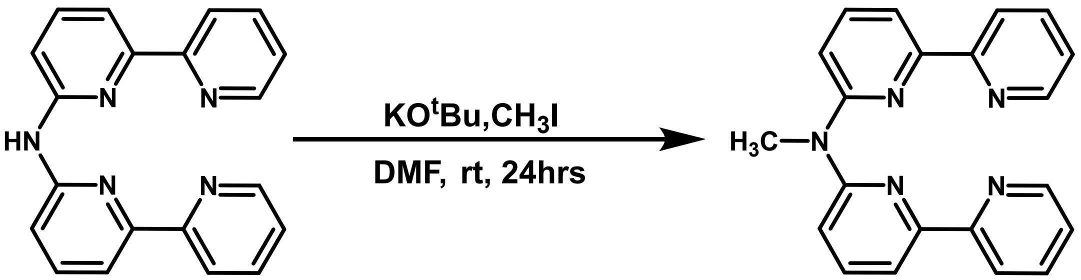

N, N-bis(2,2′-bipyrid-6-yl) amine (Hbbpya) (600 mg, 1.842 mmol, 1 equiv.), KO^{t}Bu (434 mg, 3.88 mmol, 2.1 equiv.) and CH_3I (564 mg, 0.2 mL, 3.77 mmol, 2.1 equiv.) were dissolved in 10 mL dry and degassed DMF and stirred for 24h at room temperature under Ar. Then the reaction solution was extracted with ethyl acetate and water. Afterwards, the organic phase was extracted with NH_4Cl solution. The combined organic layers were dried with Na_2SO_4, filtered and concentrated in vacuo resulting in a deep brown oil. This was further purified by alumina column chromatography with eluent hexane and ethyl acetate mixture (0 to 20 % ethyl acetate in hexane) to give a yield of 67%. ^1H NMR (300 MHz, MeOD) δ 8.58 (d, J = 4.9 Hz, 1H), 8.32 (d, J = 8.0 Hz, 1H), 7.91 – 7.82 (m, 2H), 7.76 (ddd, appearing as t, $J_1 \approx J_2$ = 7.9 Hz, J_3 = too small, 1H), 7.41 – 7.30 (m, 2H), 3.75 (s, 2H).

^{13}C NMR (75 MHz, MeOD) δ 157.73, 156.66, 154.38, 149.08, 138.63, 137.83, 124.25, 121.80, 115.06, 114.38, 93.18, 71.08, 26.92.

3 Synthesis of the complexes

3.1 Synthesis of [(Hbbpya)Co^{II}]$^{2+}$:

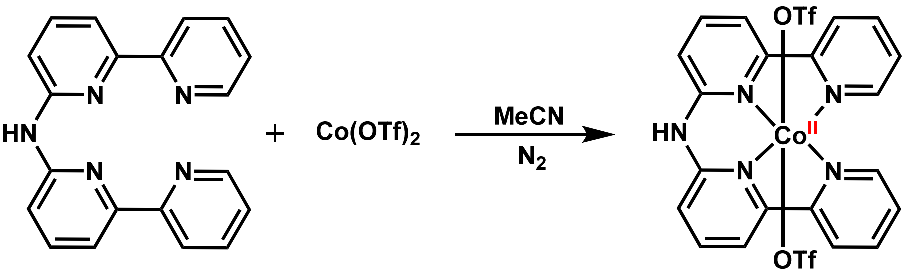

Hbbpya ligand (100 mg, 0.307 mmol, 1 equiv.) was dissolved in 5 mL dry acetonitrile inside the glovebox to which a suspension of $Co(OTf)_2$ (109.7 mg, 0.307 mmol, 1 equiv.) in acetonitrile was added. The mixture was stirred under nitrogen at room temperature for 12h. Afterwards, the clear deep yellow solution was filtered through a syringe filter and was added dropwise to 30 mL of diethyl ether. Addition to excess Et_2O led to precipitation of yellow solids, which were kept in the freezer at -25 °C for complete crystallisation for 5h. The suspension was filtered and the yellow solid was washed twice with Et_2O. The solid was dried under vacuum giving 160 mg of **[(Hbbpya)Co^{II}]$^{2+}$** (0.234 mmol, 77%). Single crystals of X-ray quality were grown by vapour diffusion of Et_2O in a saturated acetonitrile solution of **[(Hbbpya)Co^{II}]$^{2+}$** at -25 ^{0}C.

1H NMR (300 MHz, CD_3CN) δ 41.30 (s, 2H), 35.52 (s, 2H), 20.59 (s, 2H), 19.62 (s, 3H), 18.15 (s, 2H), 15.63 (s, 3H), -18.11 (s, 1H).

^{19}F NMR (282 MHz, CD_3CN) δ -78.03.

ESI-MS: (positive) calculated m/z = 233.0 for $[Co(Hbbpya)(CH_3CN)_2]^{2+}$, found m/z = 233.0 (100%).

Elemental Analysis: Found (calculated) for $C_{22}H_{15}CoF_6N_5O_6S_2$: C, 38.90 (38.72); H, 2.35 (2.22); N, 10.45 (10.26); F, 16.98 (16.70); O, 14.07 (14.30); S, 9.56 (9.40).

3.2 Synthesis of $[(Hbbpya)Zn^{II}]^{2+}$:

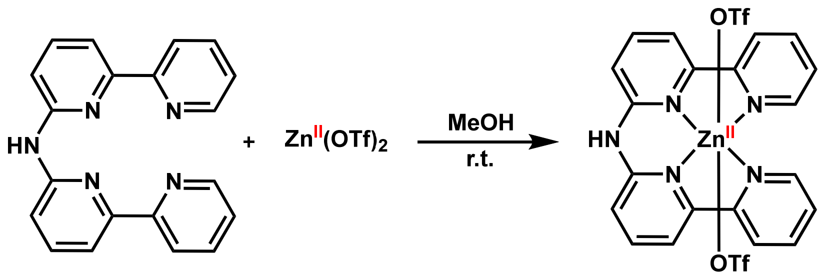

A solution of $Zn(OTf)_2$ (165 mg, 0.461 mmol, 1 equiv.) in 5 mL methanol was added to the Hbbpya solution (150 mg, 0.461 mmol, 1 equiv.). The resultant mixture was stirred overnight at room temperature followed by removal of the solvent in vacuo. The mixture was redissolved in a minimal amount of MeOH and crystals were grown by vapor diffusion with MeOH/Et_2O at 4 °C. The obtained crystals were isolated by filtration and were washed thoroughly with Et_2O. After drying the crystals at 40 °C in vacuo the target compound, **($[(Hbbpya)Zn^{II}]^{2+}$)** was obtained (245 mg, 0.368 mmol, 80%).

^{1}H NMR (300 MHz, CD_3CN) δ 9.57 (s, 1H), 9.05 (d, J = 5.2 Hz, 2H, H8), 8.57 (d, J = 8.2 Hz, 2H, H5), 8.36 (ddd, appearing as pseudo t , $J_1 \approx J_2$ = 7.9, J_3 = too small, 2H, H6), 8.23 – 8.11 (m, 4H, H3,4), 7.90 (ddd, appearing as pseudo t , $J_1 \approx J_2$ = 6.6 Hz, J_3 = too small, 2H, H7), 7.44 (d, J = 7.6 Hz, 2H, H2).

^{19}F NMR (282 MHz, CD_3CN) δ -80.14.

3.3 Synthesis of $[(Mebbpya)Co^{II}]^{2+}$:

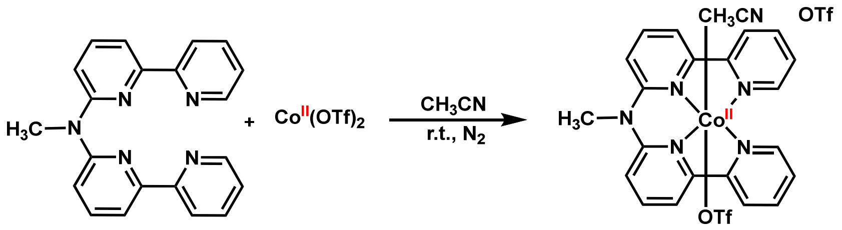

Mebbpya ligand (100 mg, 0.295 mmol, 1 equiv.) was dissolved in 5 mL dry acetonitrile inside the glovebox to which a suspension of $Co(OTf)_2$(105.2 mg, 0.294 mmol, 1 equiv.) in acetonitrile was added. The mixture was stirred under N_2 at room temperature for 12h. Afterwards, the clear deep yellow solution was filtered through a syringe filter. Et_2O was allowed to diffuse into the dark yellow filtrate overnight at -25 ºC and additional standing for 2 days at room temperature to yield dark yellow crystals of **$[(Mebbpya)Co^{II}]^{2+}$**(173 mg, 0.236 mmol, 80%). The identity of the product was confirmed by single crystal XRD.

^{1}H NMR (300 MHz, CD_3CN) δ 17.80 (s, 2H), 15.13 (s, 2H), 12.80 (d, J = 114.1 Hz, 4H), 11.93 (s, 2H), 3.65 (s, 3H), 3.03 (s, 2H).

^{19}F NMR (282 MHz, CD_3CN) δ -78.20.

ESI-MS: (positive) calculated m/z = 219.5 for $[Co(Mebbpya)(CH_3CN)]^{2+}$, found m/z = 219.5 (100%).

Calculated m/z = 240.1 for $[Co(Mebbpya)(CH_3CN)_2]^{2+}$, found m/z = 240.1 (15%).

Elemental Analysis: Found (calculated) for $C_{25}H_{20}CoF_6N_6O_6S_2$: C, 40.95 (40.71); H, 2.90 (2.73); N, 11.58 (10.40); F, 15.62 (15.46); O, 13.27 (13.02); S, 8.65 (8.69).

3.4 Synthesis of $[(\text{Mebbpya})\text{Zn}^{II}]^{2+}$:

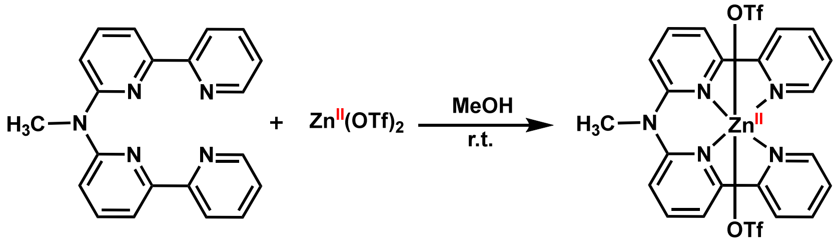

A solution of $Zn(OTf)_2$ (100 mg, 0.275 mmol, 1 equiv.) in 5 mL methanol was added to the Mebbpya solution (89 mg, 0.27 mmol, 1 equiv.). The resultant mixture was stirred overnight at room temperature followed by removal of the solvent in vacuo. The mixture was redissolved in a minimal amount of MeOH and crystals were grown by vapor diffusion with $MeOH/Et_2O$ at 4 °C. The obtained white crystals were isolated by filtration and were washed thoroughly with Et_2O. After drying the crystals at 40 °C in vacuo the target compound, **($[(\text{Mebbpya})\text{Zn}^{II}]^{2+}$)** was obtained (140 mg, 0.2 mmol, 74%).

^{1}H NMR (300 MHz, MeOD) δ 9.25 (ddd, J = 5.3, 1.6, 0.9 Hz, 2H, H8), 8.78 (ddd, appearing as pseudo dt , J_1 = 8.2, , $J_2 \approx J_3$ = 1.0 Hz, 2H, H5), 8.52 – 8.34 (m, 6H, H4,6,3), 8.01 (ddd, J = 7.6, 5.2, 1.1 Hz, 2H, H2), 7.82 (dd, J = 8.0, 1.2 Hz, 2H, H7), 3.85 (s, 3H, H1).

^{13}C NMR (75 MHz, MeOD) δ 156.75, 149.64, 148.80, 146.94, 143.10, 141.97, 127.15, 123.53, 117.95, 117.07, 48.45, 48.16, 47.88, 47.60, 47.31, 47.03, 46.74, 40.61.

4 Scheme S4: Schematic overview of the electronic structures of the complexes formed upon sequential reduction of $[(\text{Hbbpya})\text{Co}^{II}]^{2+}$

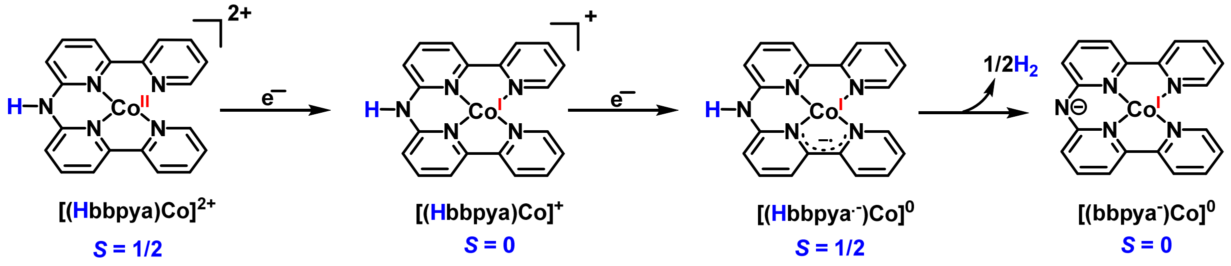

5 Scheme S5: Schematic overview for the reaction of $[(\text{Hbbpya}^{\bullet -})\text{Co}^{I}]^0$ /$[(\text{bbpya}^-)\text{Co}^{I}]^0$ with CO_2

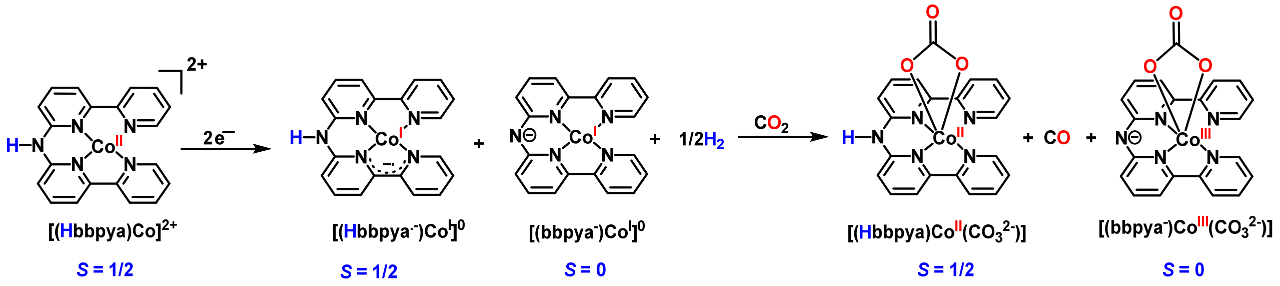

6 Supplementary Figures

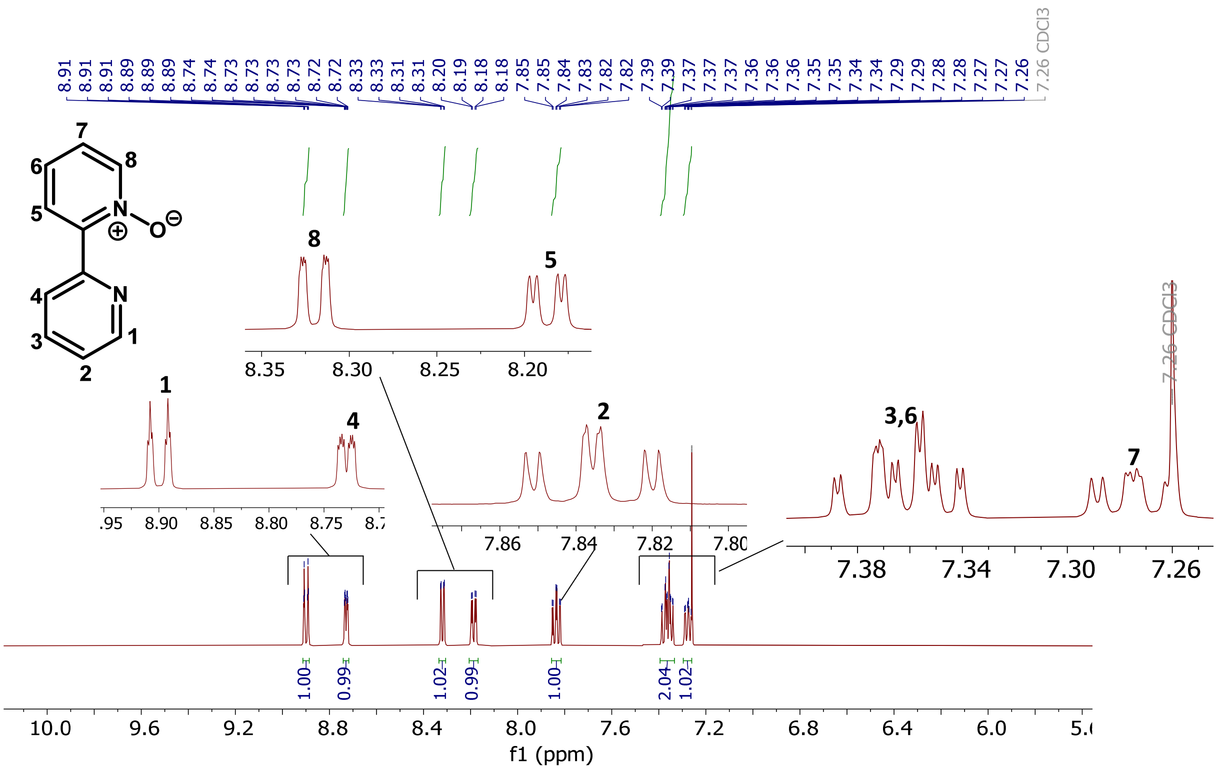

Figure S1. ^{1}H NMR of 2,2'-bipyridine N-oxide in $CDCl_3$ at 298 K.

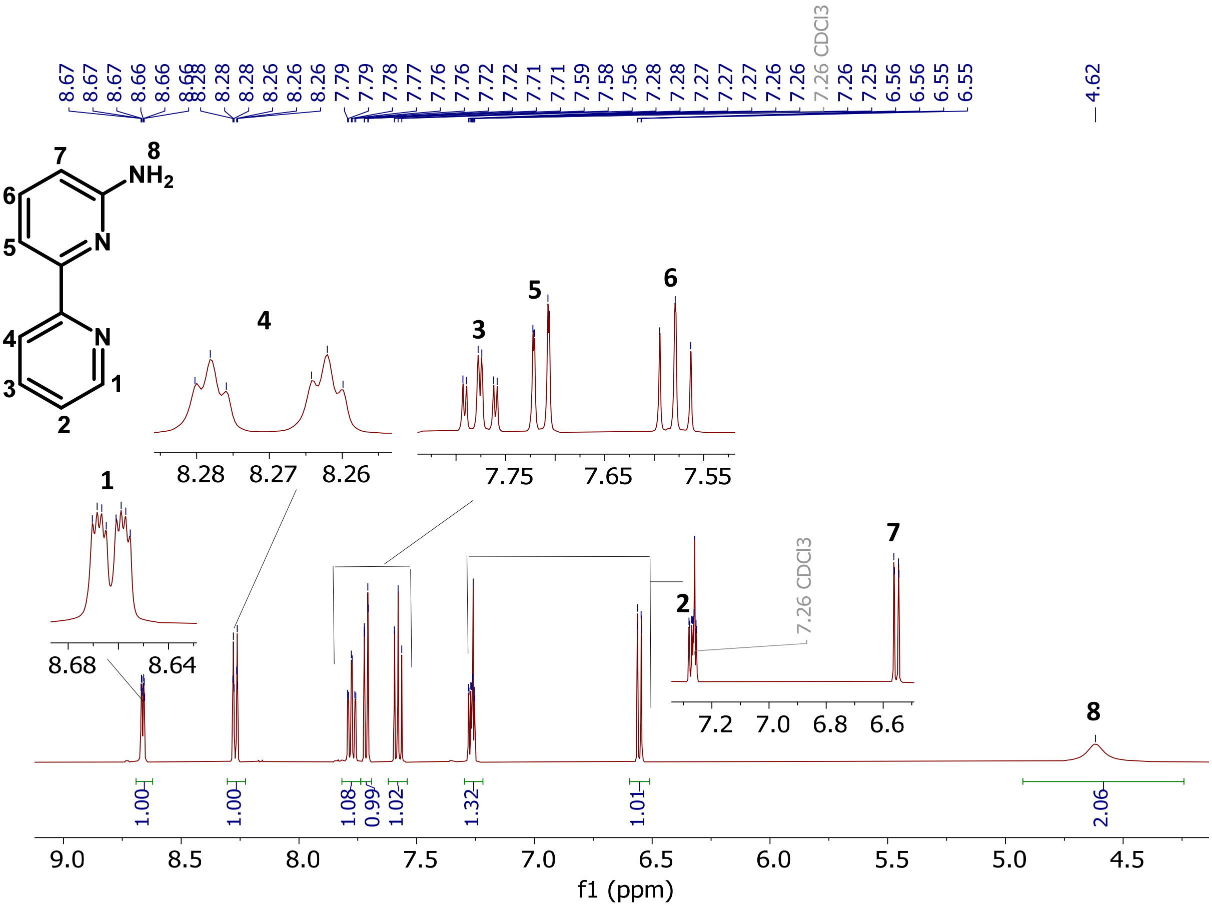

Figure S2. ^{1}H NMR of 6-amino-2,2’-bipyridine in $CDCl_3$ at 298 K.

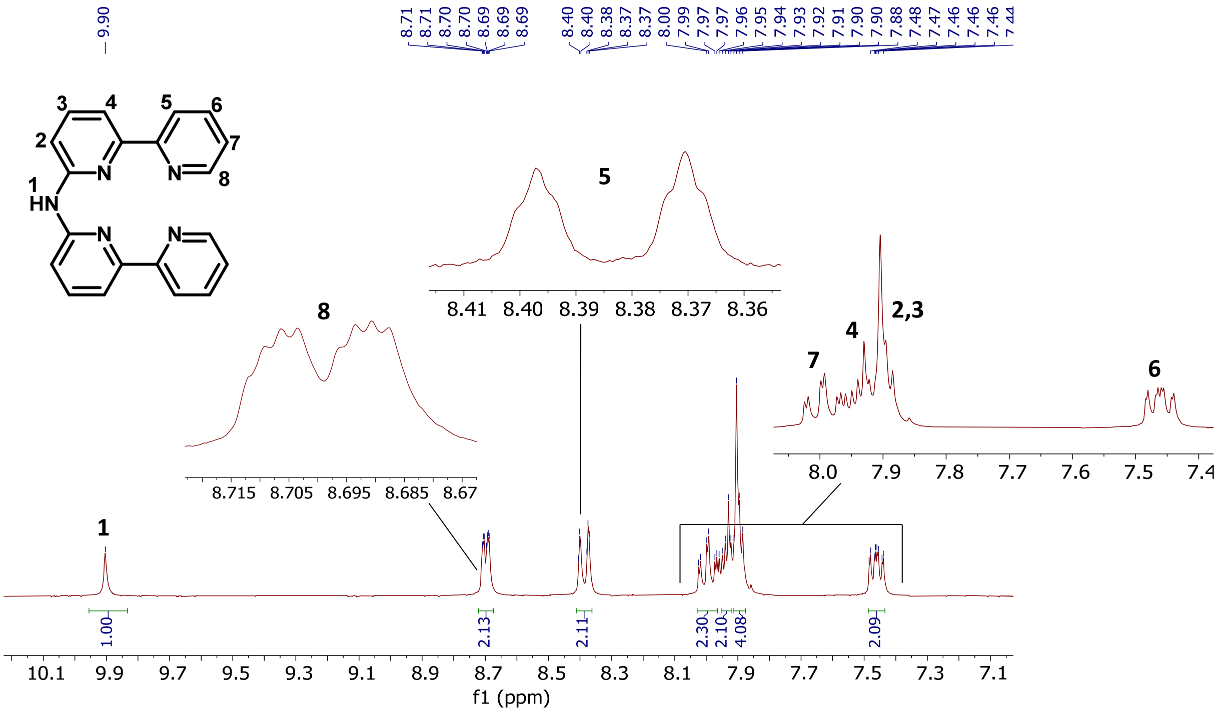

Figure S3. ^{1}H NMR of Hbbpya in DMSO-d^{6} at 298 K.

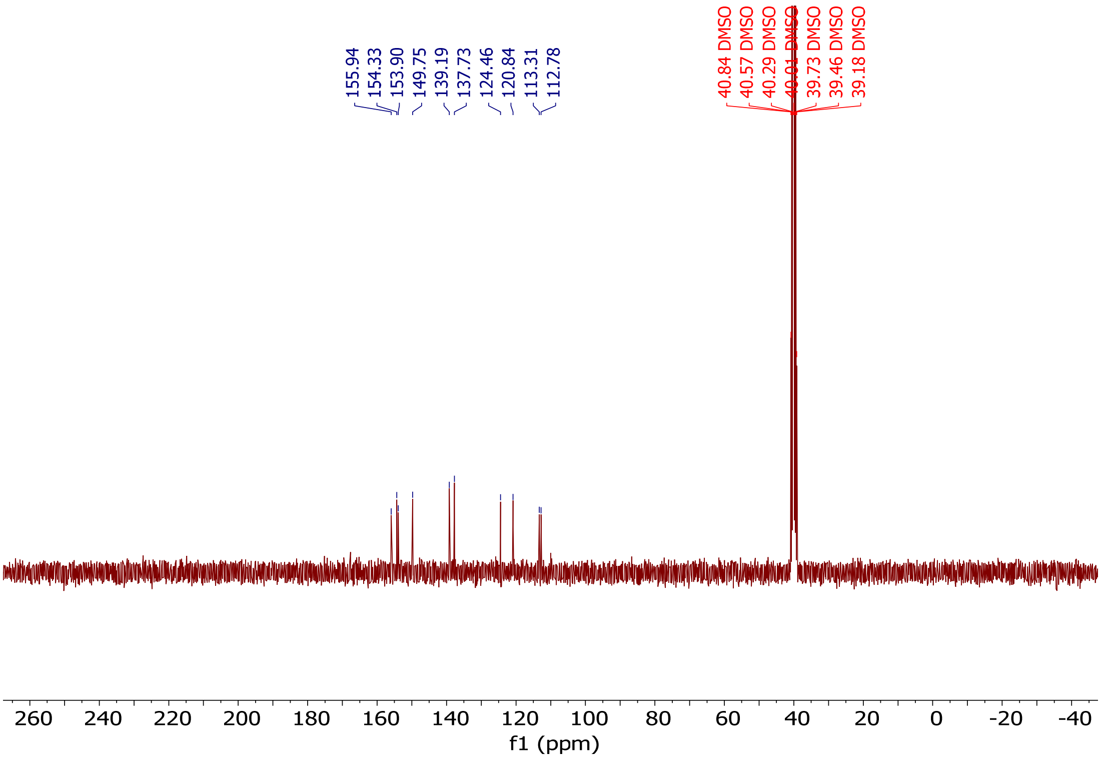

Figure S4. ^{13}C NMR spectrum of Hbbpya in DMSO-d^6 at 298 K.

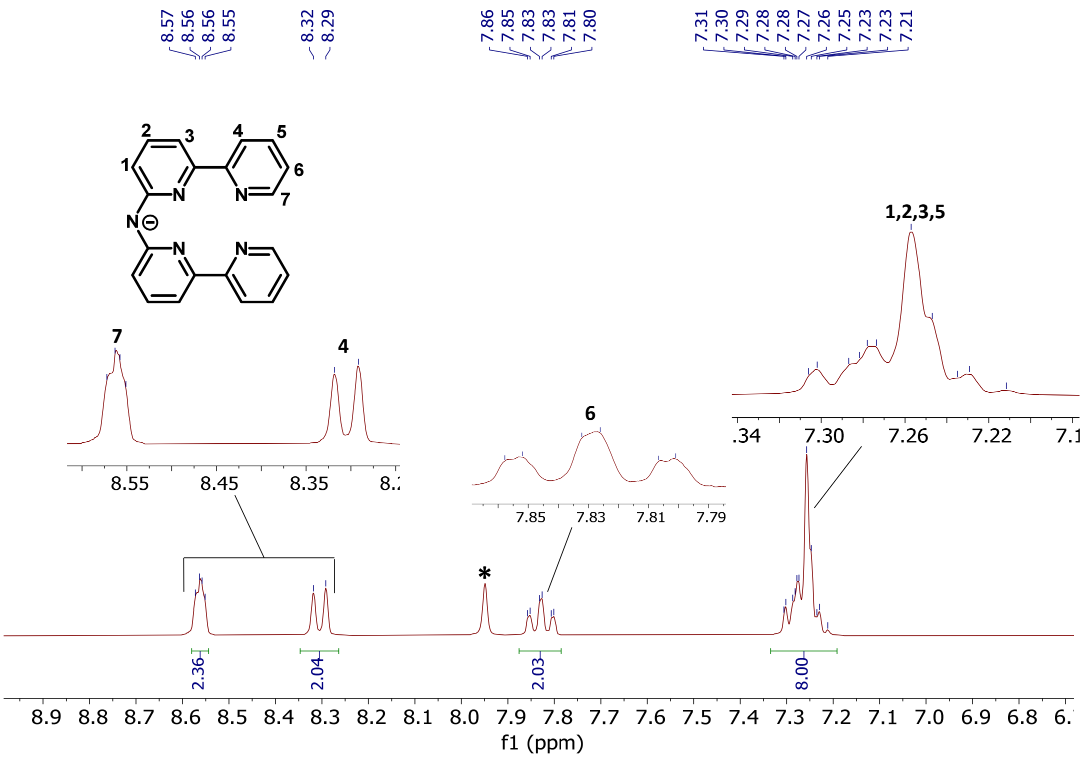

Figure S5. ^{1}H NMR of bbpya in DMSO-d^6 at 298 K. (*) residual -OH peak from the by-product ^{t}BuOH.

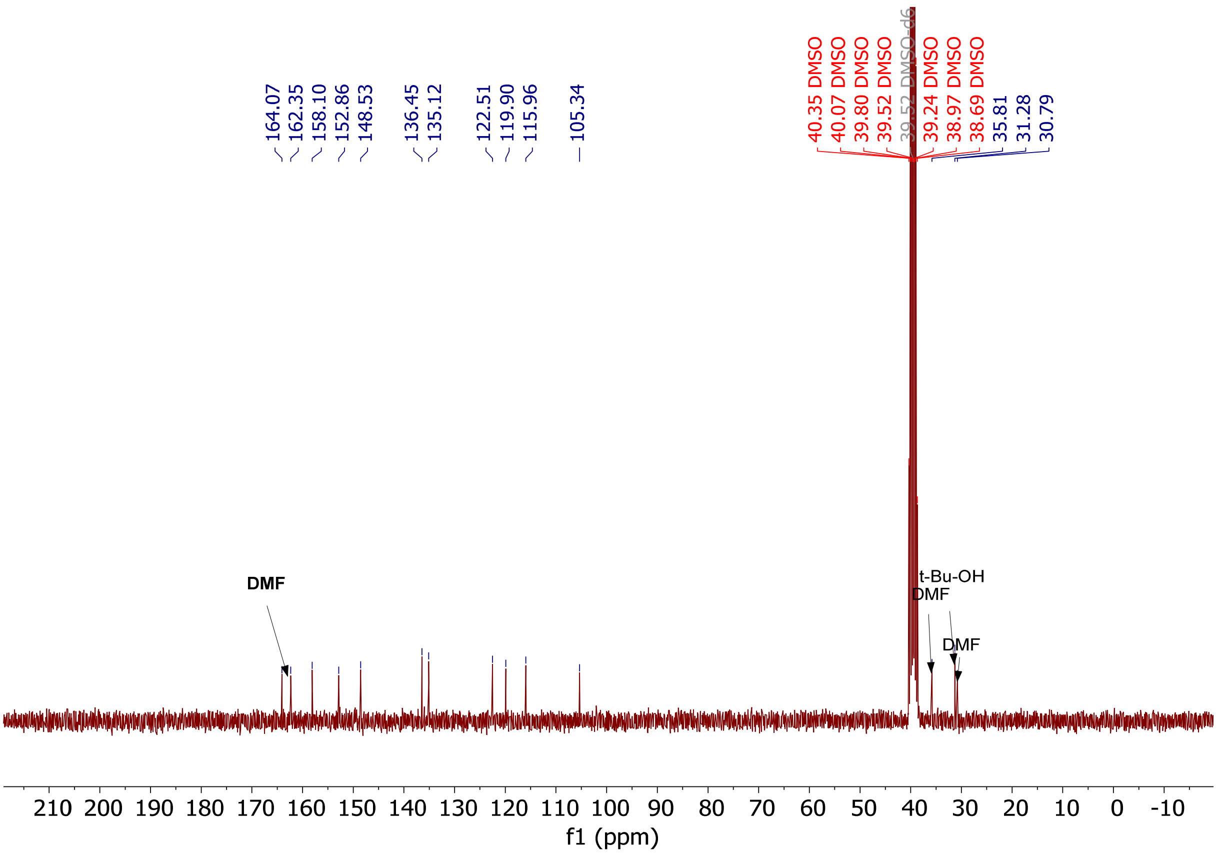

Figure S6. ^{13}C NMR spectrum of bbpya in DMSO-d^{6} at 298 K.

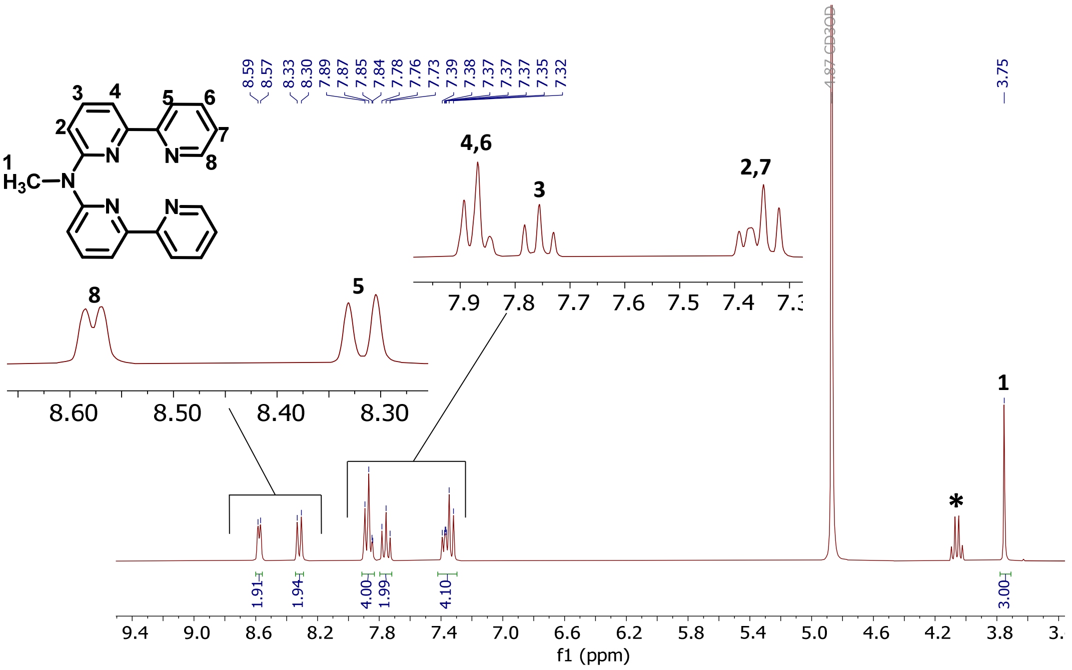

Figure S7. ^{1}H NMR spectrum of Mebbpya in MeOD at 298 K. (*) residual solvent (EtOAc).

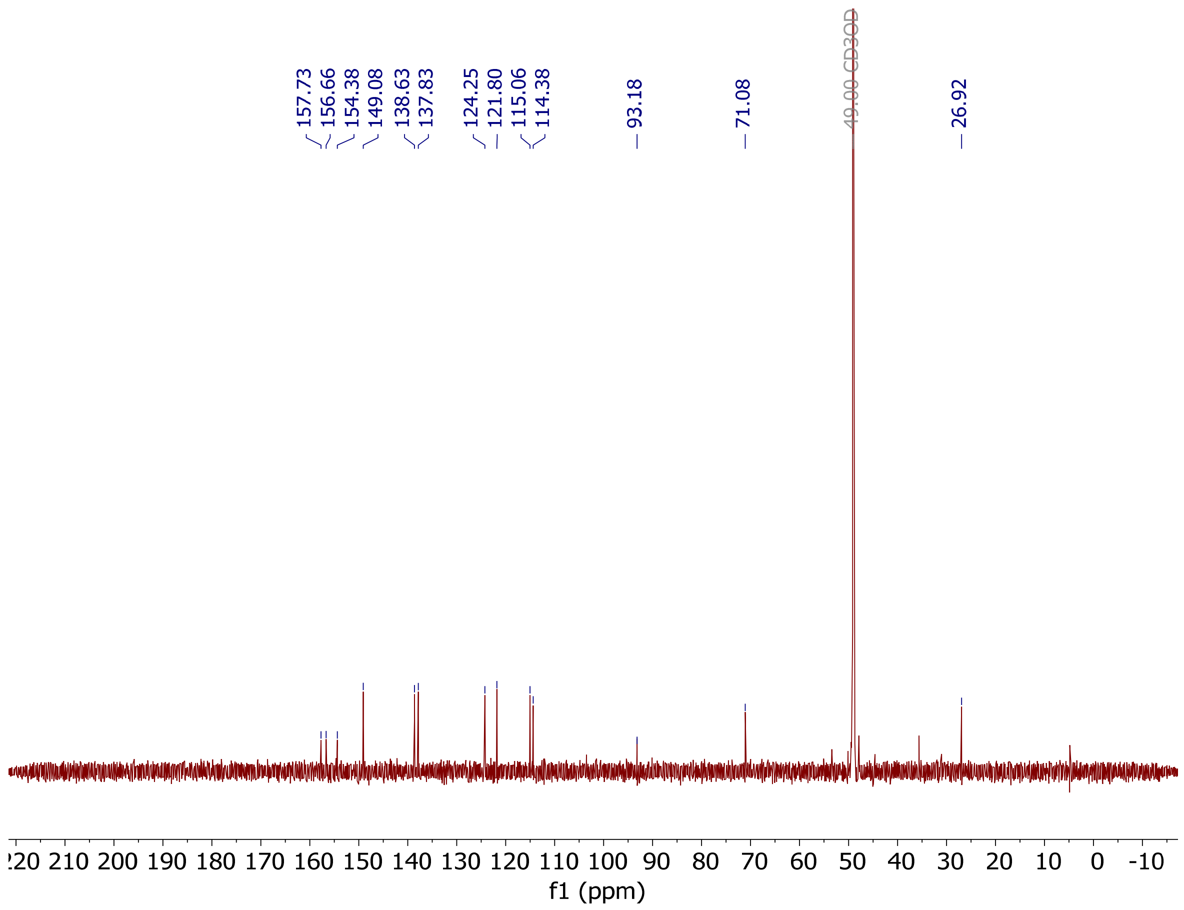

Figure S8. ^{13}C NMR spectrum of Mebbpya ligand in MeOD at 298 K.

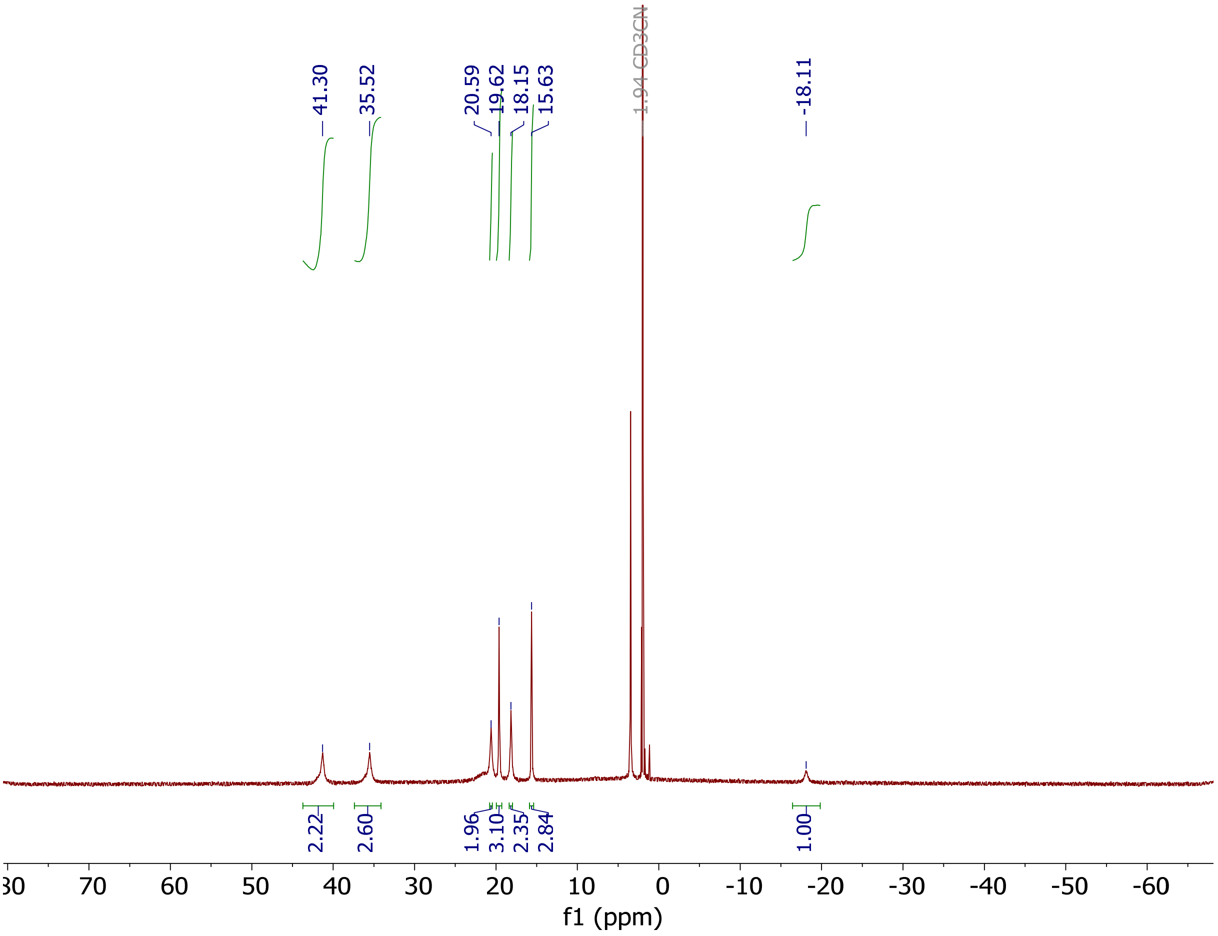

Figure S9. ^{1}H NMR spectrum of the catalyst **[(Hbbpya)Co^{II}]$^{2+}$** in CD_3CN at 298 K.

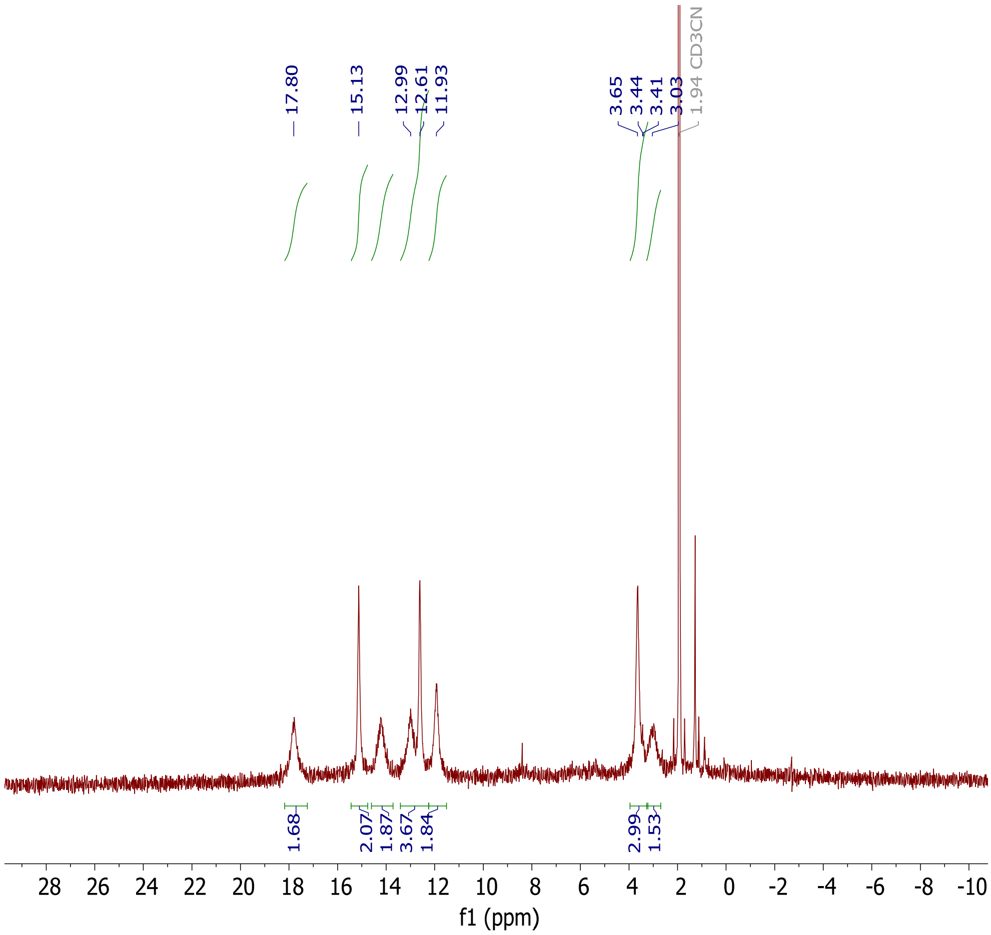

Figure S10. ^{1}H NMR spectrum of the catalyst **[(Mebbpya)Co^{II}]$^{2+}$** in CD_3CN at 298 K.

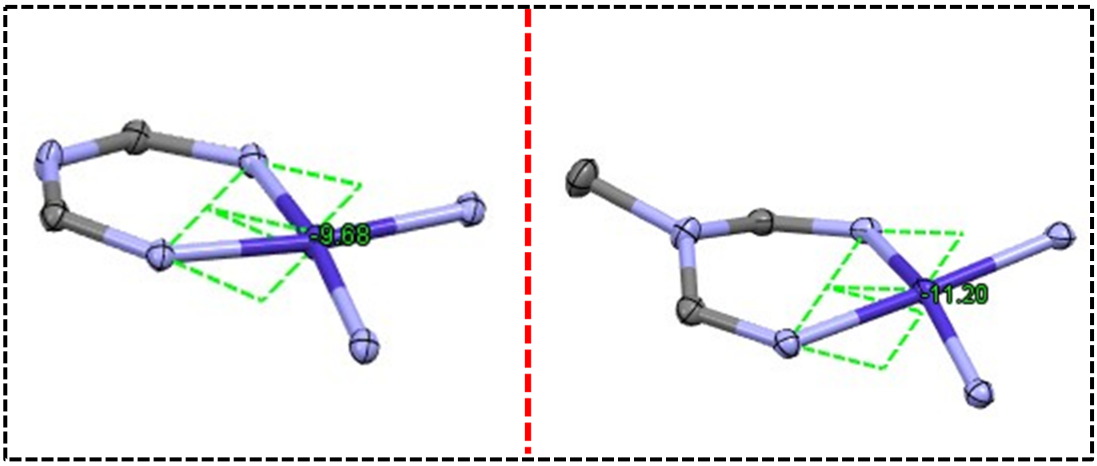

Figure S11. The N-N-N-N dihedral angles of **[(Hbbpya)Co^{II}]$^{2+}$** (left) and **[(Mebbpya)Co^{II}]$^{2+}$** (right) as obtained from X-ray crystallography. Non-essential atoms have been omitted for clarity.

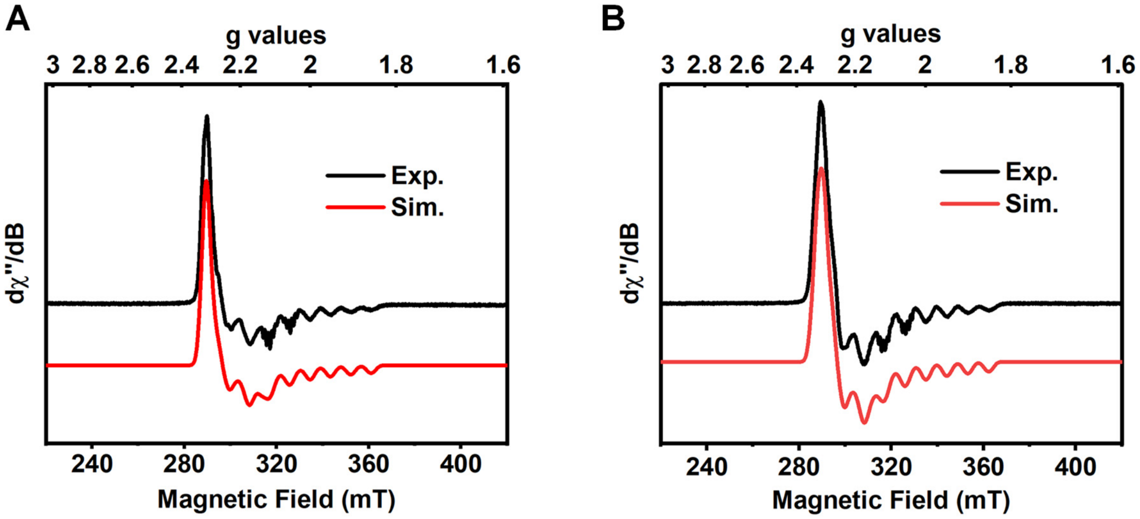

Figure S12. X-band EPR spectra of (A) [**(Hbbpya)Co^{II}**]$^{2+}$, (B) [**(Mebbpya)Co^{II}**]$^{2+}$ in frozen butyronitrile solutions. Conditions: microwave frequency ~9.37 GHz, microwave power 0.016 mW, modulation amplitude 0.5 mT, temperature 14 K. Experimental spectra are shown in black and the simulations in red. The following parameters were used for the simulations: (A) $g_x = 2.254$, $g_y = 2.220$, $g_z = 2.026$, lwpp = [2.65 0.95], $|A_{Co}|$, / MHz = 27, 91, 249; (B) $g_x = 2.271$, $g_y = 2.215$, $g_z = 2.025$, lwpp = 2.55, $|A_{Co}|$, / MHz = 36, 63, 258.

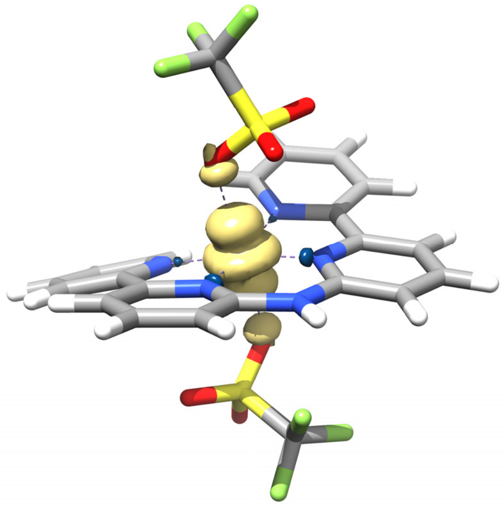

Figure S13. Spin density plots at an isovalue of 0.003 as obtained from DFT at the BP86 level for **[(Hbbpya)Co^{II}]$^{2+}$**.

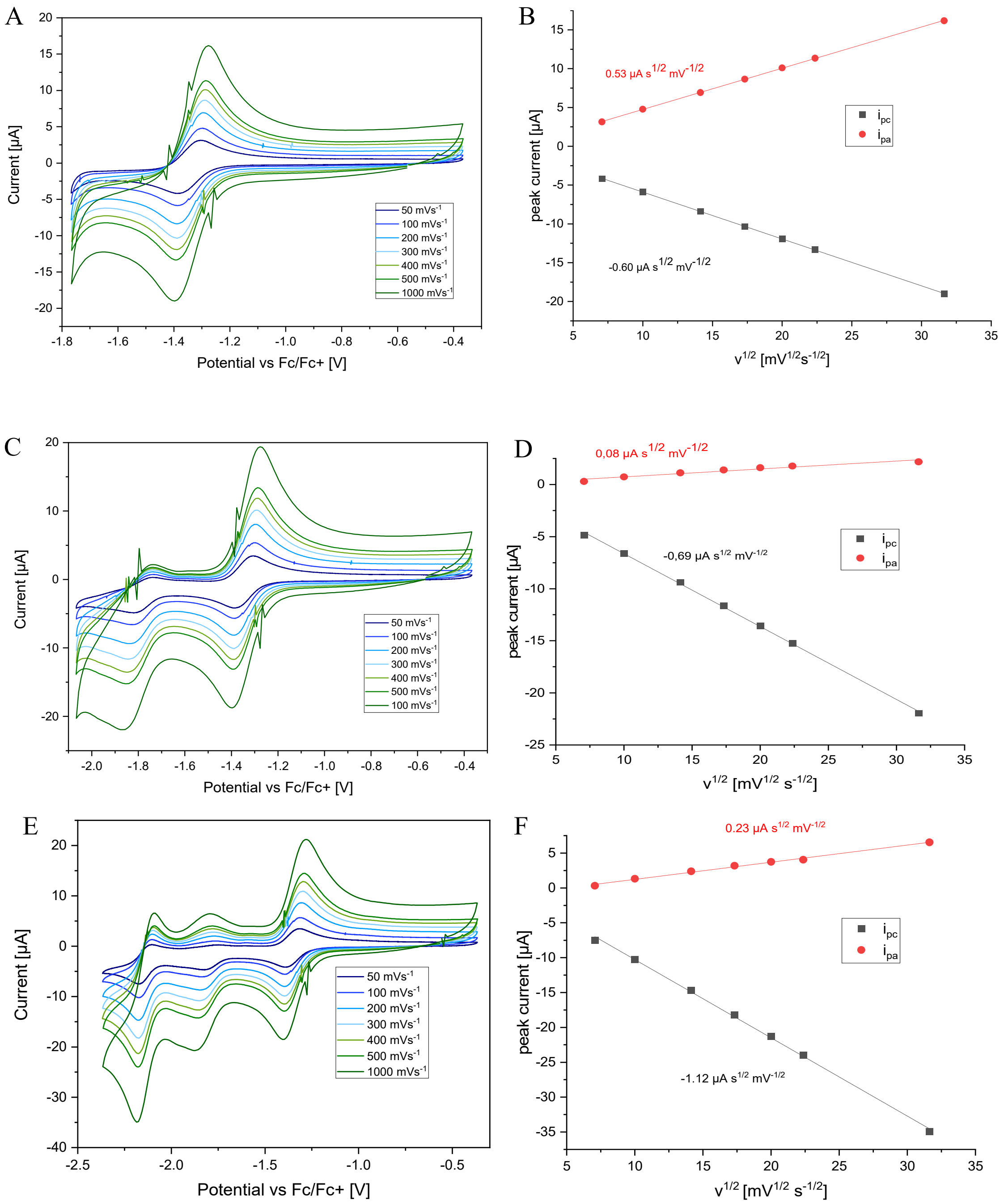

Figure S14. CVs of 0.5 mM **[(Hbbpya)Co^{II}]$^{2+}$** in MeCN with 0.1 M [$^{n}Bu_4N$]PF_6 at different potential ranges at different scan rates (A, C, and E). Scan rate dependence of the peak currents are shown in B, D, and F.

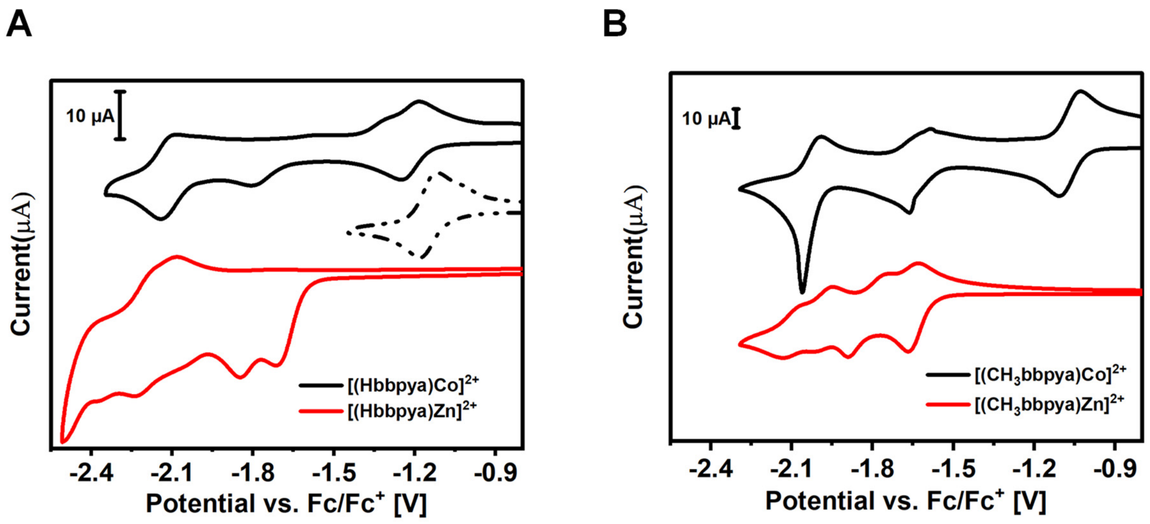

Figure S15. Comparison of CV of 1 mM (A) **[(Hbbpya)Co^{II}]$^{2+}$** (black), **[(Hbbpya)Zn^{II}]$^{2+}$** (red), (B) **[(Mebbpya)Co^{II}]$^{2+}$** (black), and **[(Mebbpya)Zn^{II}]$^{2+}$** (red) in MeCN at a scan rate of 100 mV s^{-1}.

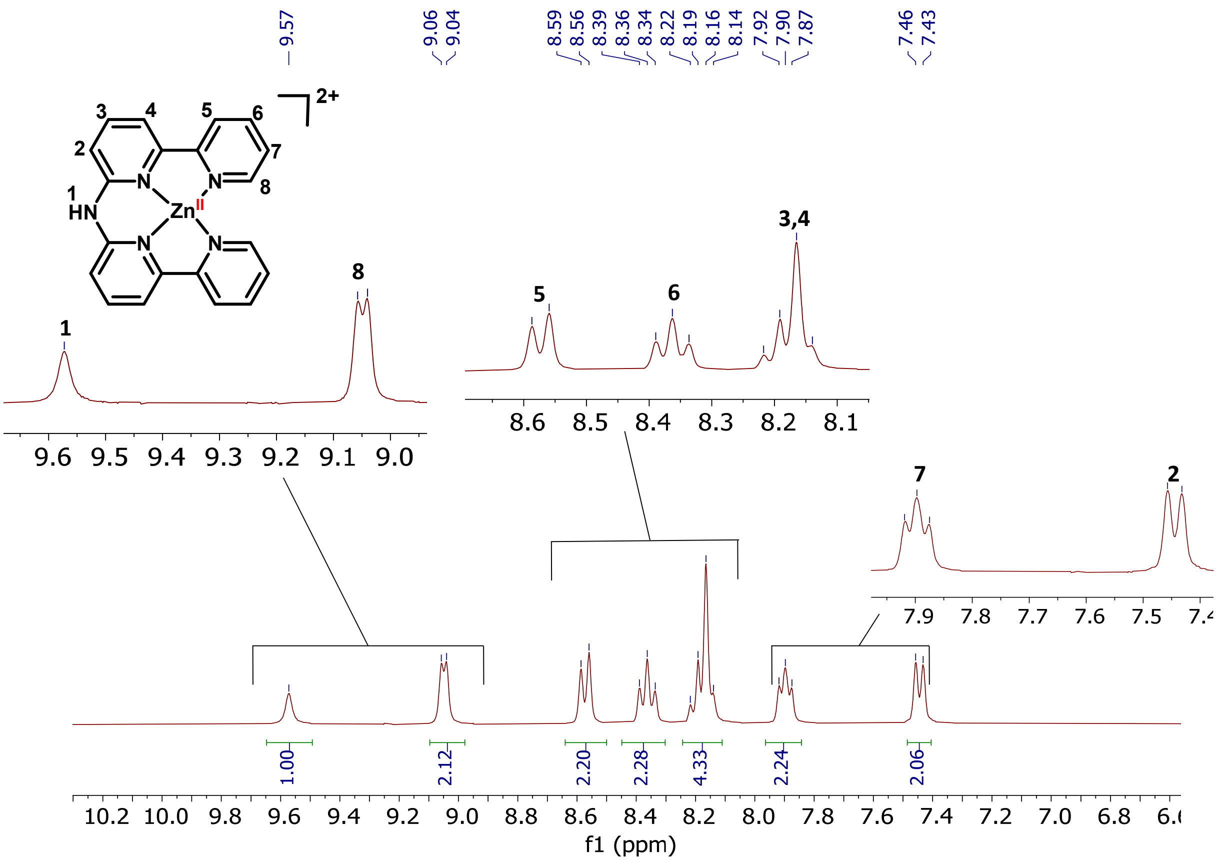

Figure S16. ^{1}H NMR spectrum of **[(Hbbpya)Zn^{II}]$^{2+}$** in CD_3CN at 298 K.

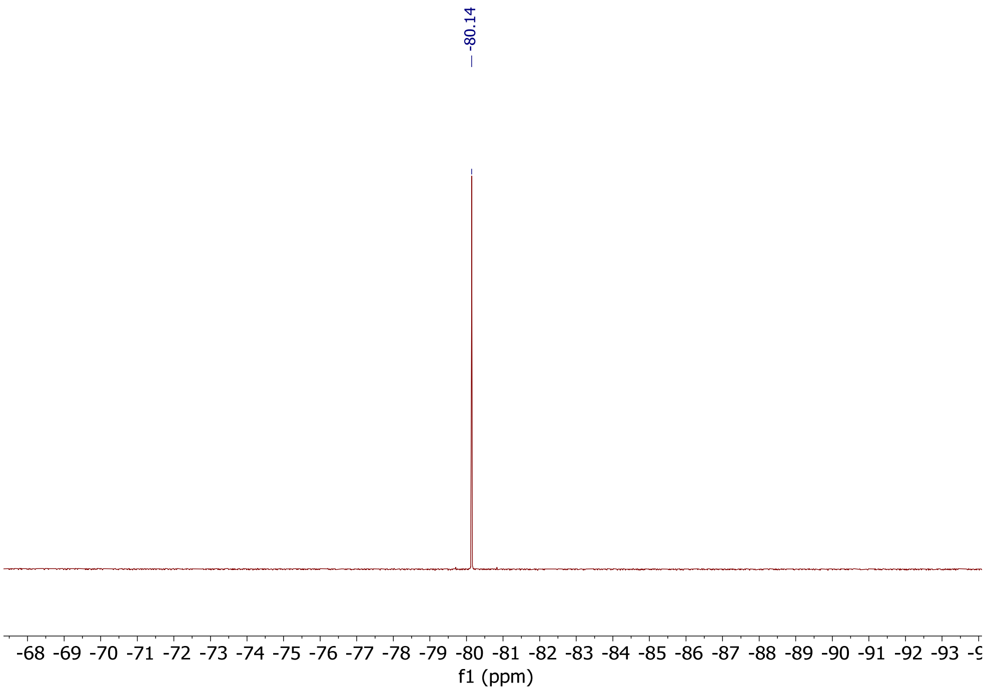

Figure S17. ^{19}F NMR spectrum of **[(Hbbpya)Zn^{II}]$^{2+}$** in CD_3CN at 298 K.

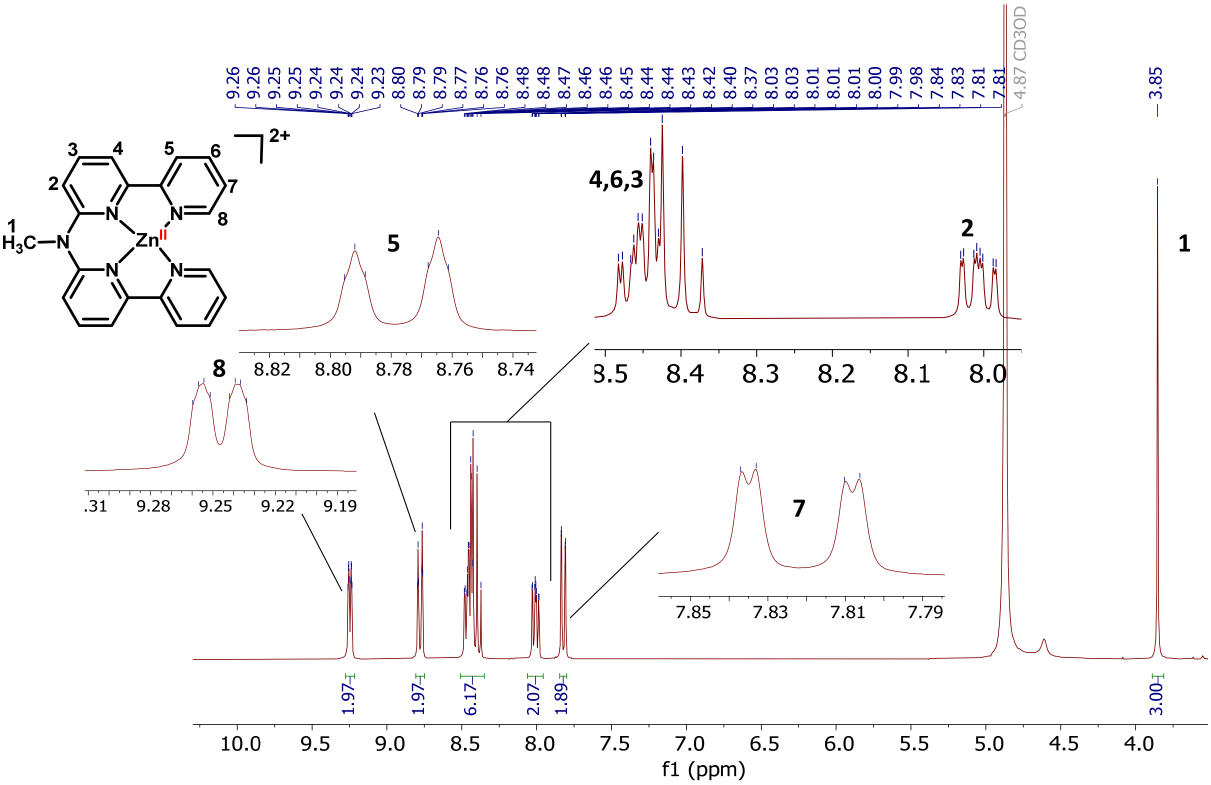

Figure S18. ^{1}H NMR spectrum of **[(Mebbpya)Zn^{II}]$^{2+}$** in MeOD at 298 K.

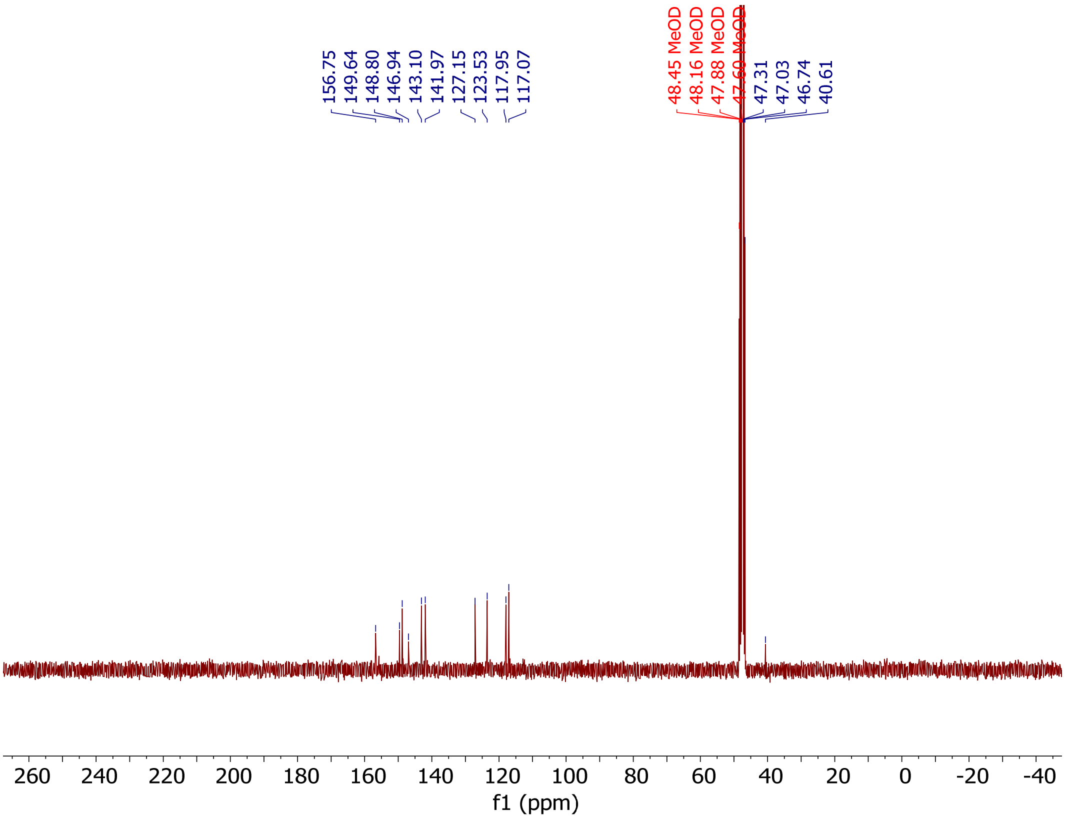

Figure S19. ^{13}C NMR spectrum of **[(Mebbpya)Zn^{II}]$^{2+}$** in MeOD at 298 K.

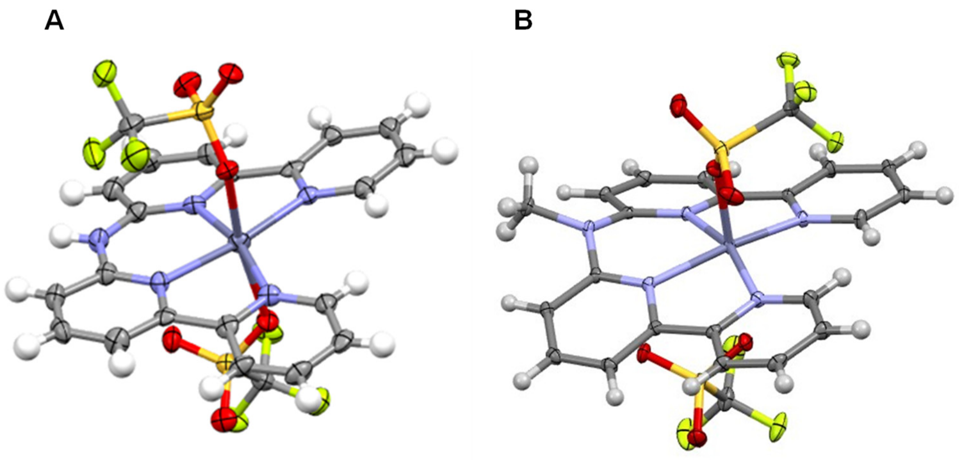

Figure S20. SC-XRD of (A) **[(Hbbpya)Zn^{II}]$^{2+}$**, and (B) **[(Mebbpya)Zn^{II}]$^{2+}$**. Thermal ellipsoids are drawn at the 50% probability level.

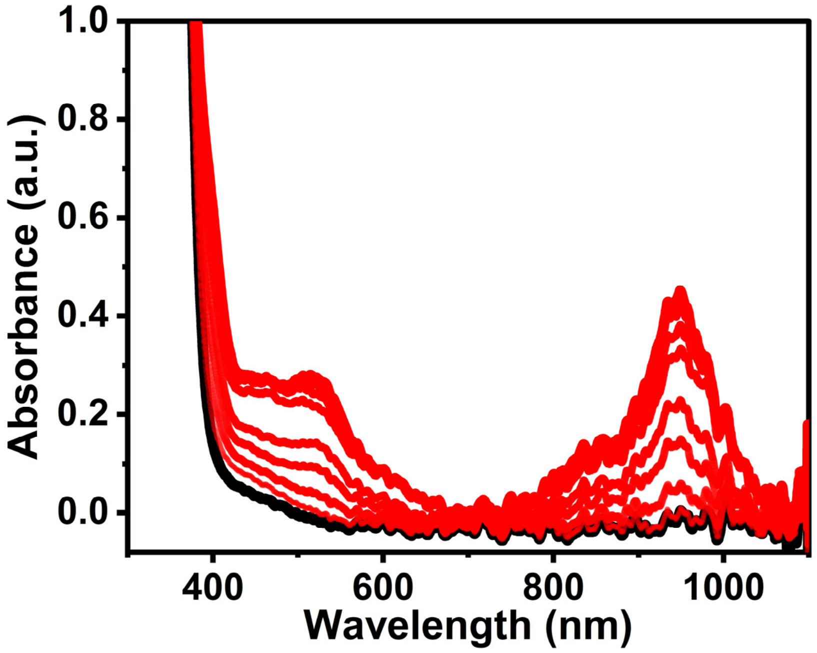

Figure S21. Spectro-electrochemical UV-Vis of **[(Hbbpya)Co^{II}]$^{2+}$** in MeCN up to first reduction potential (up to a scan of -1.36 V vs. Fc$^{+/0}$) showing the gradual generation of **[(Hbbpya)Co^{I}]$^{+}$** (red) from **[(Hbbpya)Co^{II}]$^{2+}$** (black).

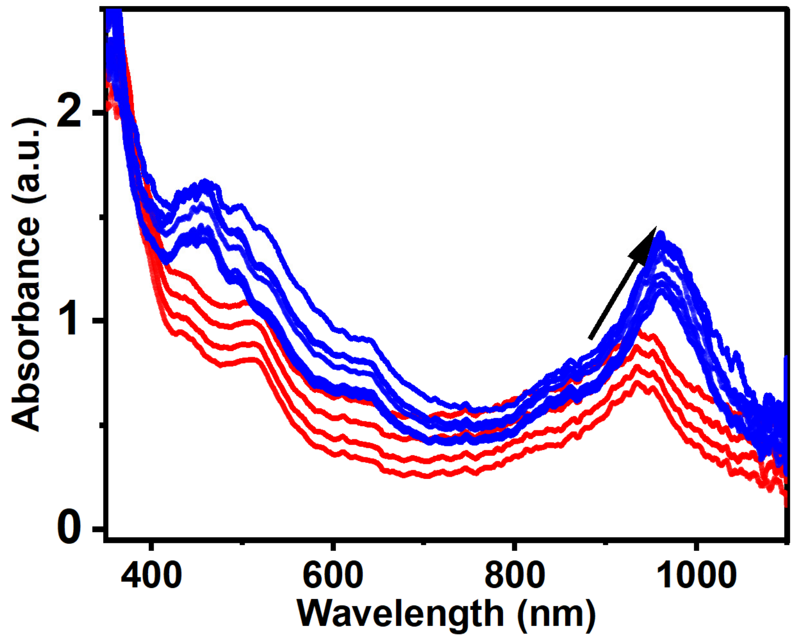

Figure S22. Spectro-electrochemical UV-Vis of **[(Hbbpya)Co^{II}]$^{2+}$** in MeCN scanning the potential from -1.35 V to -1.8 V vs. Fc$^{+/0}$ showing the gradual generation of the mixture of **[(Hbbpya^{-})Co^{I}]^{0}** and **[(bbpya^{-})Co^{I}]**^{0} (blue) from **[(Hbbpya)Co^{I}]**$^{+}$ (red).

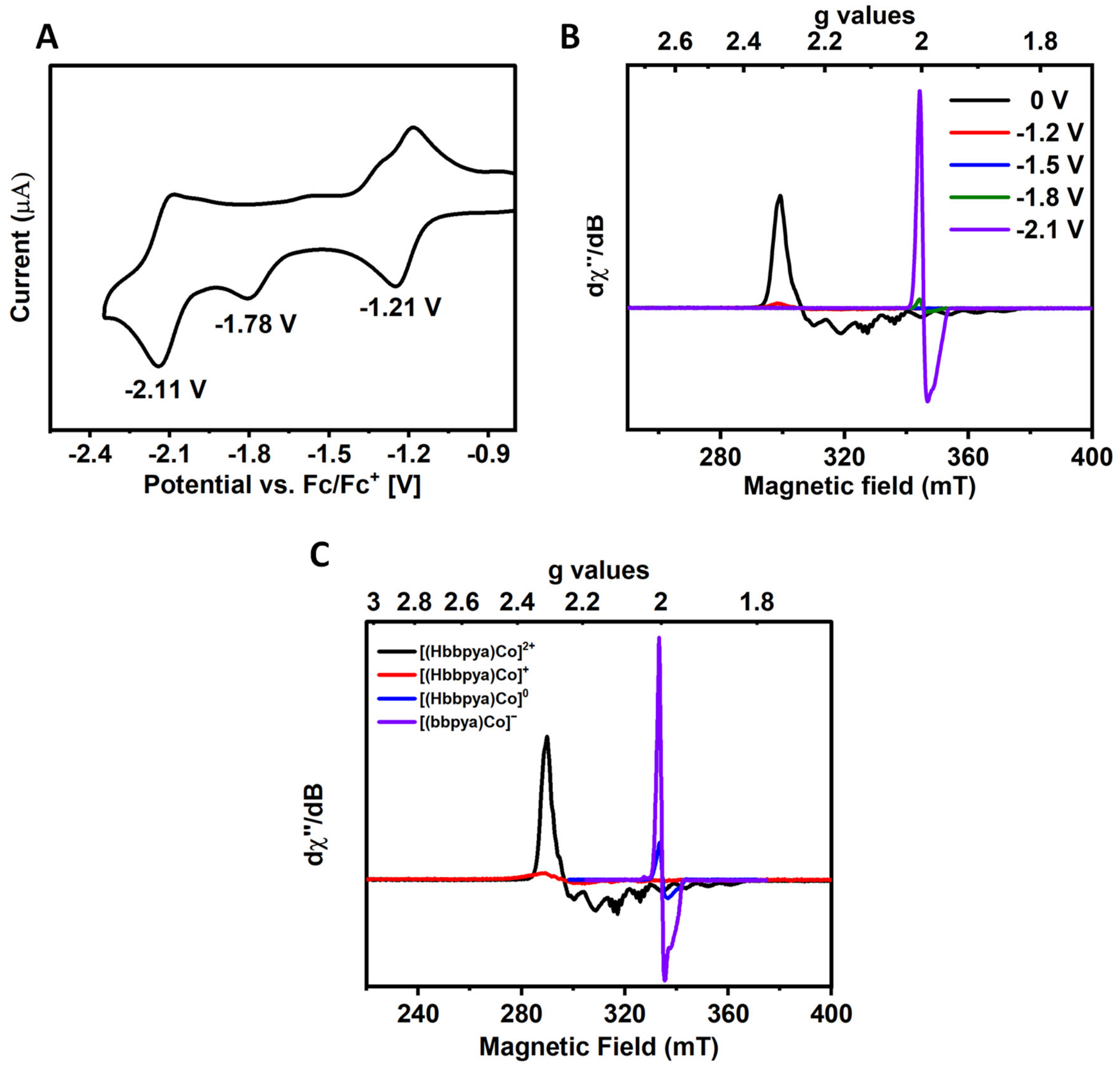

Figure S23. (A) CV, and (B) Spectro-electrochemical EPR (at various potentials) of **[(Hbbpya)Co^{II}]$^{2+}$** in MeCN with 0.1 M TBAPF$_6$. Experimental conditions: temperature 30 K, microwave frequency 9.65 GHz, microwave power 0.16 mW, modulation amplitude 0.7 mT. (C) Overlay of the X-band EPR spectra of the chemically generated reduced species of **[(Hbbpya)Co^{II}]$^{2+}$**.

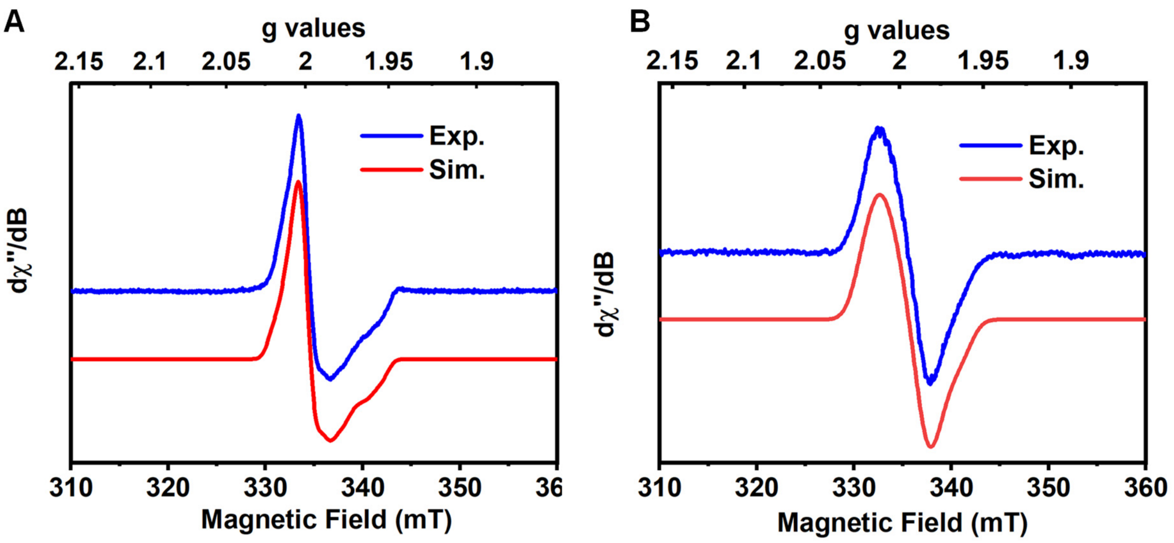

Figure S24. X-band EPR spectra of (A) **[(Hbbpya⁻)Co^{I}]^{0}** (experimental: blue and simulation: red), (B) **[(Mebbpya⁻)Co^{I}]^{0}** (experimental: blue and simulation: red) respectively. The following parameters were used for the simulations: (A) g_x = 2.007, g_y = 2.003, g_z = 1.971, lwpp = [1.97 0.96], $|A_N|$, / MHz = 0, 42, 18; $|A_{N'}|$, / MHz = 0, 28, 43. (B) g_x = 2.014, g_y = 1.988, g_z = 1.978, lwpp = [2.44 0.42], anisotropic broadenings (in MHz): $H_{Strain}(1)$ = 0, $H_{Strain}(2)$ = 38, $H_{strain}(3)$ = 134.

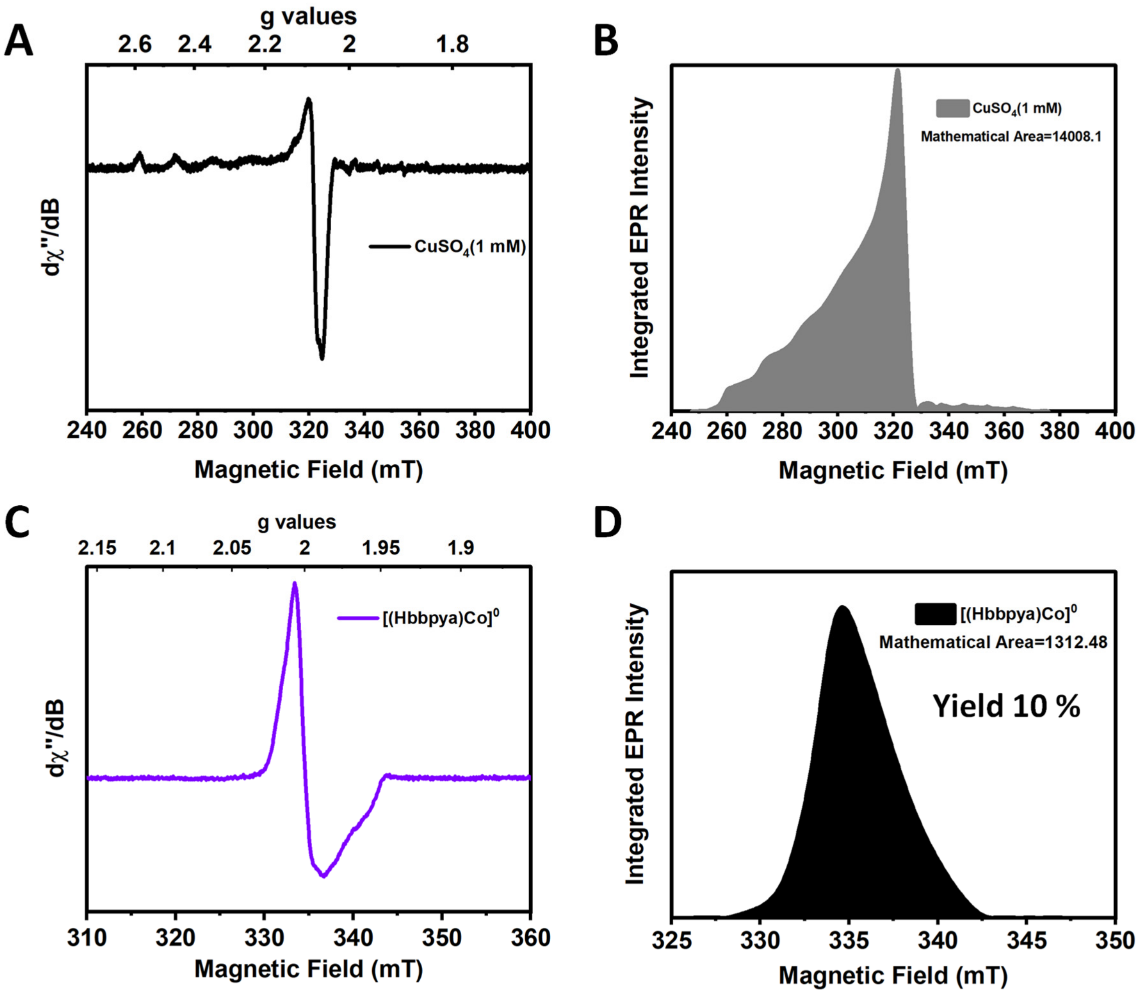

Figure S25. (A) X-band EPR analysis of 1 mM solution of $CuSO_4$. Experimental conditions: microwave frequency 9.35 GHz, microwave power 0.00025 mW, modulation amplitude 0.4 mT, temperature 13 K. (B) Integration of the signal in (A). (C) X-band EPR spectra of **[(Hbbpya$^{\cdot-}$)Co^{I}]^{0}** measured at the same experimental condition as that of $CuSO_4$. (D) Integral of the signal in (C). By comparison of the area to that calculated from $CuSO_4$ (B), it allows spin quantification of **[(Hbbpya$^{\cdot-}$)Co^{I}]^{0}**, which is thus obtained in ca. 10% yield.

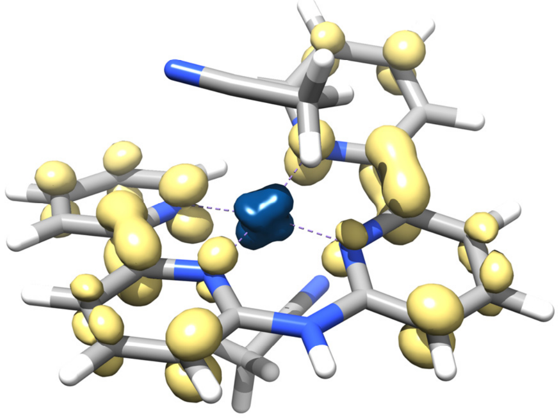

Figure S26. [(Hbbpya$^{\cdot-}$)Co^{I}]^{0} where a low-spin Co(I) center is ligated to a single unpaired electron delocalized over the entire ligand as obtained from DFT at the BP86 level.

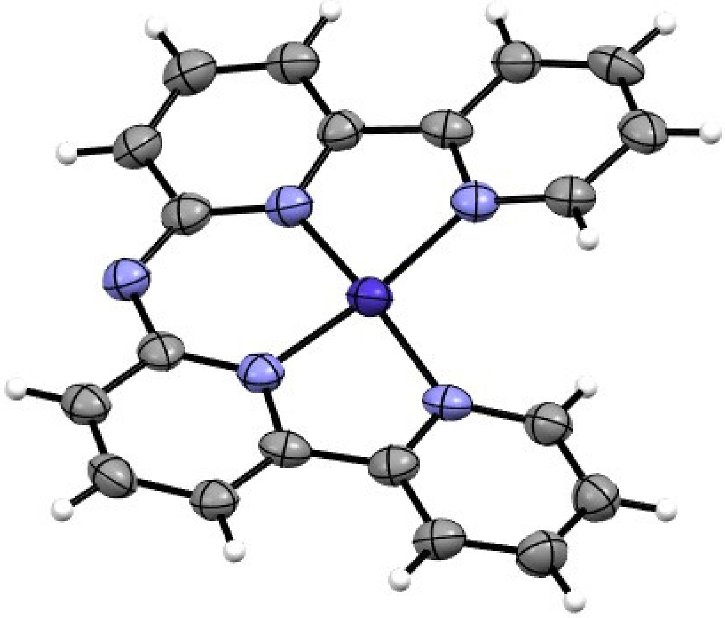

Figure S27. SC-XRD of **[(bbpya^{-})Co^{I}]^{0}**, formed via hydrogen atom loss of **[(Hbbpya$^{\cdot-}$)Co^{I}]^{0}** in reaction of two equiv. of $CoCp_2^*$ and **[(Hbbpya)Co^{II}]$^{2+}$** resulting in H_2 release (see Scheme S4). Thermal ellipsoids are drawn at the 50% probability level.

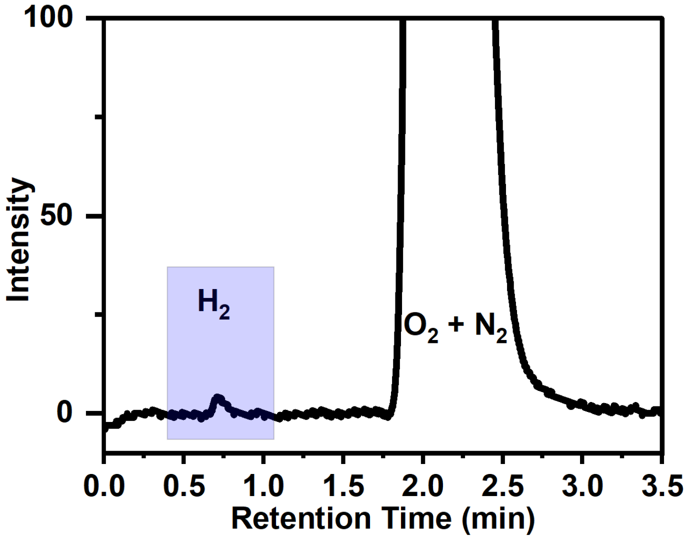

Figure S28. Detection of H_2 gas by GC during the transformation of **[(Hbbpya$^-$)Co^I]^0** to **[(bbpya$^-$)Co^I]^0** in MeCN.

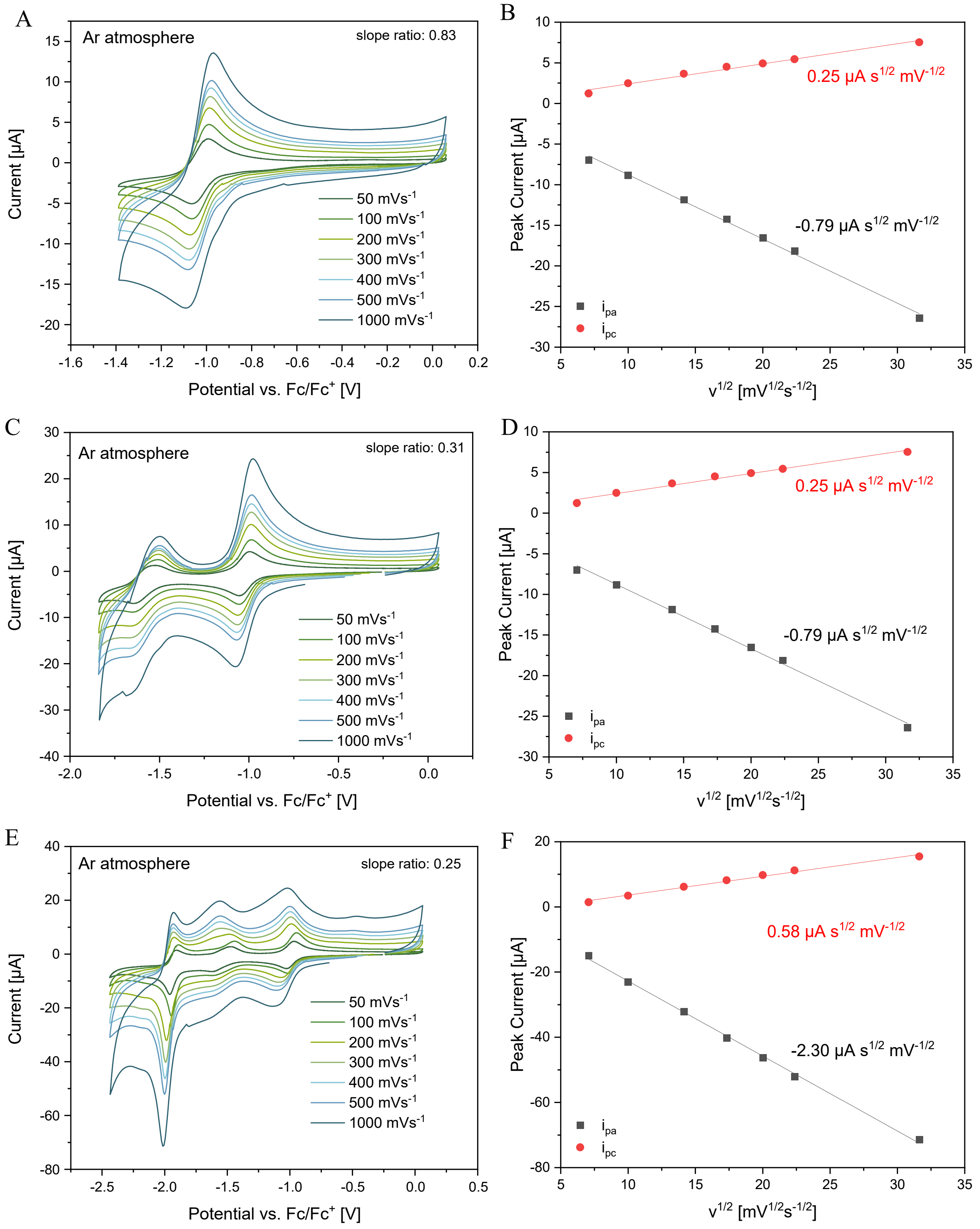

Figure S29. CVs of 0.5 mM **[(Mebbpya)Co^{II}]$^{2+}$** in MeCN with 0.1 M [^{n}Bu$_4$N]PF$_6$ at different potential ranges at different scan rates (A, C, and E). Scan rate dependence of the peak currents (B, D, and F).

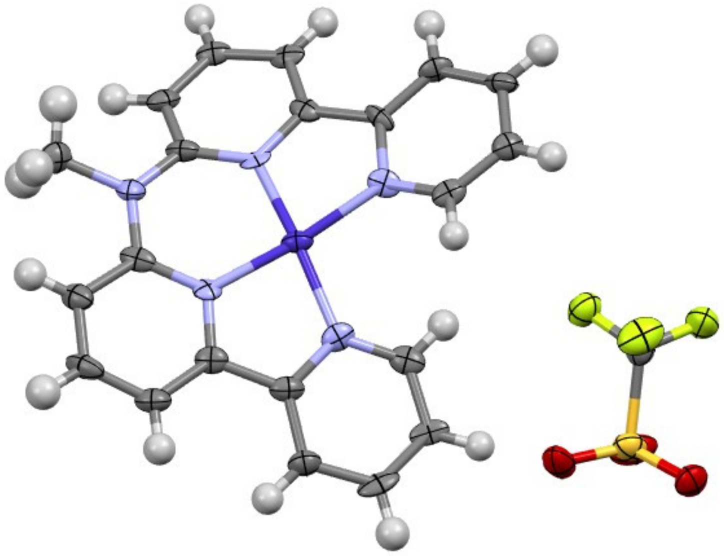

Figure S30. SC-XRD of **[(Mebbpya)Co^{I}]**$^{+}$, generated by the reaction of **[(Mebbpya)Co^{II}]$^{2+}$** with one equiv. of KC_8. Thermal ellipsoids are drawn at the 50% probability level.

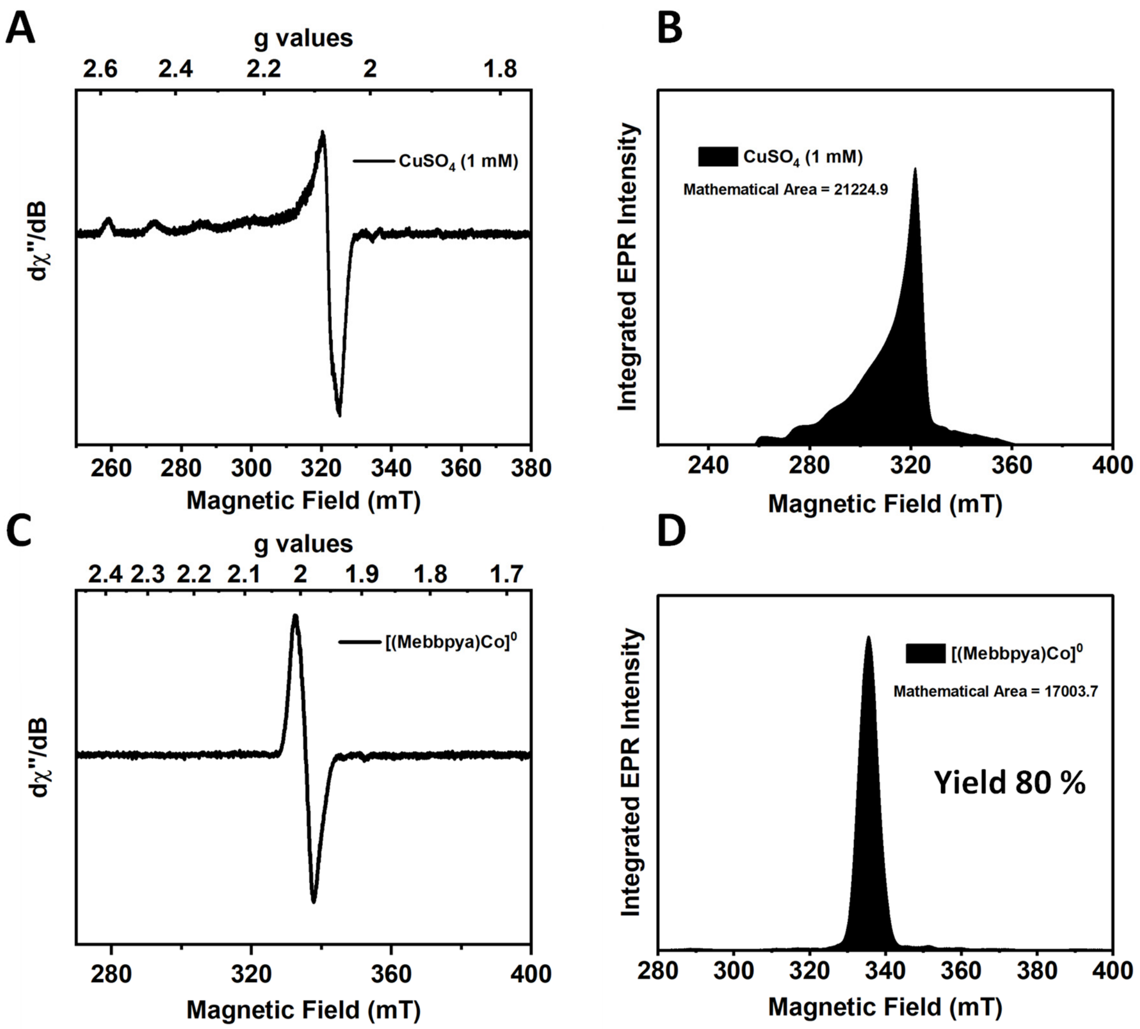

Figure S31. (A) X-band EPR analysis of 1 mM solution of $CuSO_4$. Experimental conditions: microwave frequency 9.35 GHz, microwave power 0.016 mW, modulation amplitude 0.1 mT, temperature 13 K. (B) Integration of the signal in (A). (C) X-band EPR spectra of **[(Mebbpya$^{\bullet-}$)Co^{I}]^{0}** measured at the same experimental condition as that of $CuSO_4$. (D) Integral of the signal in (C). By comparison of the area to that calculated from $CuSO_4$ (B), it allows spin quantification of **[(Mebbpya $^{\bullet-}$)Co^{I}]^{0}**, which is thus obtained in ca. 80% yield.

KIE-Determination for [(Hbbpya)Co]$^{2+}$ and [(Mebbpya)Co]$^{2+}$:

Scan rate-dependent experiments were carried out and in both cases no s-shape curves were observed at 100 mV s^{-1}, consequently the system is not in the pure kinetic region. In order to bring the system as close as possible to the pure kinetic region, the scan rate was significantly reduced (3 to 5 mV s^{-1}), and the systems showed a higher s-shape character, although the pure kinetic region was not fully reached.

The kinetic isotope effect (KIE) investigations were conducted in the intermediate kinetic region rather than the pure kinetic regime, resulting in deviations from the ideal system. To minimize these deviations, the systems could be adjusted to approach the pure kinetic region as closely as possible.

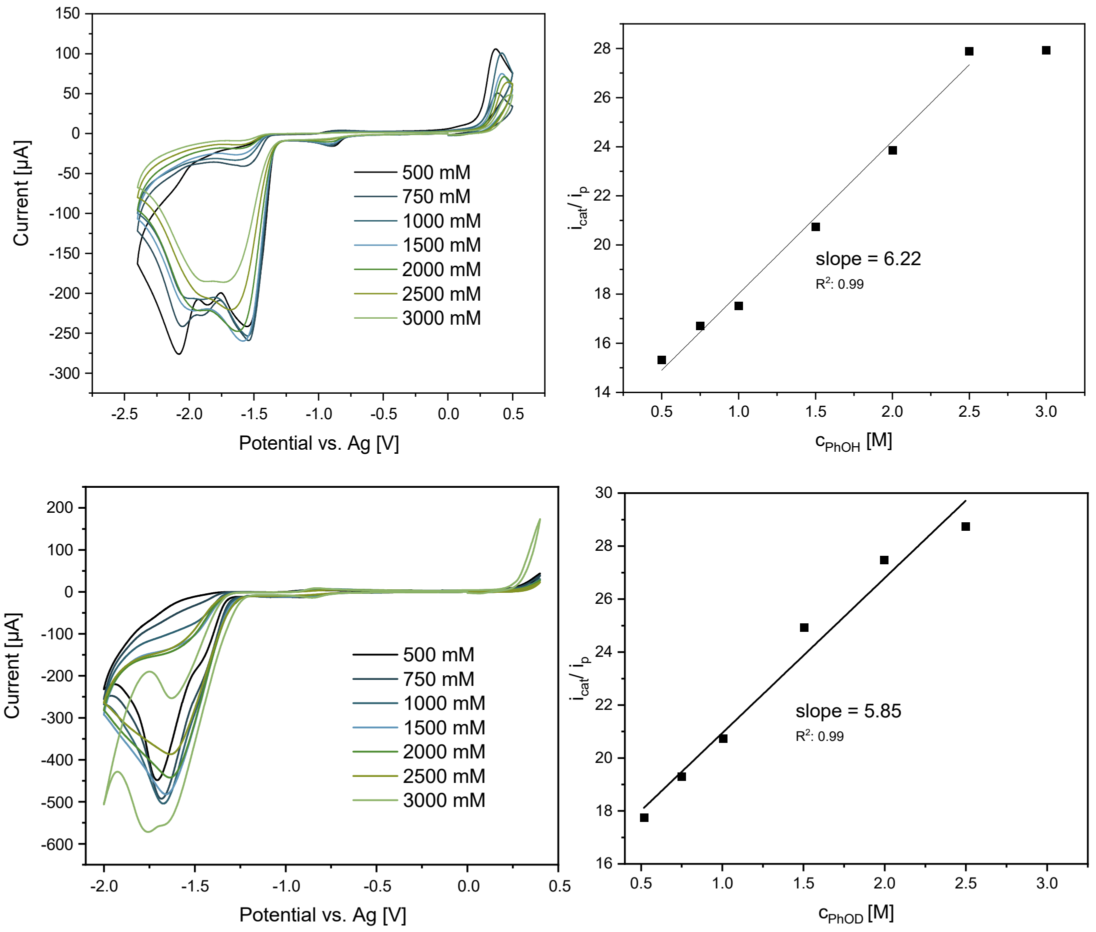

Figure S32. CVs of 0.5 mM **[(Hbbpya)Co^{II}]$^{2+}$** in MeCN with varying amounts of PhOH (top) or PhOD (bottom) purged with CO_2 with 0.1 M [^{n}Bu$_4$N]PF$_6$ with a scan rate of 100 mV s^{-1}. KIE = 1.11.

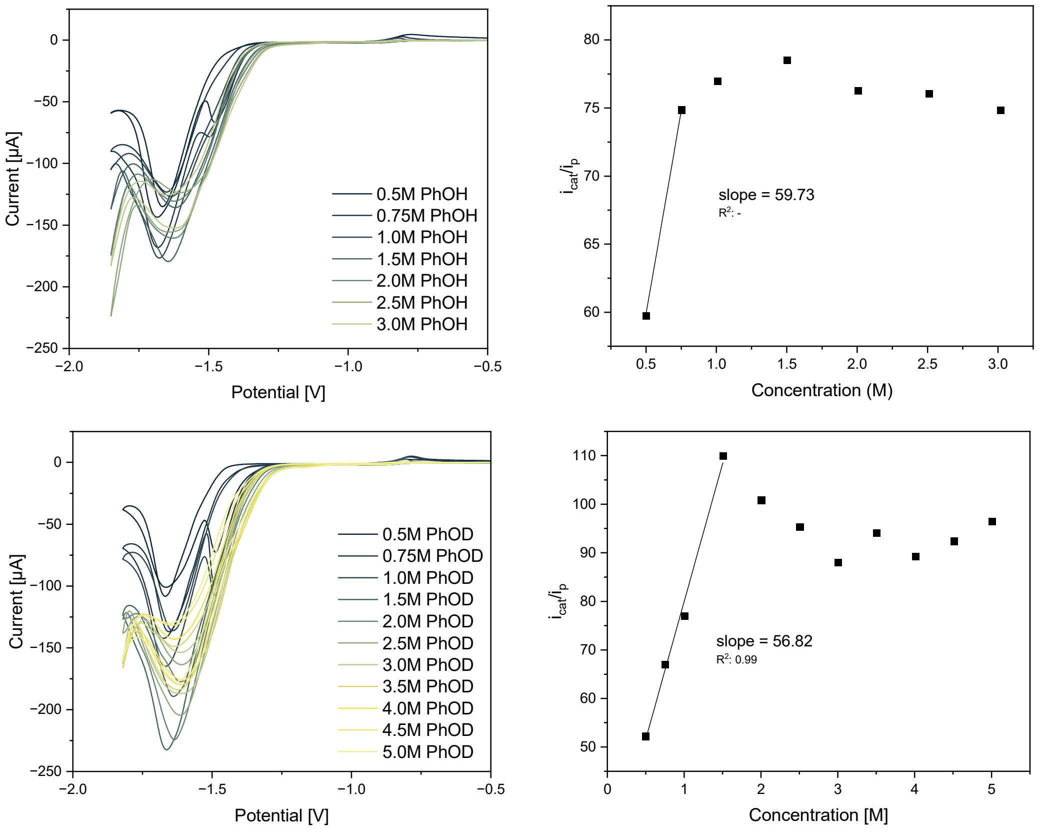

Figure S33. CVs of 0.5 mM **[(Hbbpya)Co^{II}]$^{2+}$** in MeCN with varying amounts of PhOH (top) or PhOD (bottom) purged with CO_2 with 0.1 M [^{n}Bu$_4$N]PF$_6$ for the determination of the KIE values with a scan rate of 3 mV s^{-1}.

So, KIE for [(Hbbpya)Co]$^{2+}$ $= \frac{k_{PhOH}}{k_{PhOD}} = (\frac{slope_{PhOH}}{slope_{PhOD}})^2 = (\frac{59.73}{56.82})^2 = 1.11$

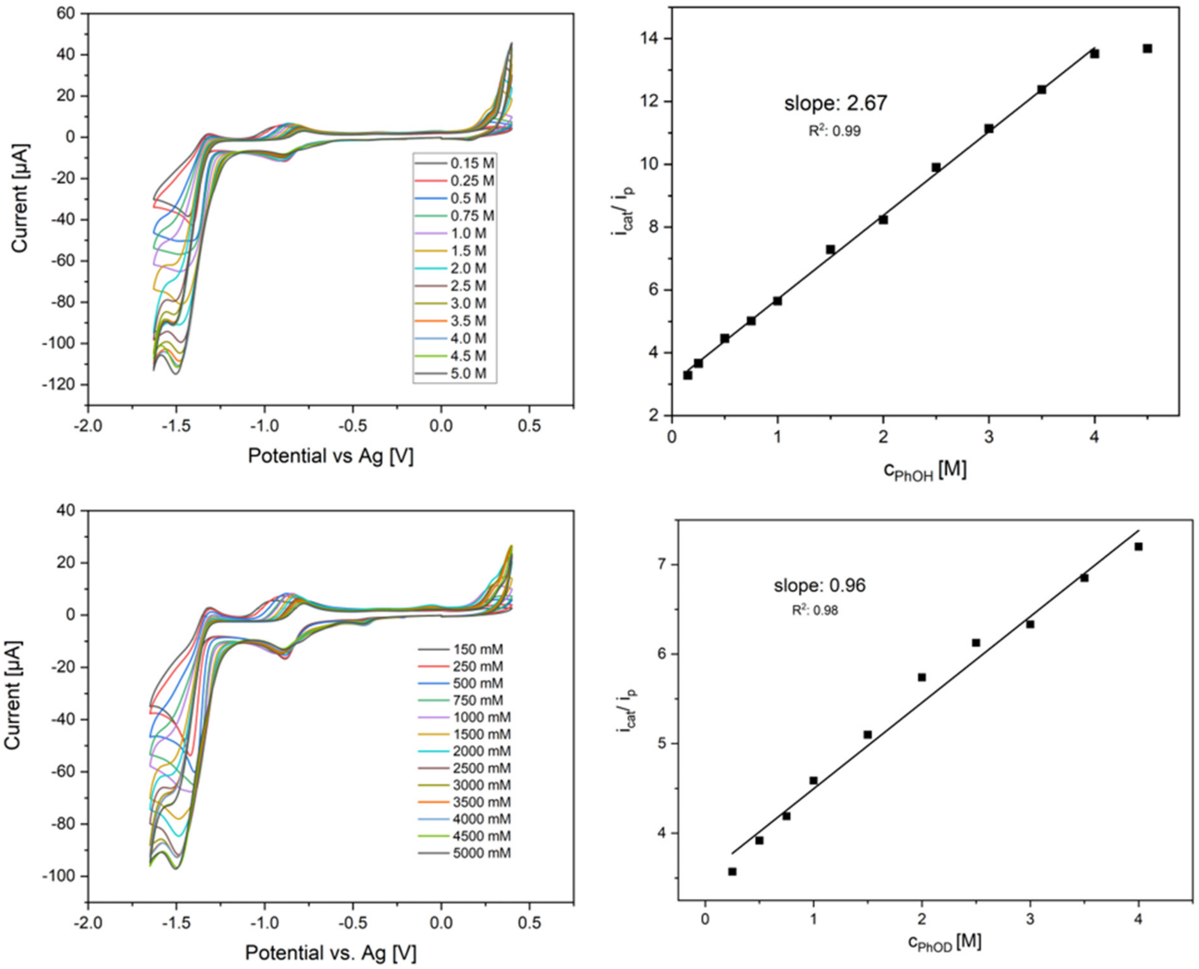

Figure S34. CVs of 0.5 mM **[(Mebbpya)Co^{II}]** $^{2+}$ in MeCN with varying amounts of PhOH (top) or PhOD (bottom) purged with CO_2 with 0.1 M [$^{n}Bu_4N$]PF_6 with a scan rate of 100 mV s^{-1}. KIE = 7.73.

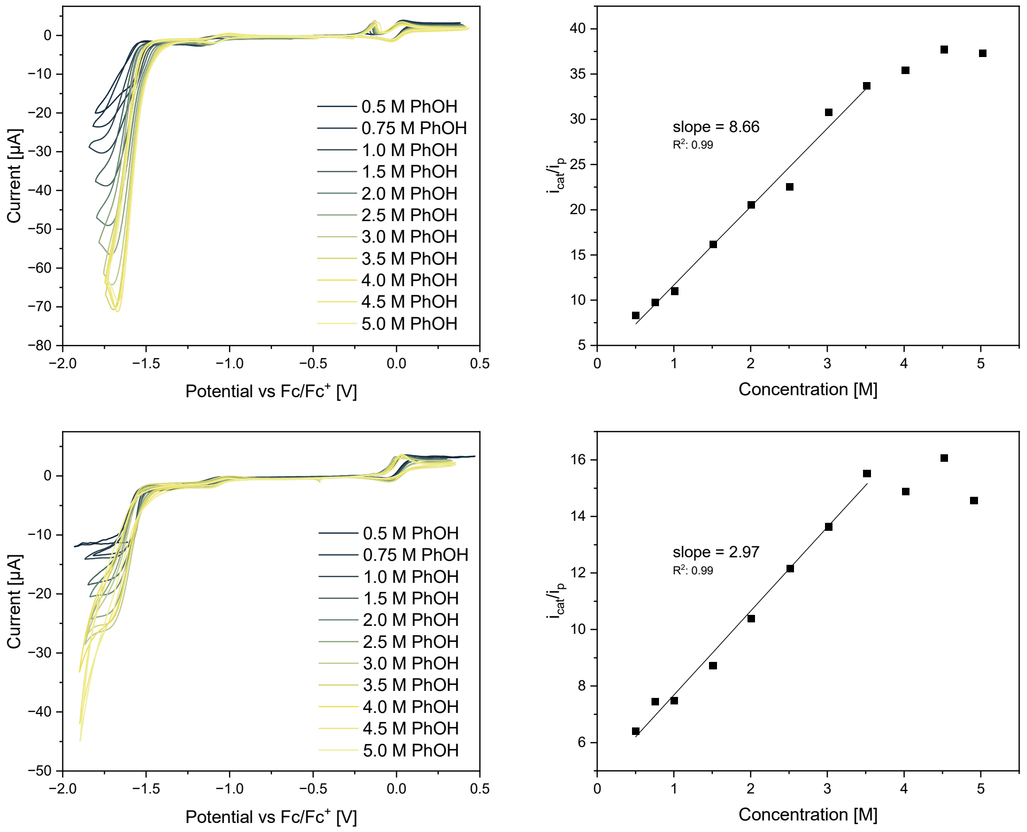

Figure S35. CVs of 0.5 mM **[(Mebbpya)Co^{II}]** $^{2+}$ in MeCN with varying amounts of PhOH (top) or PhOD (bottom) purged with CO_2 with 0.1 M [^{n}Bu$_4$N]PF_6 for the determination of the KIE values with a scan rate of 5 mV s^{-1} and 3 mV s^{-1}, respectively.

So, KIE for [(Hbbpya)Co]$^{2+}$ = $\frac{k_{PhOH}}{k_{PhOD}} = (\frac{slope_{PhOH}}{slope_{PhOD}})^2 = (\frac{8.66}{2.97})^2 = 8.50$

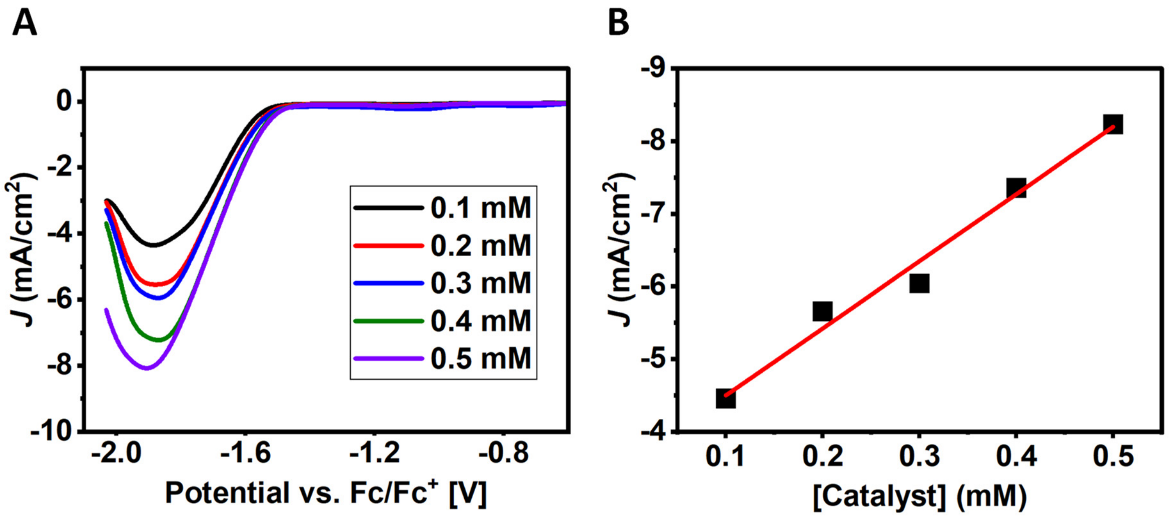

Figure S36. (A) Current response under CO_2-saturation conditions with 3 M PhOH for varying catalyst concentration (mM) of **[(Hbbpya)Co]$^{2+}$**. (B) Plot of peak current density vs concentration of **[(Hbbpya)Co]$^{2+}$**. Scan rate 100 mV s^{-1}; 0.1 M $TBAPF_6$/MeCN.

Homogeneity tests for the Catalysts under Bulk Electrolysis condition

In order to assess the stability of the catalysts, the electrode surface was examined after the respective 8h electrolysis. As XPS analysis of the electrode surface is not feasible with our equipment, we wanted to scrape off any deposited material and measure it using SEM/EDX. However, no significant depositions were observed for any of the three different complexes, indicating electrocatalytic stability.

To further analyze the electrocatalytic stability of the complexes, the electrolyte solutions were analyzed by UV/vis spectroscopy. For this purpose, a diluted solution of the 0.5 mM complex-containing electrolyte (150 µL 0.5 mM Co(II)-containing electrolyte + 850 µL electrolyte) was measured before electrolysis and then the solution was measured after electrolysis in the same volumetric dilution in order to determine the Co(II) concentration of the solution after electrolysis using the band at 345 nm. The measurements show that the complexes are largely retained, so that electrochemical deposition processes are subordinate.

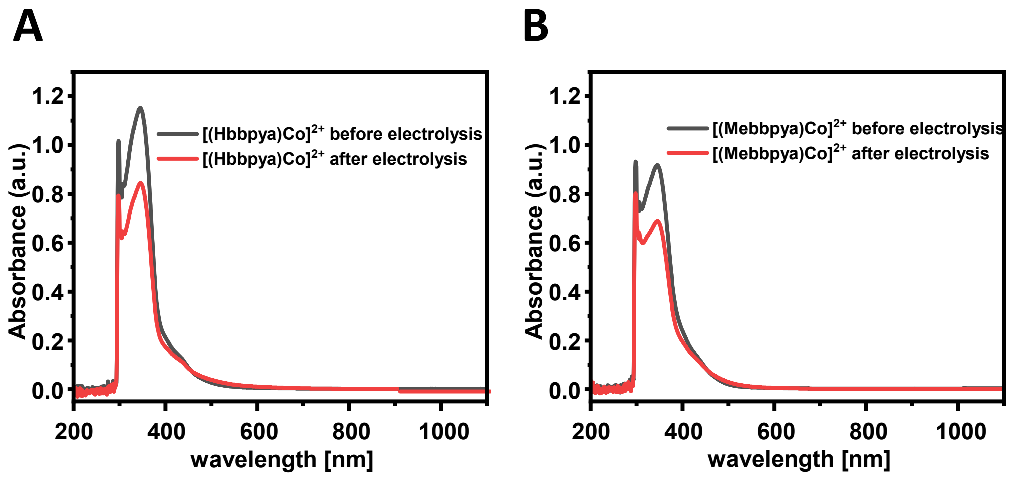

	before electrolysis	after electrolysis	% remaining
**[(Hbbpya)Co]$^{2+}$**	0.5 mM	0.37 mM	74
**[(Mebbpya)Co]$^{2+}$**	0.5 mM	0.38 mM	76

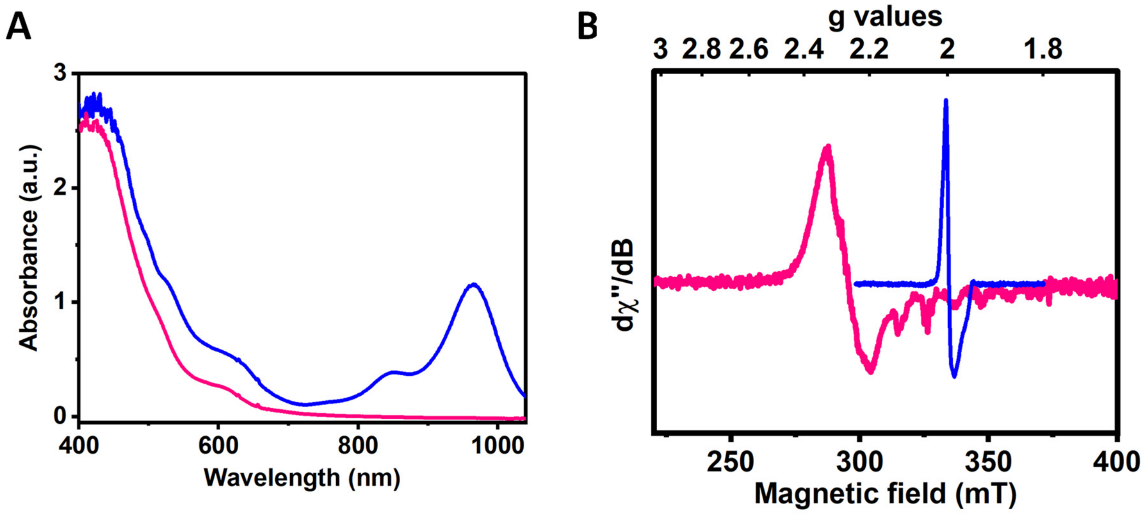

Figure S37. Reaction of **$[(Hbbpya^{-\bullet})Co^{I}]^{0}$ /$[(bbpya^{-})Co^{I}]^{0}$** with 0.28 M CO_2 in MeCN and corresponding changes in (A) UV-Vis, and (B) EPR spectra. Blue traces correspond to **$[(Hbbpya^{-\bullet})Co^{I}]^{0}$ /$[(bbpya^{-})Co^{I}]^{0}$** and pink traces correspond to the species generated after the reaction with CO_2. EPR conditions: temperature 14 K, microwave frequency ~9.35 GHz, microwave power 0.016 mW, modulation amplitude 0.5 mT.

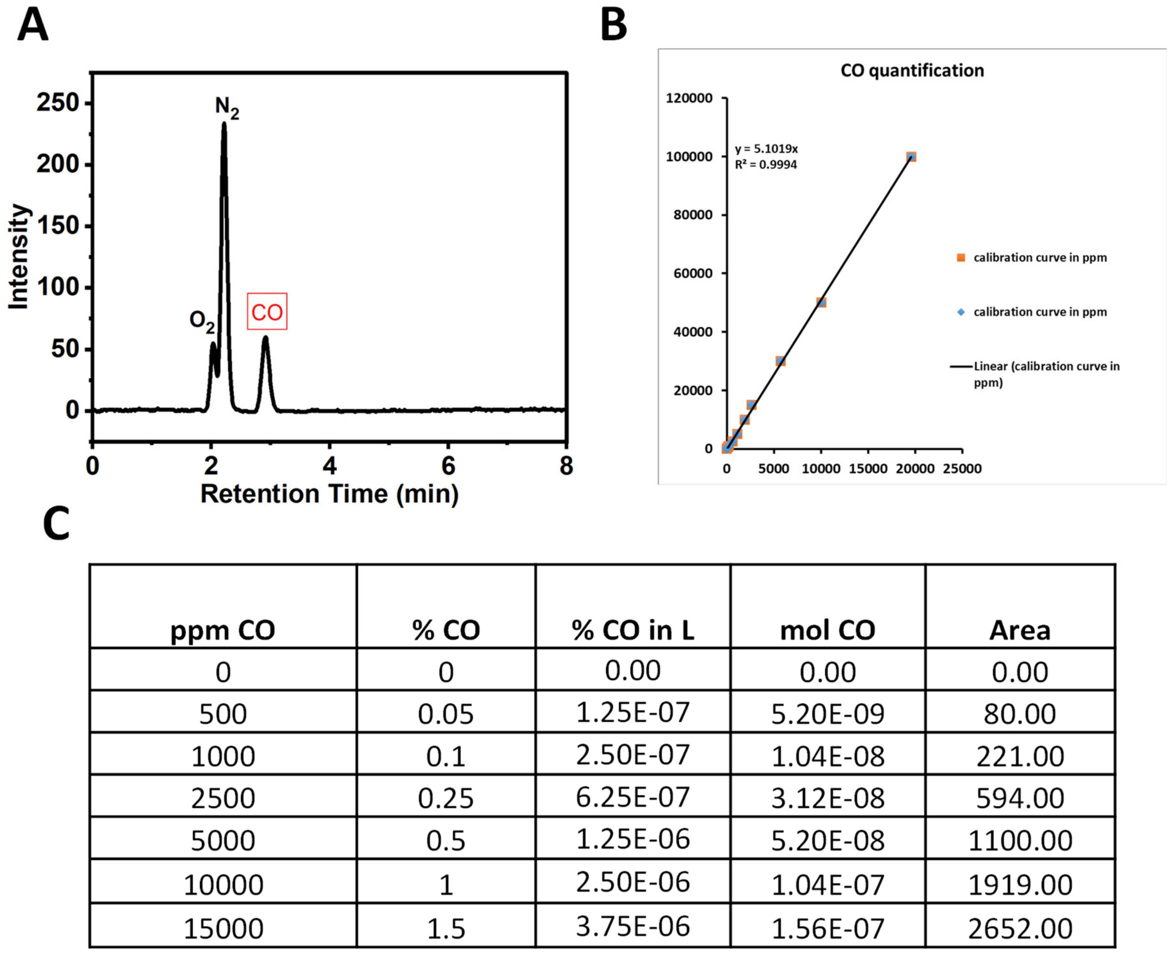

C

ppm CO	% CO	% CO in L	mol CO	Area
0	0	0.00	0.00	0.00
500	0.05	1.25E-07	5.20E-09	80.00
1000	0.1	2.50E-07	1.04E-08	221.00
2500	0.25	6.25E-07	3.12E-08	594.00
5000	0.5	1.25E-06	5.20E-08	1100.00
10000	1	2.50E-06	1.04E-07	1919.00
15000	1.5	3.75E-06	1.56E-07	2652.00

Figure S38. (A) Detected CO gas in GC from the stoichiometric reaction of **$[(Hbbpya^{\bullet-})Co^{I}]^{0}$ /$[(bbpya^{-})Co^{I}]^{0}$** with 0.28 M CO_2 in MeCN at room temperature. (B) Calibration curve used for the quantification of CO using gas chromatography. (C) Table for the quantification of CO.

Yield of CO from the reaction of $[(bbpya^{-})Co^{I}]^{0}$ / $[(Hbbpya^{\bullet-})Co^{I}]$ with CO_2:

Concentration of $[(Hbbpya)Co^{II}]^{2+}$ taken = 1 mM (2 mL).

2.2 equivalents of $CoCp_2^{*}$ were added to it.

From EPR quantification, concentration of $[(bbpya^{-})Co^{I}]^{0}$ = 0.835 mM (2 mL).

Amount of CO produced = 1.02 μmol (from the calibration curve).

% of CO formed stoichiometrically = [1.02/ (2×0.835)] × 100 = 61.07 %.

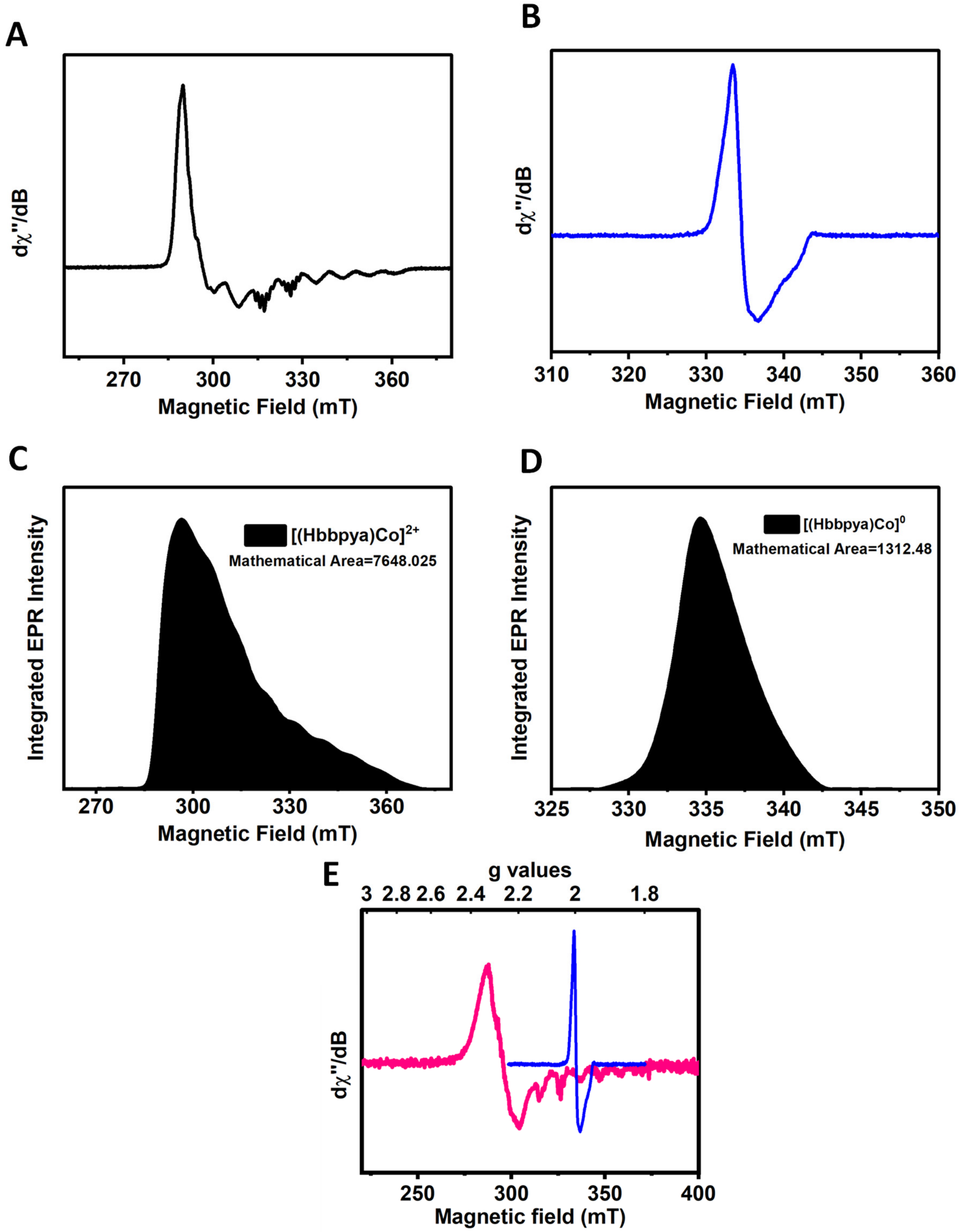

Figure S39. (A) X-band EPR spectra of 1 mM solution of **[(Hbbpya)Co^{II}]$^{2+}$** in butyronitrile. Experimental conditions: microwave frequency 9.35 GHz, microwave power 0.0025 mW, modulation amplitude 0.4 mT, temperature 13 K. (B) X-band EPR spectra of the solution after reducing 1 mM **[(Hbbpya)Co^{II}]$^{2+}$** by two equiv. of reductant keeping the EPR parameters same to form **[(Hbbpya$^{-•}$)Co^{I}]**. (C) Double integration of the signal in (A) for **[(Hbbpya)Co^{II}]$^{2+}$** gave a mathematical area of 7648.025. (D) Double integration of the signal in (B) for **[(Hbbpya$^{-•}$)Co^{I}]^{0}** gave a mathematical area of 1312.48. Thus, the spin quantification of the signals reveals the generation of **[(Hbbpya$^{-•}$)Co^{I}]^{0}** in ca. 17 % yield. (E) Comparison of the EPR spectra of **[(Hbbpya)Co^{II}CO$_3^{2-}$]** (pink) formed upon reaction of a solution of **[(Hbbpya$^{-•}$)Co^{I}]^{0}** (blue) with CO_2; hence, a two-electron oxidation is evident.

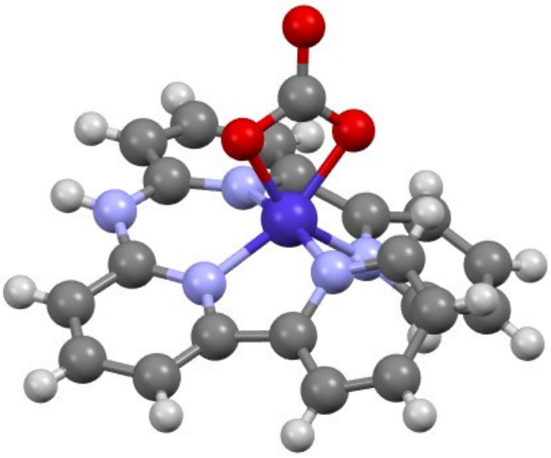

Figure S40. SC-XRD of **[(Hbbpya)Co(CO_3^{2-})]**, formed from the reaction of **[(Hbbpya$^{-•}$)Co^I]^0** and CO_2 in MeCN at room temperature.

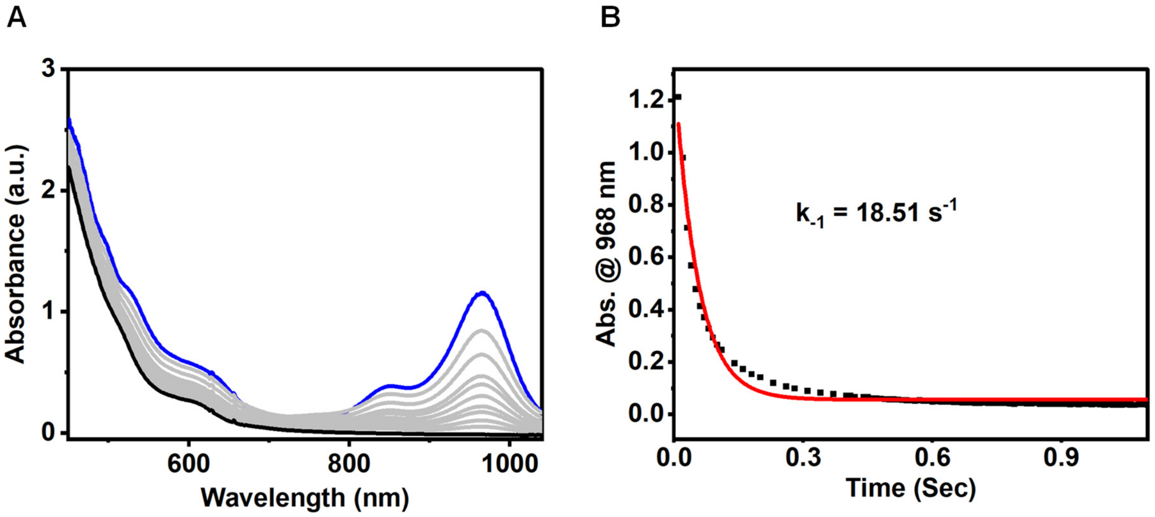

Figure S41. (A) Change in UV-Vis spectra from the reaction of generated **[(Hbbpya$^{-•}$)Co^I]^0** /**[(bbpya$^-$)Co^I]^0** (blue) with 0.28 M CO_2 in MeCN at -20 °C to form two electrons oxidised product (black) with a first order rate constant k_{-1} = 18.51 s^{-1}. (B) Time trace of the band 968 nm. Black square and red trace represent experimental data and first order fit respectively.

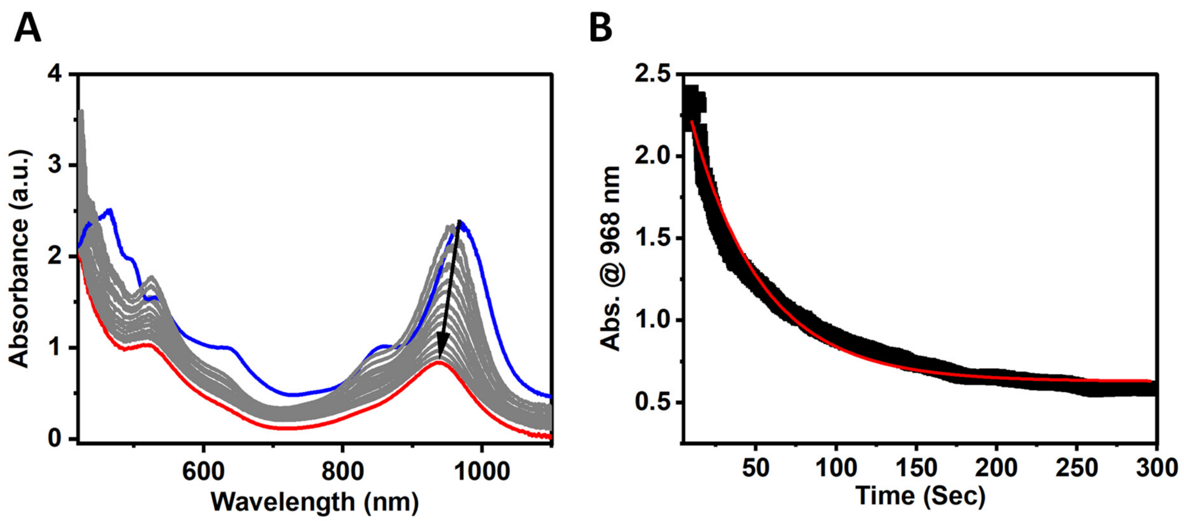

Figure S42. (A) Change in UV-Vis spectra from the reaction of generated **[(Hbbpya$^{-•}$)Co^{I}]^{0}** /**[(bbpya^{-})Co^{I}]^{0}** (blue) with 0.28 M Phenol in MeCN at -20 ºC which decays to species with similar spectral features to that of **[(Hbbpya)Co]$^{+}$** with a first order rate constant k = 0.02238 s^{-1}. (B) Time trace of the band 968 nm. Black square and red trace represent experimental data and first order fit respectively.

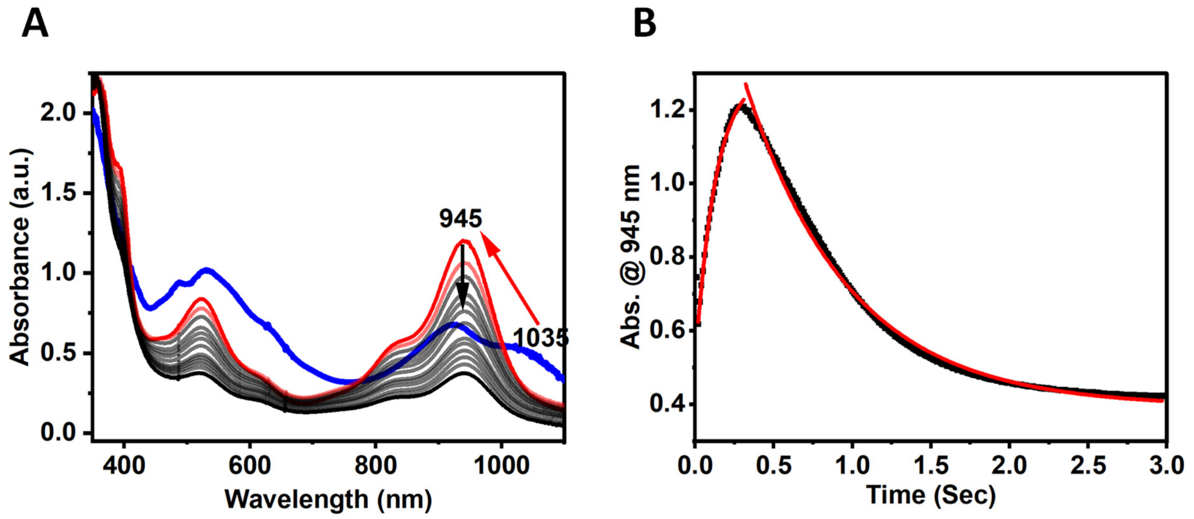

Figure S43. (A) Change in UV-Vis during the reaction of **[(Mebbpya$^{-•}$)Co^{I}]^{0}** (blue trace) with 0.28 M PhOH in MeCN at -20 ºC to initially form the one-electron oxidized species **[(Mebbpya)Co^{I}]$^{+}$**, which decays to **[(Mebbpya)Co^{II}]$^{2+}$** with the release of H_2. (B) Time trace of the band at 945 nm corresponding to **[(Mebbpya)Co^{I}]$^{+}$**, showing its initial generation with a rate constant of 7.76 s^{-1} and its decay with a rate constant of 1.51 s^{-1}. Black square and red trace represent experimental data and first order fit respectively.

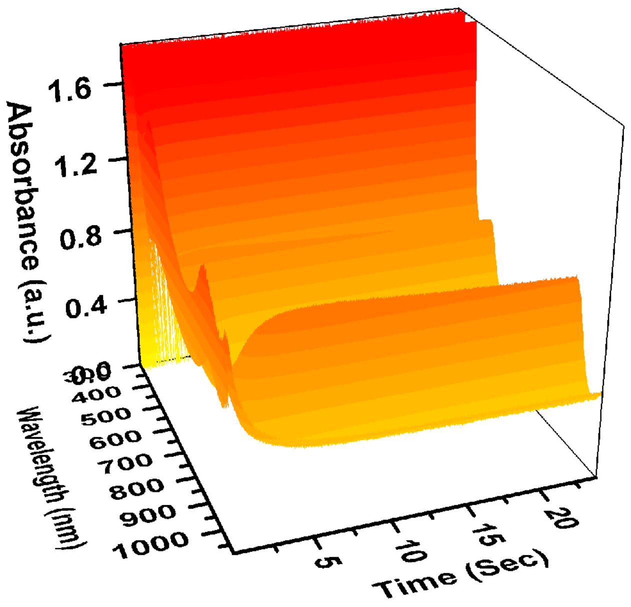

Figure S44. 3D profile for the Change in UV-Vis during the reaction of **[(Mebbpya$^{\bullet}$)Co^{I}]^{0}** with 0.28 M CO_2 under stopped-flow conditions. Selected 2D spectra and the time trace (945 nm) are shown in Figure 3.

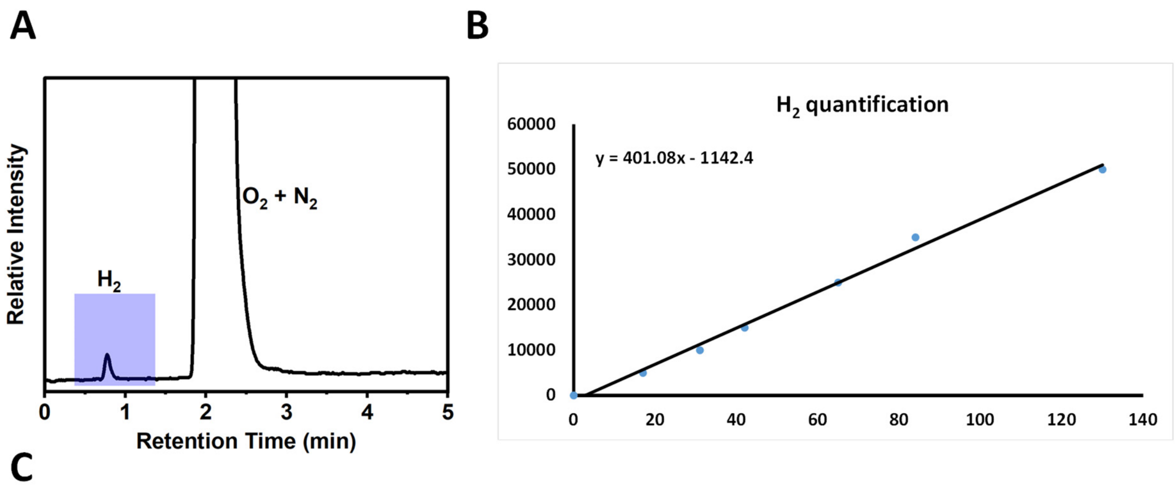

C

ppm H_2	% H_2	V H_2 in L	mol H_2	Area
0	0	0.00	0.00	0.00
5000	0.5	1.25E-06	5.58E-08	17.00
10000	1	2.50E-06	1.12E-07	31.00
15000	1.5	3.75E-06	1.67E-07	42.00
25000	2.5	6.25E-06	2.79E-07	65.00
35000	3.5	8.75E-06	3.91E-07	84.00
50000	5	1.25E-05	5.58E-07	130.00

Figure S45. (A) Detected H_2 gas in GC from the stoichiometric reaction of **$[(Mebbpya^{-•})Co^{I}]^{0}$** with 0.28 M PhOH in MeCN at room temperature. (B) Calibration curve used for the quantification of H_2 using gas chromatography. (C) Table for the quantification of H_2.

Yield of H_2 from the reaction of $[(Mebbpya^{-•})Co^{I}]^{0}$ with PhOH:

Amount of $[(Mebbpya)Co^{II}]^{2+}$ (MW: 737.515) taken = 8 mg in 8 mL (10.84 µmol).

2.2 equivalents of $CoCp_2^*$ were added to it.

Amount of H_2 produced = 8.24 µmol (from the calibration curve).

% of H_2 formed stoichiometrically = [8.24/10.84] × 100 = 76.29%.

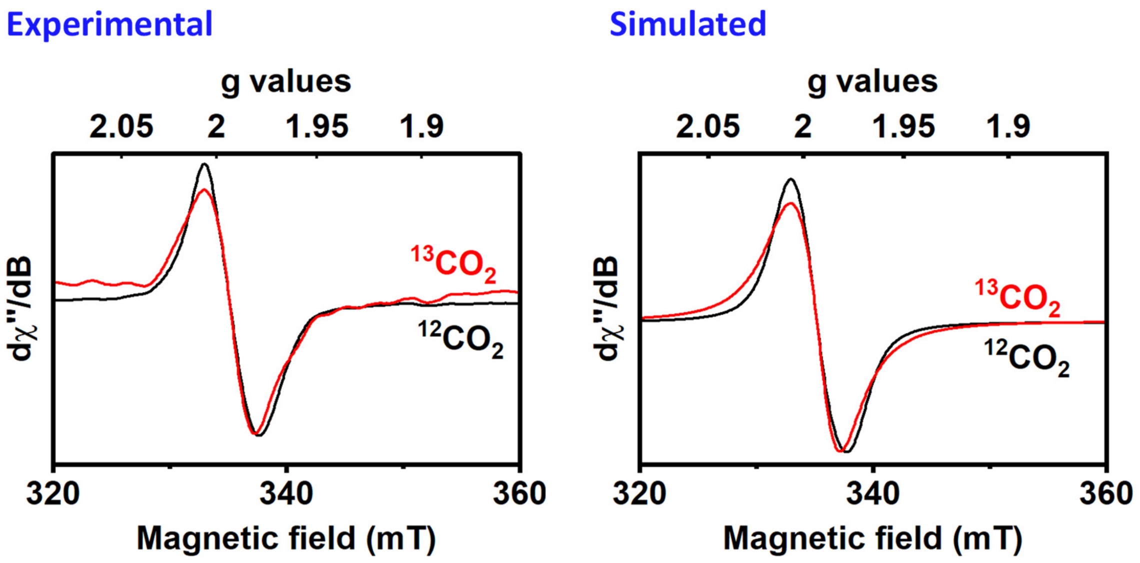

Figure S46. (Left) Experimental EPR spectra of **[(Mebbpya)Co^{I}-$CO_2^{\bullet-}$]** obtained at 0.2 s after addition of $^{12}CO_2$ (black) and $^{13}CO_2$ (red) in butyronitrile at 14 K. (Right) Simulations of the experimental EPR spectra of **[(Mebbpya)Co^{I}-$CO_2^{\bullet-}$]** obtained at 0.2 s after addition of $^{12}CO_2$ (black) and $^{13}CO_2$ (red). The following parameters were used for the simulations: (for $^{12}CO_2$) $g_x = 2.008$, $g_y = 1.994$, $g_z = 1.972$, lwpp = [1.5014, 1.8014] mT; (for $^{13}CO_2$) $g_x = 2.008$, $g_y = 1.994$, $g_z = 1.972$, lwpp = [1.5014, 1.8114] mT, $|A(^{13}C)|$ = [0, 21, 69] MHz.

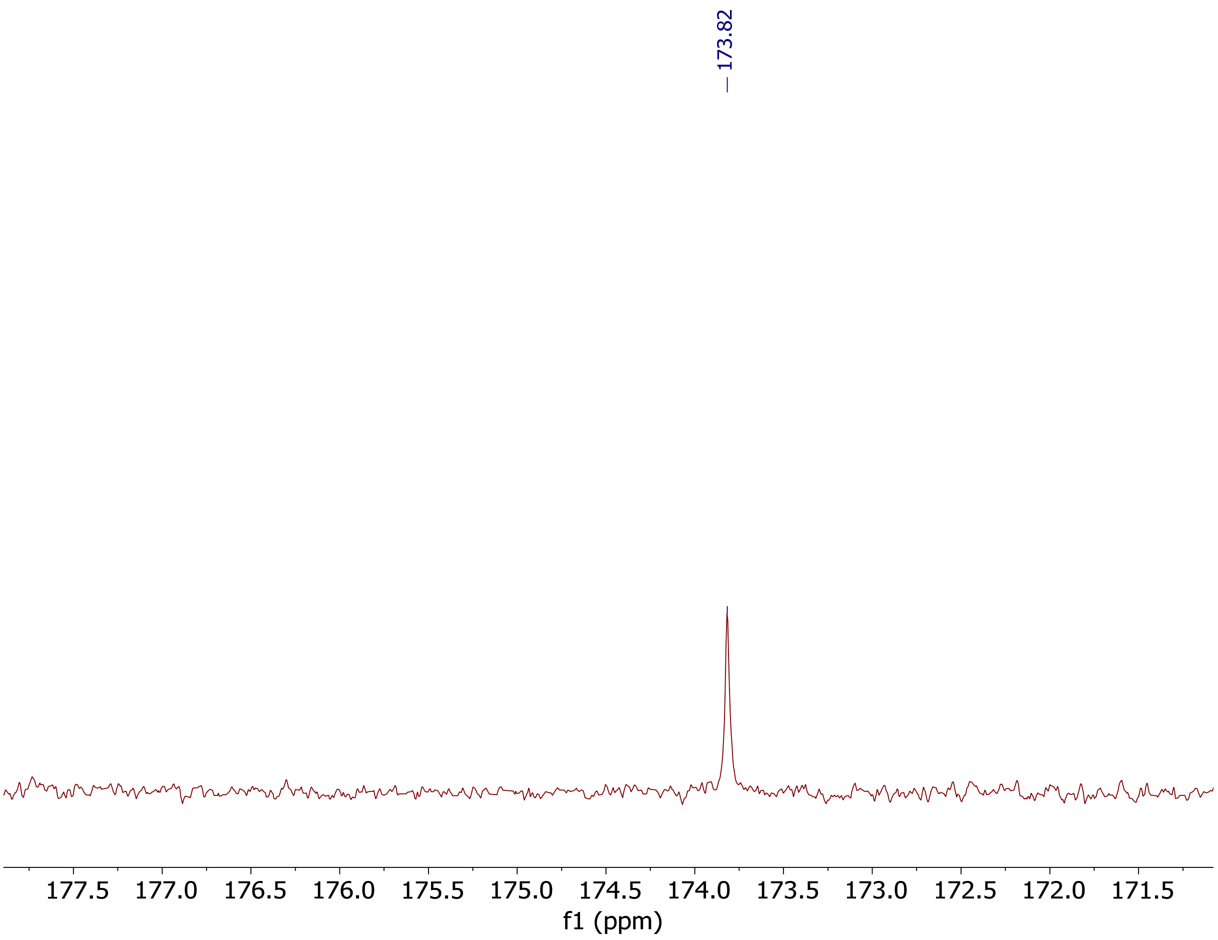

Figure S47. ^{13}C NMR spectrum in D_2O after the extraction of the precipitates from the reaction of **[(Mebbpya^{-})Co^{I}]^{0}** and CO_2.

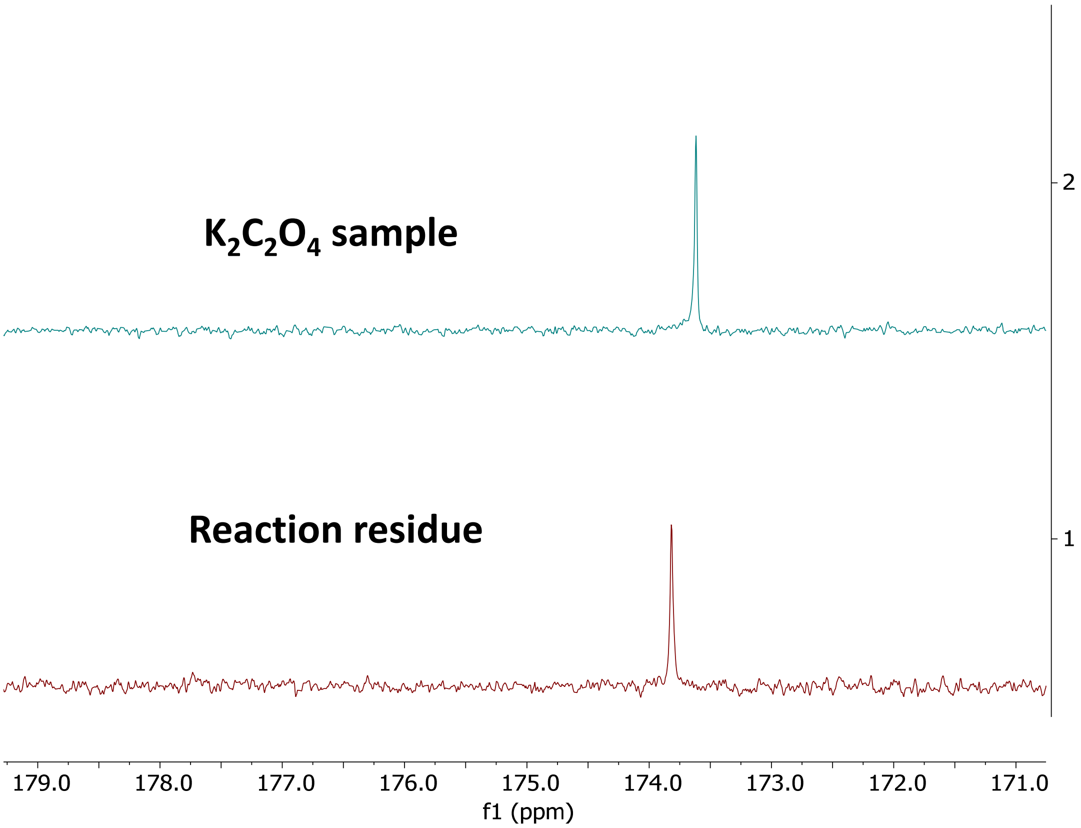

Figure S48. Comparison of ^{13}C NMR spectrum of authentic $K_2C_2O_4$ and that of the solid residues in D_2O after aqueous extraction of the precipitate from the reaction of **[(Mebbpya^{-})Co^{I}]^{0}** and CO_2.

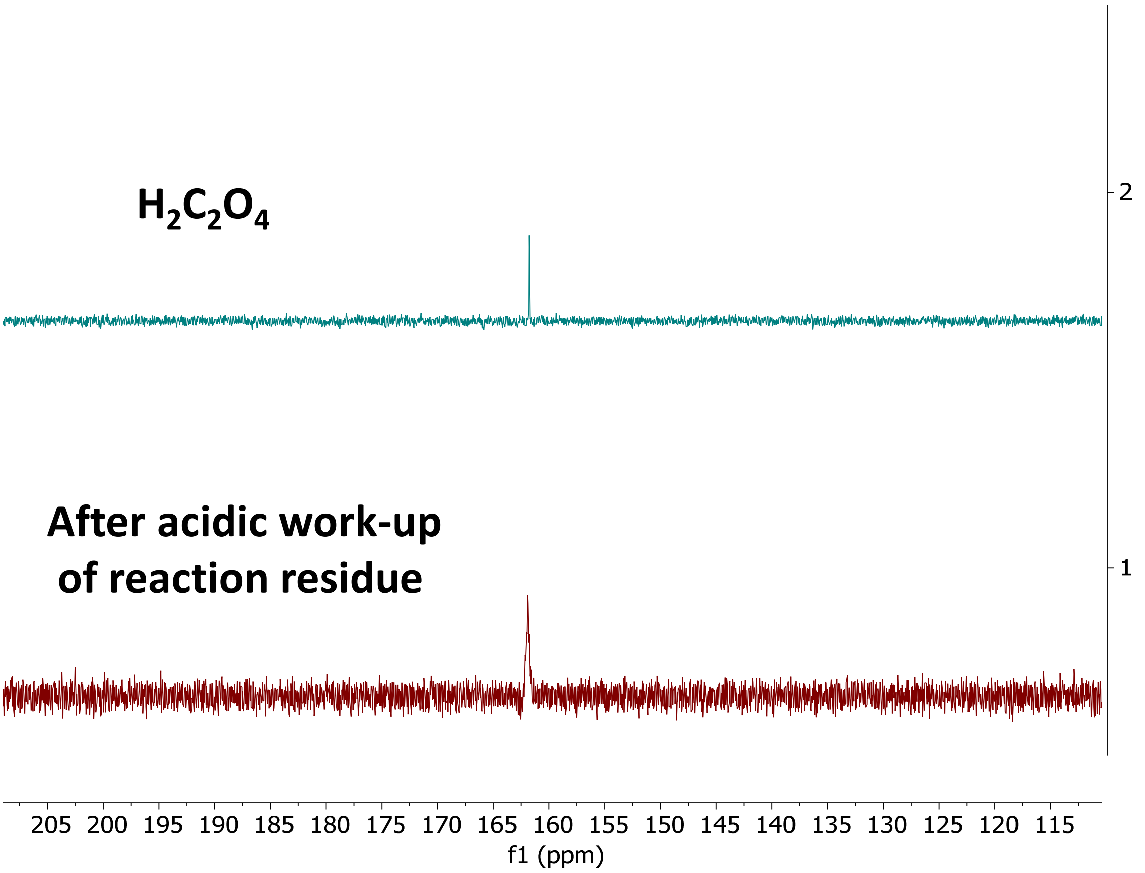

Figure S49. Comparison of ^{13}C NMR spectrum of authentic $H_2C_2O_4$ and that of the solid residues in D_2O after acidic (1 M HCl) work-up of the precipitate from the reaction of **[(Mebbpya$^-$)Co^I]^0** and CO_2.

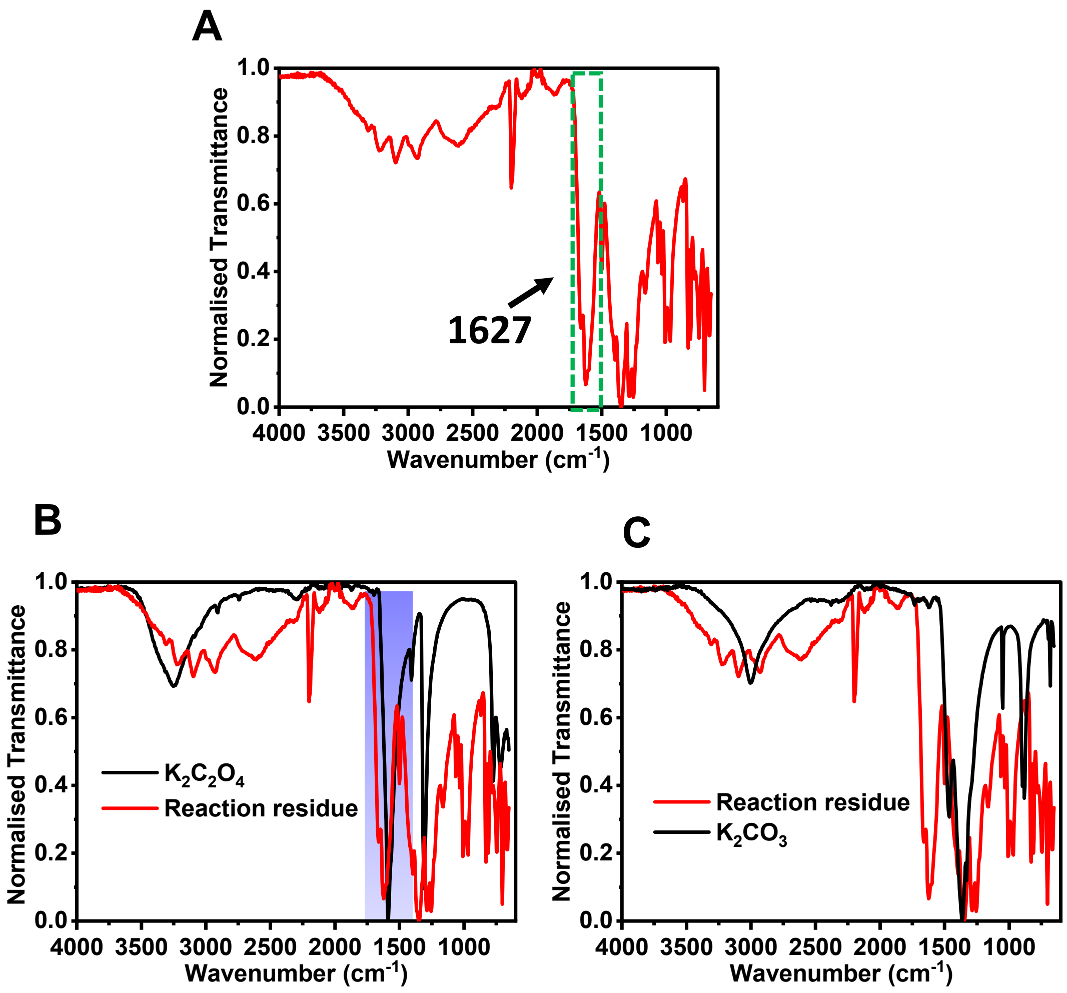

Figure S50. (A) ATR-IR spectrum of the residue from the reaction of **[(Mebbpya⁻)Co^{I}]^{0}** and CO_2 (red). Peak at 1627 cm^{-1} corresponds to asymmetric C-O stretching mode of the carboxylate functional group of $K_2C_2O_4$. (B) The comparison of IR spectra of authentic $K_2C_2O_4$ sample (black) and reaction residue (red). (C) The comparison of IR spectra of authentic K_2CO_3 sample (black) and reaction residue (red) which shows the absence of 1627 cm^{-1} peak in K_2CO_3.

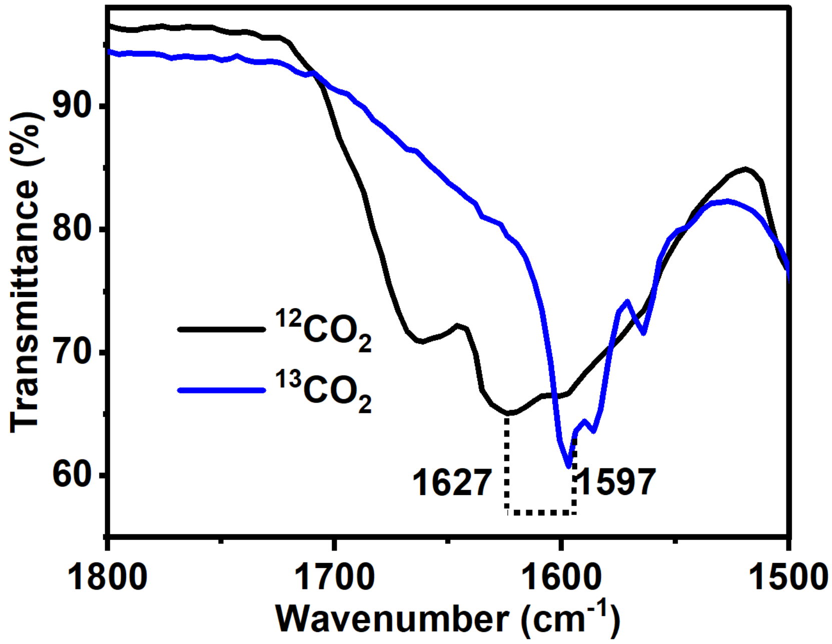

Figure S51. (A) ATR-IR spectrum of the residue from the reaction of **[(Mebbpya$^-$)Co^I]^0** and $^{12}CO_2$ (black) and $^{13}CO_2$ (blue). Peak at 1627 cm^{-1} corresponds to asymmetric C-O stretching mode of the carboxylate functional group of $K_2{}^{12}C_2O_4$ shifts to 1597 cm^{-1} in $K_2{}^{13}C_2O_4$.

a)

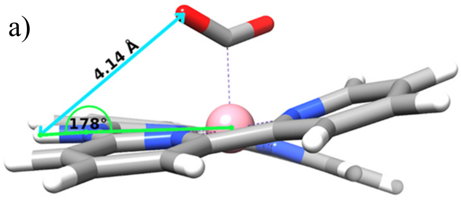

b)

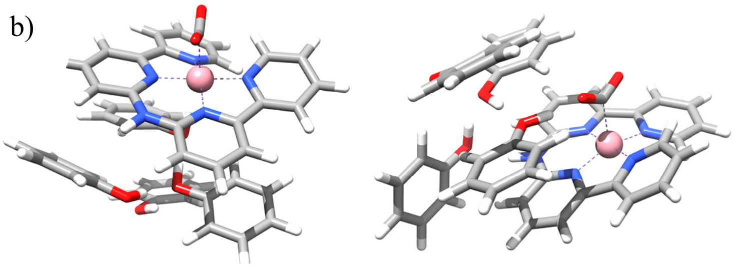

[1-Co-CO$_2$]^o + HA

[1-Co-CO$_2$]$^o_{rearr.}$ + HA

Figure S52. a) DFT-optimized structure highlighting the orientation of the equatorial amine (N–H) bond relative to the coordinated CO_2 ligand. The nearly linear Heq. amine–Neq. amine–Co angle (178.8°) and the significant separation between the N–H hydrogen and the nearest CO_2 oxygen atom (~4.14 Å) indicate that the N–H bond points away from the CO_2 ligand; b) DFT optimized structures for the CO_2 binding states in Figure 4.

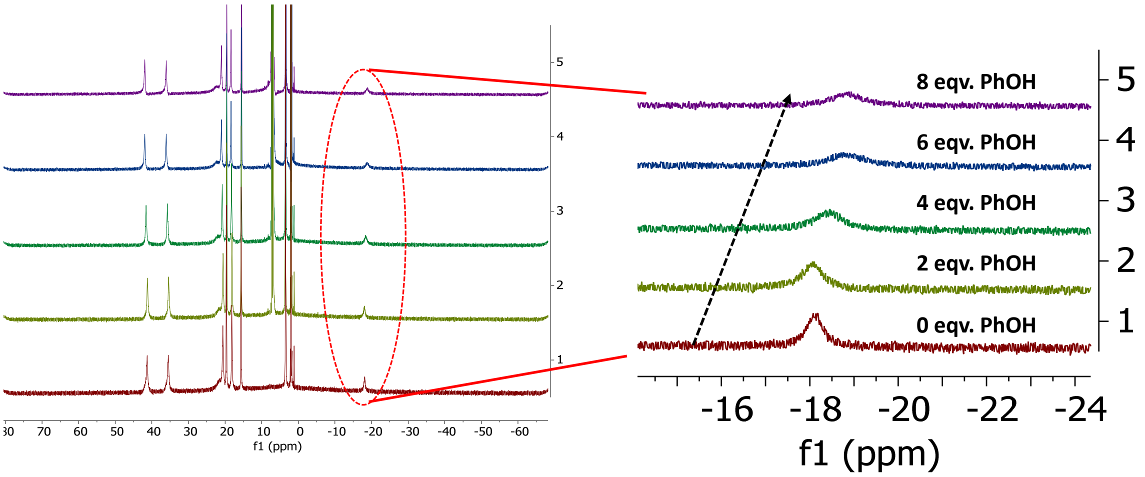

Figure S53. ^{1}H-NMR spectra of **[(Hbbpya)Co^{II}]$^{2+}$** in CD_3CN at 298 K showing the shift of the -NH proton of **[(Hbbpya)Co^{II}]$^{2+}$** with different equivalents of PhOH.

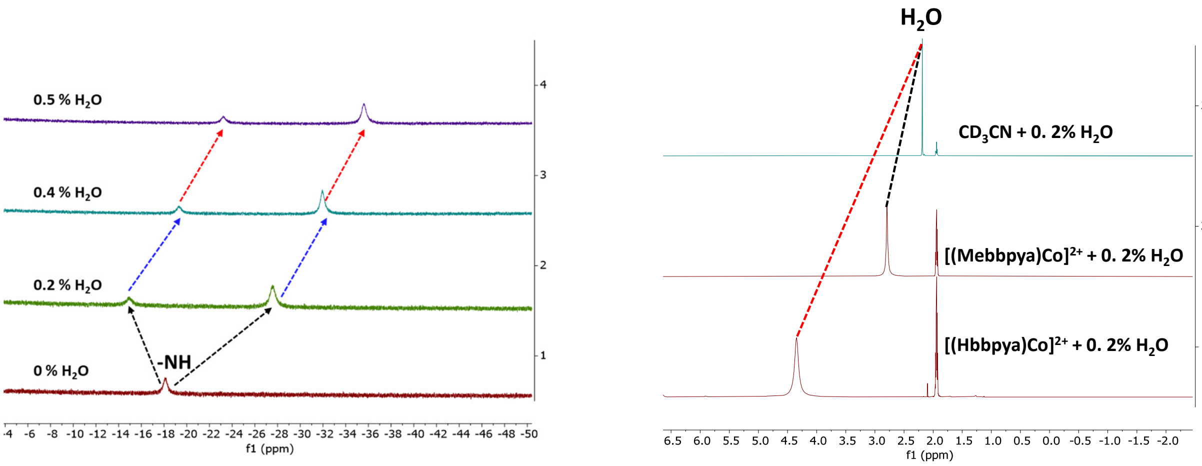

Figure S54. (Left) ^{1}H-NMR spectra in CD_3CN at 298 K showing the shift of the -NH proton of **[(Hbbpya)Co^{II}]$^{2+}$** with different amount of water; (Right) ^{1}H NMR of H_2O in presence of **[(Hbbpya)Co^{II}]$^{2+}$** and **[(Mebbpya)Co^{II}]$^{2+}$**.

7 Supplementary Tables

Table S1. Crystallographic Data[9] for **[(Hbbpya)Co^{II}]$^{2+}$**, **[(Mebbpya)Co^{II}]$^{2+}$**, and **[(bbpya^{-})Co^{II}]$^{+}$**

	**[(Hbbpya)Co]$^{2+}$**	**[(Mebbpya)Co]$^{2+}$**	**[(bbpya^{-})Co^{II}]$^{+}$**
CCDC number	2384008	2384009	2384005
Empirical formula	C_{22} H_{15} Co F_6 N_5 O_6 S_2,2(C_2 H_3 N)	C_{24} H_{20} Co F_3 N_6 O_3 S, C F_3 O_3 S	$C_{23}H_{17}CoF_3N_6O_3$ S
Formula weight	764.55	739.59	573.41
Temperature (K)	100(2)	100(2)	100(2)
Wavelength (Å)	0.71073	0.71073	0.71073
Crystal system/space group	triclinic, P-1	Monoclinic, $P2_1/n$	Monoclinic, $P2_1/n$
Crystal size (mm)	0.29×0.13×0.07	0.46×0.35×0.27	0.217×0.07×0.041
a (Å)	8.3098(8)	13.0009(7)	10.5076(10)
b (Å)	12.8944(12)	18.3888(12)	8.1255(8)
c (Å)	15.2143(13)	13.1096(8)	26.785(3)
α (°)	103.426(3)	90	90
β (°)	100.920(3)	105.794(2)	96.258(4)
γ (°)	103.382(3)	90	90
Volume/Å^3	1490.8(2)	3015.8(3)	2273.3(4)
Z	2	4	4
ρ_{calc}(g/cm^3)	1.703	1.6244	1.675
F(000)	774.0	1492.0	1164.0
μ/mm^{-1}	0.809	0.795	0.913
Data/restraints/parameters	7454/0/435	9808/0/417	10443/0/335
Goodness-of-fit on F^2	1.034	1.021	1.085
Final R indexes [all data]	R_1 = 0.0248, wR_2 = 0.0675	R_1 = 0.0402, wR_2 = 0.0882	R_1 = 0.0844, wR_2 = 0.2033

Table S2. Crystallographic Data[9] for **[(Mebbpya)Co^{I}]$^{+}$** and **[(bbpya^{-})Co^{I}]^{0}**

	**[(Mebbpya)Co^{I}]$^{+}$**	**[(bbpya^{-})Co^{I}]^{0}**
CCDC number	2384007	2384011
Empirical formula	$C_{22}H_{17}CoF_3N_5O_3S$	$C_{21}H_{18}CoN_5$
Formula weight	547.40	399.344
Temperature (K)	100(2)	100(2)
Wavelength (Å)	0.71073	0.71073
Crystal system/space group	Monoclinic P21/c	Monoclinic, $P2_1/n$
Crystal size (mm)	0.12×0.1×0.05	0.09×0.04×0.02
a (Å)	27.856(7)	13.309(2)
b (Å)	7.2291(14)	25.473(4)
c (Å)	24.638(5)	13.730(3)
α (°)	90	90
β (°)	123.513(5)	90.07(3)
γ (°)	90	90
Volume/Å^3	1490.8(2)	4654.6(14)
Z	8	4
ρ_{calc}(g/cm^3)	1.758	1.121
F(000)	2224.0	2352
μ/mm^{-1}	0.997	0.913
Data/restraints/parameter	7562/0/633	8266/0/704
Goodness-of-fit on F^2	1.034	1.020
Final R indexes [all data]	R_1 = 0.1237, wR_2 = 0.2746	R_1 = 0.0529, wR_2 = 0.1187

Table S3. Crystallographic Data[9] for **[(Hbbpya)Zn^{II}]$^{2+}$** and **[(Mebbpya)Zn^{II}]$^{2+}$**

	**[(Hbbpya)Zn^{II}]$^{2+}$**	**[(Mebbpya)Zn^{II}]$^{2+}$**
CCDC number	2384004	2384010
Empirical formula	C_{22} H_{15}Zn F_6 N6 O5 S_2	$C_{23}H_{17}$ Zn F_6 N_5 O_6 S_2
Formula weight	688.90	702.908
Temperature (K)	100(2)	100(2)
Wavelength (Å)	0.71073	0.71073
Crystal system/space group	Monoclinic, $P2_1/c$	Triclinic P-1
Crystal size(mm)	0.03×0.07×0.14	0.23×0.20×0.16
a (Å)	14.617(4)	10.3098(4)
b (Å)	13.512(4)	10.3325(5)
c (Å)	13.815(4)	13.0850(6)
α (°)	90	108.031(2)
β (°)	114.445(7)	101.057(2)
γ (°)	90	100.784(2)
Volume/Å^3	2483.9(12)	1254.65(10)
Z	4	12
ρ_{calc}(g/cm^3)	1.842	1.861
F(000)	1384.0	708
μ/mm^{-1}	1.254	1.244
Data/restraints/parameters	5928/0/379	12695/0/389
Goodness-of-fit on F^2	1.081	1.031
Final R indexes [all data]	R_1 = 0.0871, wR_2 = 0.2188	R_1 = 0.0277, wR_2 = 0.0662

Table S4. Comparison of the bond distances (in Å) for **[(Mebbpya)Co^{II}]$^{2+}$** and **[(Mebbpya)Co^{I}]$^{+}$**

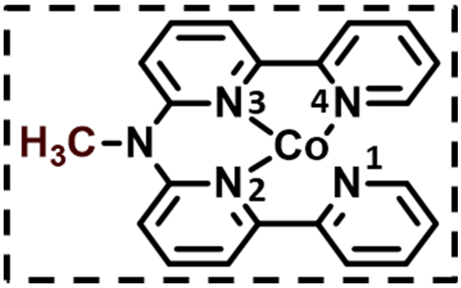

**Selected bonds**	**[(Mebbpya)Co^{II}]$^{2+}$**	**[(Mebbpya)Co^{I}]$^{+}$**
Co-N1	1.980(1)	1.919(6)
Co-N2	1.916(1)	1.864(6)
Co-N3	1.928(1)	1.853(4)
Co-N4	1.960(1)	1.888(4)

8 DFT calculations

All calculations in this work utilized the ORCA program package version 5.0.3/5.0.4[10-13] (utilizing libint2[14] and libXC version 5.1.0[15]).

Electronic structures calculations on the DFT level have been conducted using the four different DFT-functionals BP86[16-17], TPSSh[18], B3LYP16,19[19] and PBE0[20]. During these calculations the def2-TZVP(-f)[21] basis set has been used as main basis set while the AutoAux22[22] feature has been invoked to fit auxiliary basis sets to utilize the RI[23-29] and RIJCOSX[30] approximation for GGA- and hybrid-functionals, respectively. Dispersion effects have been accounted for by the D3[21] dispersion correction with Becke-Johnson damping[31]. The influence of solvation effects has been modeled by the C-PCM[32] for acetonitrile. Furthermore, the keywords TightOpt and TightSCF have been given to ensure tight convergence, and optimized geometric minima have been verified by calculation of the Hessian, which showed no significant (<-31 cm**-1) imaginary frequency. G- and a-tensors have been calculated with linear response theory on the very same level of theory. Corresponding energies, state energy differences in kcal/mol and electronic state characteristics as deduced from Loewdin spin populations are shown in the tables below.

Although some of the identified electronic low-spin states may suggest to be found using the broken-symmetry DFT formalism, the entirety of the results presented below have been obtained by simply setting the multiplicity to the corresponding low spin solution and doing a standard UKS DFT calculation. Nevertheless, where appropriate, broken-symmetry calculations have been conducted to confirm that the identified low-spin state represents the ground state and to ascertain whether other low-lying low-spin states are accessible. Be that as it may, no new states have been identified through this approach.

As previously stated in the manuscript, two distinct structures were identified for the doubly reduced complexes using different functionals, which also yielded different electronic states. This leads to the question of whether the different electronic states originate from the distinct geometries or vice versa. To address this question, a series of test calculations has been conducted, combining different input geometries, orbitals, and functional choices. E.g., a single point calculation with the BP86 functional on the converged B3LYP geometry, without reading in any orbitals, again yields a low spin Co(I) + L*. Single point calculations with the B3LYP functional, reading in BP86 orbitals ("low spin Co(I) + L*"-orbitals), on both B3LYP and BP86 geometries yielded identical results. This suggests that the choice of functional exerts the most significant influence on the electronic structure to which the calculation converges.

Table S5. Final Gibbs free energy, energy differences and interpreted electronic state from Loewdin analysis spin population, spin densities and orbital occupation numbers and energies for all four different functionals for **[(Hbbpya)Co]**^{x}. For the last section, LS = low spin, HS = high spin, L- = ligand radical, L2- = ligand diradical and a tilde (~) indicates that the assigned state is a very rough estimate.

E [Hartree]	$[(Hbbpya)Co]^{2+}$		$[(Hbbpya)Co]^{+}$		$[(Hbbpya)Co]^{0}$		$[(bbpya)Co]^{0}$	
	S=1/2	S=3/2	S=0	S=1	S=1/2	S=3/2	S=0	S=1
BP86	-4352.0169	-4351.9914	-2693.7447	-2693.7308	-2693.8689	-2693.8409	-2693.2823	-2693.2695
TPSSH	-4351.6748	-4351.6681	-2693.5374	-2693.5344	-2693.6411	-2693.6394	-2693.0712	-2693.0694
B3LYP	-4350.3840	-4350.3814	-2692.6081	-2692.6065	-2692.7142	-2692.7125	-2692.1434	-2692.1416
**Δ**E [kcal mol^{-1}]	$[(Hbbpya)Co]^{2+}$		$[(Hbbpya)Co]^{+}$		$[(Hbbpya)Co]^{0}$		$[(bbpya)Co]^{0}$	
	S=1/2	S=3/2	S=0	S=1	S=1/2	S=3/2	S=0	S=1
BP86	0.0	16.0	0.0	8.7	0.0	17.6	0.0	8.0
TPSSH	0.0	4.2	0.0	1.9	0.0	1.1	0.0	1.1
B3LYP	0.0	1.6	0.0	1.0	0.0	1.1	0.0	1.1
electronic state	$[(Hbbpya)Co]^{2+}$		$[(Hbbpya)Co]^{+}$		$[(Hbbpya)Co]^{0}$		$[(bbpya)Co]^{0}$	
	S=1/2	S=3/2	S=0	S=1	S=1/2	S=3/2	S=0	S=1
BP86	Co(II) LS	Co(II) HS	Co(I) LS	Co(I) HS	Co(I) LS + L-	~Co(I) HS + L-	Co(I) LS	Co(II) LS + L-
TPSSH	Co(II) LS	Co(II) HS	Co(II) LS + L-	Co(II) LS + L-	Co(II) LS + L2-	~Co(II) LS + L2-	Co(II) LS + L-	Co(II) LS + L-
B3LYP	Co(II) LS	Co(II) HS	Co(II) LS + L-	Co(II) LS + L-	Co(II) LS + L2-	~Co(II) LS + L2-	Co(II) LS + L-	~Co(II) LS + L-

Table S6. Calculated Geometric parameters of **[(Hbbpya)Co]$^{2+}$** and **[(Mebbpya)Co]$^{2+}$** including acetonitrile in the axial positions. N1-N4 being to the Co coordinating ones, N5 being the bridging N.

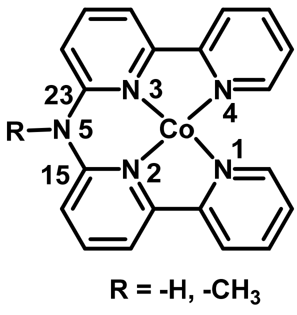

S=1/2			S=3/2		
BP86 (ACN)	[(Hbbpya)Co]$^{2+}$	[(Mebbpya)Co]$^{2+}$	BP86 (ACN)	[(Hbbpya)Co]$^{2+}$	[(Mebbpya)Co]$^{2+}$
Co-N1	1.955	1.988	Co-N1	2.111	2.093
Co-N2	1.911	1.937	Co-N2	2.059	2.079
Co-N3	1.914	1.928	Co-N3	2.058	2.067
Co-N4	1.935	1.962	Co-N4	2.112	2.100
Co-N5	3.193	3.191	Co-N5	3.294	3.309
C15-N5	1.377	1.390	C15-N5	1.383	1.405
C23-N5	1.377	1.397	C23-N5	1.383	1.398
B3LYP (ACN)	[(Hbbpya)Co]$^{2+}$	[(Mebbpya)Co]$^{2+}$	B3LYP (ACN)	[(Hbbpya)Co]$^{2+}$	[(Mebbpya)Co]$^{2+}$
Co-N1	1.982	1.953	Co-N1	2.136	2.127
Co-N2	1.941	1.909	Co-N2	2.089	2.100
Co-N3	1.940	1.899	Co-N3	2.086	2.093
Co-N4	1.983	1.923	Co-N4	2.133	2.132
Co-N5	3.205	3.200	Co-N5	3.305	3.298
C15-N5	1.375	1.386	C15-N5	1.380	1.397
C23-N5	1.375	1.395	C23-N5	1.380	1.403

Table S7. Comparison of bond distances (in Å) in **[(Hbbpya)Co^{II}]$^{2+}$** and **[(Mebbpya)Co^{II}]$^{2+}$** as determined from their molecular structures (Scheme 1) and DFT calculations (BP86 functional). N1-N4 being to the Co coordinating ones, N5 being the bridging N.

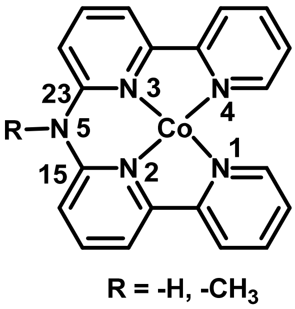

Selected bonds		[(Hbbpya)Co^{II}]$^{2+}$	[(Mebbpya)Co^{II}]$^{2+}$
Co-N1	XRD	1.968(1)	1.980(1)
	DFT	1.955	1.988
Co-N2	XRD	1.924(1)	1.916(1)
	DFT	1.911	1.937
Co-N3	XRD	1.921(1)	1.928(1)
	DFT	1.914	1.928
Co-N4	XRD	1.974(1)	1.960(1)
	DFT	1.935	1.962
Co-N5	XRD	3.219(1)	3.202(1)
	DFT	3.193	3.191
C15-N5	XRD	1.372(2)	1.394(2)
	DFT	1.377	1.390
C23-N5	XRD	1.375(2)	1.392(2)
	DFT	1.377	1.397

Table S8. *g*-tensors for **[(Hbbpya)Co]^{x}** calculated with the two different functionals BP86 and B3LYP.

	BP86								B3LYP							
	[(Hbbpya)Co]+2OTf		[(Hbbpya)Co]$^{2+}$		[(Hbbpya)Co]$^{+}$		[(Hbbpya)Co]^{0}		[(Hbbpya)Co]+2OTf		[(Hbbpya)Co]$^{2+}$		[(Hbbpya)Co]$^{+}$		[(Hbbpya)Co]^{0}	
total	S=1/2	S=3/2	S=1/2	S=3/2	S=0	S=1	S=1/2	S=3/2	S=1/2	S=3/2	S=1/2	S=3/2	S=0	S=1	S=1/2	S=3/2
g1	2.007	2.055	2.008	2.044	/	2.017	1.987	2.000	2.007	2.080	2.007	2.066	/	2.002	2.006	1.999
g2	2.101	2.064	2.090	2.066	/	2.053	1.998	2.039	2.162	2.088	2.140	2.093	/	2.086	2.174	2.065
g3	2.138	2.091	2.120	2.099	/	2.069	2.007	2.050	2.189	2.130	2.156	2.137	/	2.101	2.218	2.078
delta																
g1	0.005	0.052	0.005	0.041	/	0.014	-0.016	-0.002	0.004	0.077	0.004	0.064	/	0.000	0.004	-0.003
g2	0.099	0.061	0.088	0.064	/	0.051	-0.004	0.037	0.159	0.086	0.137	0.090	/	0.083	0.171	0.062
g3	0.136	0.089	0.118	0.096	/	0.067	0.005	0.048	0.186	0.128	0.154	0.135	/	0.098	0.216	0.076

Table S9. Final Gibbs free energy, energy differences and interpreted electronic state from Loewdin analysis spin population, spin densities and orbital occupation numbers and energies for all four different functionals for **[(Mebbpya)Co]^{x}**, For the last section, LS = low spin, HS = high spin, L- = ligand radical, L2- = ligand diradical and a tilde (~) indicates that the assigned state is a very rough estimate.

E [Hartree]	[(Mebbpya)Co]$^{2+}$		[(Mebbpya)Co]$^{+}$		[(Mebbpya)Co]^{0}	
	S=1/2	S=3/2	S=0	S=1	S=1/2	S=3/2
BP86	-4391.3019	-4391.2750	-2733.0328	-2733.0185	-2733.1539	-2733.1244
TPSSH	-4390.9635	-4390.9541	-2732.8285	-2732.8249	-2732.9337	-2732.9278
B3LYP	-4389.6386	-4389.6382	-2731.8673	-2731.8651	-2731.9725	-2731.9708

ΔE [kcal mol^{-1}]	[(Mebbpya)Co]$^{2+}$		[(Mebbpya)Co]$^{+}$		[(Mebbpya)Co]^{0}	
	S=1/2	S=3/2	S=0	S=1	S=1/2	S=3/2
BP86	0.0	16.9	0.0	9.0	0.0	18.5
TPSSH	0.0	5.9	0.0	2.3	0.0	3.7
B3LYP	0.0	0.3	0.0	1.4	0.0	1.1

electronic state	[(Mebbpya)Co]$^{2+}$		[(Mebbpya)Co]$^{+}$		[(Mebbpya)Co]^{0}	
	S=1/2	S=3/2	S=0	S=1	S=1/2	S=3/2
BP86	Co(II) LS	Co(II) HS	Co(I) LS	Co(I) HS	Co(I) LS + L-	Co(II) LS + L2-
TPSSH	Co(II) LS	Co(II) HS	Co(II) LS + L-	~Co(II) LS + L-	Co(II) LS + L2-	Co(II) LS + L2-
B3LYP	Co(II) LS	Co(II) HS	Co(II) LS + L-	~Co(II) LS + L-	Co(II) LS + L2-	Co(II) LS + L2-

Table S10. *g*-tensors for **[(Mebbpya)Co]^{x}** calculated with the two different functionals BP86 and B3LYP.

	BP86								B3LYP							
	[(Mebbpya)Co]+2OTf		[(Mebbpya)Co]$^{2+}$		[(Mebbpya)Co]$^{+}$		[(Mebbpya)Co]^{0}		[(Mebbpya)Co]+2OTf		[(Mebbpya)Co]$^{2+}$		[(Mebbpya)Co]$^{+}$		[(Mebbpya)Co]^{0}	
total	S=1/2	S=3/2	S=1/2	S=3/2	S=0	S=1	S=1/2	S=3/2	S=1/2	S=3/2	S=1/2	S=3/2	S=0	S=1	S=1/2	S=3/2
g1	2.007	2.055	2.008	2.046	/	2.018	1.989	1.997	2.007	2.080	2.008	2.068	/	1.999	2.011	1.998
g2	2.103	2.064	2.093	2.068	/	2.057	2.000	2.039	2.163	2.090	2.150	2.097	/	2.084	2.149	2.061
g3	2.139	2.088	2.127	2.093	/	2.075	2.007	2.050	2.191	2.127	2.181	2.129	/	2.100	2.212	2.073
delta																
g1	0.005	0.053	0.006	0.043	/	0.016	-0.014	-0.005	0.004	0.078	0.005	0.066	/	-0.003	0.009	-0.005
g2	0.101	0.062	0.091	0.066	/	0.054	-0.002	0.037	0.161	0.088	0.148	0.095	/	0.081	0.147	0.059
g3	0.137	0.086	0.125	0.091	/	0.073	0.005	0.048	0.189	0.125	0.179	0.127	/	0.098	0.210	0.071

Table S11. *A*-tensors for Co in **[(Hbbpya)Co]^x** calculated with the two different functionals BP86 and B3LYP.

	BP86								B3LYP							
	[(Hbbpya)Co]+2OTf		[(Hbbpya)Co]$^{2+}$		[(Hbbpya)Co]$^{+}$		[(Hbbpya)Co]^{0}		[(Hbbpya)Co]+2OTf		[(Hbbpya)Co]$^{2+}$		[(Hbbpya)Co]$^{+}$		[(Hbbpya)Co]^{0}	
total	S=1/2	S=3/2	S=1/2	S=3/2	S=0	S=1	S=1/2	S=3/2	S=1/2	S=3/2	S=1/2	S=3/2	S=0	S=1	S=1/2	S=3/2
a1	76.20	37.62	35.31	26.69	/	24.37	-2.40	14.25	58.40	39.53	35.23	31.58	/	24.49	46.56	19.81
a2	78.62	56.66	63.52	45.84	/	51.39	-2.50	27.88	61.57	54.07	42.75	47.98	/	35.44	74.43	26.30
a3	243.42	68.70	216.10	59.88	/	85.16	-10.61	74.60	237.74	63.53	221.82	59.07	/	119.94	226.67	83.50

Table S12. *A*-tensors for Co in **[(Mebbpya)Co]^x** calculated with the two different functionals BP86 and B3LYP.

	BP86								B3LYP							
	[(Mebbpya)Co] + 2OTf		[(Mebbpya)Co]$^{2+}$		[(Mebbpya)Co]$^{+}$		[(Mebbpya)Co]^{0}		[(Mebbpya)Co]+2OTf		[(Mebbpya)Co]$^{2+}$		[(Mebbpya)Co]$^{+}$		[(Mebbpya)Co]^{0}	
total	S=1/2	S=3/2	S=1/2	S=3/2	S=0	S=1	S=1/2	S=3/2	S=1/2	S=3/2	S=1/2	S=3/2	S=0	S=1	S=1/2	S=3/2
a1	75.59	37.55	27.30	32.08	/	26.49	-0.60	11.12	57.90	40.21	30.98	36.48	/	17.41	23.30	12.73
a2	82.82	54.72	64.13	35.18	/	52.44	-1.95	27.21	65.29	51.32	58.34	38.72	/	33.37	63.37	22.35
a3	244.70	69.63	210.83	63.59	/	75.52	-11.01	72.43	238.92	65.18	223.03	62.10	/	114.27	208.32	78.27

Table S13. *A*-tensors for all N in **[(Hbbpya)Co]^0** calculated with the two different functionals BP86 and B3LYP. N1-N4 being to the Co coordinating ones, N5 being the bridging N, and N6-N7 being the N of ACN coordinating to the Co center.

	BP86													
	S=1/2							S=3/2						
total	N1	N2	N3	N4	N5	N6	N7	N1	N2	N3	N4	N5	N6	N7
a1	-1.95	-0.84	-0.84	-1.94	-0.19	-0.09	-0.09	-0.25	0.33	0.20	0.28	0.04	19.85	0.10
a2	-2.10	-1.01	-1.01	-2.09	-0.32	-0.13	-0.13	-0.63	0.86	0.61	0.70	0.14	19.89	0.10
a3	8.70	4.14	4.14	8.69	0.60	0.30	0.26	6.95	6.12	5.90	7.25	0.22	22.01	0.64
total	B3LYP													
a1	-3.15	-4.33	-5.00	-2.04	0.11	39.16	0.24	-1.68	-1.88	-1.81	-1.10	-0.13	12.77	0.49
a2	-3.66	-4.78	-5.73	-3.14	0.20	39.26	0.29	-1.91	-2.08	-1.98	-1.42	0.16	12.82	0.52
a3	-27.17	-20.92	13.82	22.75	0.78	43.47	1.01	6.89	4.58	4.53	7.33	0.31	14.24	1.12

Table S14. *A*-tensors for all N in **[(Mebbpya)Co]^0** calculated with the two different functionals BP86 and B3LYP. N1-N4 being to the Co coordinating ones, N5 being the bridging N, and N6-N7 being the N of ACN coordinating to the Co center.

	BP86													
	S=1/2							S=3/2						
total	N1	N2	N3	N4	N5	N6	N7	N1	N2	N3	N4	N5	N6	N7
a1	-1.97	-0.88	-1.38	-2.11	0.12	-0.11	-0.04	-0.84	0.43	0.40	0.41	0.04	19.60	0.04
a2	-2.09	-1.02	-1.52	-2.28	-0.16	-0.11	-0.07	-1.24	1.11	0.76	0.83	0.12	19.64	0.05
a3	8.35	5.45	5.41	8.28	-0.27	0.19	0.15	6.47	6.56	6.36	7.37	0.30	21.74	0.49
total	B3LYP													
a1	-3.09	-4.97	-4.97	-0.47	0.07	41.07	0.00	-1.57	-1.61	-1.66	-0.66	0.13	14.25	0.31
a2	-3.62	-5.28	-5.62	-1.82	0.43	41.14	-0.01	-1.84	-1.84	-1.82	-1.03	0.29	14.29	0.34
a3	-25.91	-23.70	16.91	23.49	0.95	45.39	0.54	7.03	5.68	5.65	7.66	-0.31	15.81	0.77

Catalytic cycle - The thermodynamics of a direct dehydrogenation of two [(Hbbpya)Co]^0 to two [(bppya)-Co]^0 plus H_2 as well as some initial CO_2 association to [1/(Mebbpya)Co] have been conducted with the def2-TZVP(-f) basis set, AutoAux for generation of an auxiliary basis set for integral acceleration by means of the RI or RIJCOSX approximation, d3bj for dispersion correction and C-PCM for acetonitrile.[21-33] Furthermore, the keywords TightOpt and TightSCF have been invoked. The used functionals for each calculation are indicated in the tables below, listing resulting energies as well. If a transition state is given the reactants and products structure and energies result from an IRC calculation of the given transition state. Only exception to this is in HER the H_2 formation with the B3LYP functional, where the IRC product side happened to converge to minima coordinating a phenol to the metal center, or to form a η^2-H_2 coordinating complex. Since these might be reasonable minima but aren't the dissociated structure, the dissociation was enforced by hand (not through a constraint, but through a different starting structure). The initial and other minima found on the way to the desired product are listed as well.

Table S15. Energies of the dehydrogenation of **[(Hbbpya)Co]^0** reactant and product and reaction Gibbs free energy with four functionals.

functional	Reac [Hartree]	Prod [Hartree]	d_RG [kcal/mol]
BP86	-5122.1873	-5122.2006	-8.35
TPSSH	-5121.7187	-5121.7565	-23.73
B3LYP	-5120.0216	-5120.0580	-22.85
PBE0	-5118.2184	-5118.2279	-5.96

Table S16. Energies of the CO_2-associations reactant, transition state and product, and activation and gibbs free energies.

	Reac [Hartree]	TS [Hartree]	Prod [Hartree]	E_A [kcal/mol]	d_RG [kcal/mol]
TPSSh [(Hbbpya)Co]^0	-2616.7713	-2616.7693	-2616.7775	1.22	-3.90
PBE0 [(Hbbpya)Co]^0	-2614.9868	-2614.9796	-2614.9851	4.52	1.06
PBE0 [(Mebbpya)Co]^0	-2654.2181	-2654.2127	-2654.2199	3.36	-1.15

In the following the energies of the $CO_2H_{0/1}$-protonation reactions, the hydrogen evolution reaction, CO-dissociation, further CO_2-associations, Co-protonation, NR-protonation and the phenol-cluster assisted CO_2-association are given. All these calculations utilized the same settings as mentioned before but the def2-TZVP[21] basis set for all atoms but the def2-TZVPP[21] basis set for cobalt.

Table S17. Energies of previously mentioned reactions. a be aware that these TS do not correspond to the given product in the classical sense, since the IRC didn't lead to the latter. b The IRC of this TS led to the initially H^+-donating phenol ending up as the phenolate. There is a small barrier to the protonation of this first phenol so that the phenolate will be stabilized by three other phenols, which is the corresponding minimum for **$[(Hbbpya)Co\text{-}CO_2H]^+$**. Overcoming the first minimum leads to the desired product state, which also results in the same dRG. c This energy difference is the only one that corresponds to the sum of each product in their own calculations Gibbs free energy.

	Energies [Hartree]			Energies [kcal/mol]	
	Reactant	TS (if available)	Product	E_A (if available)	d_RG
BP86	CO_2 Association far away thermodynamic only				
$[(Hbbpya)Co]^0$	-2616.9987		-2617.0138		-9.46
$[(Mebbpya)Co]^0$	-2656.2842		-2656.3003		-10.08
BP86	CO_2 Association IRC				
$[(Hbbpya)Co]^0$	-2617.0053	-2617.0035	-2617.0138	1.14	-5.33
$[(bbpya)Co]^0$	CO_2 Association $[(bppya)Co]^{0/-}$				
BP86	-2616.4225		-2616.4314		-5.54
B3LYP	-2615.3479		-2615.3516		-2.34
PBE0	-2614.4091		-2614.4123		-1.99
$[(bbpya)Co]^-$					
BP86	-2616.5314		-2616.5479		-10.33
B3LYP	-2615.4417		-2615.4539		-7.65
PBE0	-2614.5113		-2614.5167		-3.42
BP86	PhOH + M		PhO^- + MH^+	Co - Protonation thermodynamics	
$[(Hbbpya)Co]^0$	-3658.4738		-3658.4884		-9.18
$[(Mebbpya)Co]^0$	-3697.7634		-3697.7673		-2.46

B3LYP	PhOH +M		PhO$^-$ + MH$^+$	Co - Protonation thermodynamics	
[(Hbbpya)Co]^0	-3696.0162		-3696.0196		-2.12
[(Mebbpya)Co]^0	-3656.7560		-3656.7698		-8.67
BP86	NRH$^+$ + M		NR + MH$^+$	Co - Protonation thermodynamics	
[(Hbbpya)Co]^0	-3658.4515		-3658.4884		-23.13
[(Mebbpya)Co]^0	-3697.7424		-3697.7673		-15.61
B3LYP	NRH$^+$ + M		NR + MH$^+$	Co - Protonation thermodynamics	
[(Hbbpya)Co]^0	-3656.7231		-3656.7689		-28.73
[(Mebbpya)Co]^0	-3695.9875		-3696.0197		-20.18
[(Hbbpya)Co]^0	CO_2 Association - PhOH-cluster supported thermodynamics only				
BP86	-3847.1570		-3847.1814		-15.36
B3LYP	-3845.3366		-3845.3629		-16.47
BP86	CO_2 Association - PhOH-cluster supported IRC def2-SVP basis set				
[(Hbbpya)Co]^0	-3844.3092	-3844.3010	-3844.3254	5.17	-10.11
BP86	HER - H_2 formation - dissociated product				
[(Hbbpya)Co]^0	-3658.9294		-3658.9411		-7.34
[(Mebbpya)Co]^0	-3698.2099		-3698.2184		-5.35
B3LYP	HER - H_2 formation - dissociated product				
[(Hbbpya)Co]^0	-3657.2204	-3657.2222	-3657.2443	-1.17[a]	-15.01
[(Mebbpya)Co]^0	-3696.4718	-3696.4715	-3696.4967	0.18[a]	-15.63
BP86	HER - H_2 formation - H_2-adduct				
[(Hbbpya)Co]^0	-3658.9294		-3658.9336		-2.67
[(Mebbpya)Co]^0					
B3LYP	HER - H_2 formation - H_2-adduct				
[(Hbbpya)Co]^0	-3657.2204		-3657.2384		-11.29
[(Mebbpya)Co]^0					
BP86	HER - H_2 formation - PhOH-adduct				

[(Hbbpya)Co]^0	-3658.9294		-3658.9480		-11.68
[(Mebbpya)Co]^0	-3698.2099		-3698.2306		-12.98
B3LYP	HER - H_2 formation - PhOH-adduct				
[(Hbbpya)Co]^0	-3657.2204		-3657.2532		-20.61
[(Mebbpya)Co]^0	-3696.4718		-3696.5060		-21.41
B3LYP	1st Protonation				
[(Hbbpya)Co]^0 + 4 PhOH	barrierless				
[(Mebbpya)Co]^0 + 4 PhOH	barrierless				
[(Hbbpya)Co]^0 + 3 PhOH	-3538.0027	-3538.0034	-3538.0057	-0.45	-1.90
[(Hbbpya)Co]^0 + 1 PhOH	-2923.2866	-2923.2800	-2923.2783	4.14	5.19
BP86	1st Protonation				
[(Hbbpya)Co]^0 + 4 PhOH	-3847.1821	-3847.1818	-3847.1807	0.18	0.82
B3LYP	2nd Protonation				
[(Hbbpya)Co-CO_2H]$^+$ + 4 PhOH	-3845.8108	-3845.8023	-3845.8221	5.36	-7.06
[(Mebbpya)Co-CO_2H]$^+$ + 4 PhOH	-3885.0675	-3885.0587	-3885.0733	5.52	-3.61
[(Mebbpya)Co-CO_2H]$^+$ + 4 PhOH[b]	-3885.0675		-3885.0788[b]		-7.07[b]
[(Hbbpya)Co-CO_2H]$^+$ + 3 PhOH	-3538.4554	-3538.4476	-3538.4585	4.94	-1.94
[(Hbbpya)Co-CO_2H]$^+$ + 1 PhOH	-2923.7386	-2923.7238	-2923.7349	9.24	2.31
B3LYP	CO Dissociation - Separated calculations[c]				
[(Hbbpya)Co-CO]2+	-2540.4086		-2540.4089		-0.19

Table S18. Reaction energy diagrams total and relative (to the reactants) energies. Calculated with BP86 functional.

	[(Hbbpya)Co]		[(Mebbpya)Co]	
	E [Hartree]	$\Delta_R G$ [kcal mol^{-1}]	E [Hartree]	$\Delta_R G$ [kcal mol^{-1}]
$[x\text{-}Co]^0 + HA + CO_2$	-3847.6127	0.00	-3886.9007	0.00
$[x\text{-}Co\text{-}CO_2]^0 + HA$	-3847.6137	-0.57	-3886.9008	-0.08
$[x\text{-}Co\text{-}CO_2]^0_{rear.} + HA$	-3847.6192	-4.06	-3886.9033	-1.64
TS1	-3847.6189	-3.88	-3886.9048	-2.54
$[x\text{-}Co\text{-}CO_2H]^+ + A^-$	-3847.6179	-3.24	-3886.9024	-1.06
$[x\text{-}Co\text{-}CO_2H]^+ + HA$	-3847.6131	-0.21	-3886.8960	2.97
TS2	-3847.6034	5.88	-3886.8880	7.96
$[x\text{-}Co\text{-}CO]^{2+} + A^- + H_2O$	-3847.6201	-4.62	-3886.8983	1.51
$[x\text{-}Co\text{-}H_2O]^+ + A^- + CO$	-3847.6108	1.24	-3886.8891	7.31
$[x\text{-}Co\text{-}H_2O]^+ + A^- + CO$	-3847.6212	-5.32	-3886.8990	1.05

Table S19. CO Dissociation thermodynamics.

B3LYP	CO Dissociaton					
	$[(Hbbpya)Co\text{-}CO]^{2+}$		$[(Hbbpya)Co\text{-}CO]^{+}$		$[(Hbbpya)Co\text{-}CO]^{0}$	
Spin state	S=1/2	S=3/2	S=0	S=1	S=1/2	S=3/2
Reactant	-2540.4086	-2540.3958	-2540.5425	-2540.5642	-2540.6681	-2540.6637
Product	-2540.3942	-2540.3845	-2540.5338	-2540.5292	-2540.6436	-2540.6308
	$[(Hbbpya)Co\text{-}CO]^{2+}$		$[(Hbbpya)Co\text{-}CO]^{+}$		$[(Hbbpya)Co\text{-}CO]^{0}$	
Spin state	S=1/2	S=3/2	S=0	S=1	S=1/2	S=3/2
Reactant	0.00	8.06	13.63	0.00	0.00	2.78
Product	9.05	15.15	19.09	21.99	15.36	23.44
	$[(Mebbpya)Co\text{-}CO]^{2+}$		$[(Mebbpya)Co\text{-}CO]^{+}$		$[(Mebbpya)Co\text{-}CO]^{0}$	
Spin state	S=1/2	S=3/2	S=0	S=1	S=1/2	S=3/2
Reactant	-2579.6685	-2579.6543	-2579.8047	-2579.8239	-2579.9300	-2579.9240
Product	-2579.6521	-2579.6388	-2579.7898	-2579.7823	-2579.8995	-2579.8882
	$[(Mebbpya)Co\text{-}CO]^{2+}$		$[(Mebbpya)Co\text{-}CO]^{+}$		$[(Mebbpya)Co\text{-}CO]^{0}$	
Spin state	S=1/2	S=3/2	S=0	S=1	S=1/2	S=3/2
Reactant	0.00	8.92	12.07	0.00	0.00	3.73
Product	10.30	18.64	21.40	26.10	19.16	26.22

General procedure for localization of TS.

Transition state guesses have been acquired by either scans or, due to the simplicity of most reaction steps in terms of change in geometry, guessed from an optimized structure. These guesses then have been evaluated by the calculation of a numerical Hessian with xtb[34[33]], and then optimized on a lower level of theory first (def2-SVP), then on the higher level of theory. If the transition state optimization with the xtb-Hessian as the guess-Hessian didn't converge or even diverged, the guess-Hessian has been calculated on DFT level. If this still wasn't successful, the level of theory was stepwise increased (svp-Hessian + tzvp-optTS, tzvp-Hessian + tzvp-optTS, svp-Hessian + tzvp-Hessian + recalculation of the Hessian throughout the calculation, and so on). In the case of $[(Mebbpya)Co]^{0}$ + 4 phenol, even though a promising imaginary mode has been localized, even the greatest effort we could muster didn't lead to a converged TS structure. In this case comparing the reactivity of $[(Hbbpya)Co]^{0}$ and $[(Mebbpya)Co]^{0}$ in other reaction steps was helpful, since there, little to no difference between the two specimens could be seen. Assuming that this holds true for the first protonation, too, and with the difficulty to converge

the TS and the fact that diverging transition state optimization converged to the protonation product with imaginary modes somewhere else in the molecule, we assume this transition to be barrier less, too.

9 References

[1] S. Stoll, A. Schweiger, *J Magn Reson* **2006**, *178*, 42-55.

[2] G. Sheldrick, *Acta Crystallographica Section C* **2015**, *71*, 3-8.

[3] O. V. Dolomanov, L. J. Bourhis, R. J. Gildea, J. A. K. Howard, H. Puschmann, *Journal of Applied Crystallography* **2009**, *42*, 339-341.

[4] S. Roy, B. Sharma, J. Pecaut, P. Simon, M. Fontecave, P. D. Tran, E. Derat, V. Artero, *J Am Chem Soc* **2017**, *139*, 3685-3696.

[5] L. Homberg, A. Roller, K. C. Hultzsch, *Org Lett* **2019**, *21*, 3142-3147.

[6] D. den Boer, A. I. Konovalov, M. A. Siegler, D. G. H. Hetterscheid, *Inorg Chem* **2023**, *62*, 5303-5314.

[7] W. Verbeet, Y. Husiev, S. Bonnet, *European Journal of Organic Chemistry* **2024**, *27*.

[8] S. Zheng, N. R. Reintjens, M. A. Siegler, O. Roubeau, E. Bouwman, A. Rudavskyi, R. W. Havenith, S. Bonnet, *Chemistry* **2016**, *22*, 331-339.

[9] Deposition numbers 2384008, 2384005, 2384007, 2384011, 2384004, 2384010, and 2384006 contain the supplementary crystallographic data for this paper. These data are provided free of charge by the joint Cambridge Crystallographic Data Centre and Fachinformationszentrum Karlsruhe Access Structures service.

[10] F. Neese, *Wiley Interdisciplinary Reviews: Computational Molecular Science* **2012**, *2*, 73-78.

[11] F. Neese, *WIREs Computational Molecular Science* **2012**, *2*, 73-78.

[12] F. Neese, F. Wennmohs, U. Becker, C. Riplinger, *The Journal of Chemical Physics* **2020**, *152*, 224108.

[13] F. Neese, *WIREs Computational Molecular Science* **2022**, *12*, e1606.

[14] E. F. Valeev, *For the current version, see https://github. com/evaleev/libint/tree/v1* **2020**.

[15] S. Lehtola, C. Steigemann, M. J. T. Oliveira, M. A. L. Marques, *SoftwareX* **2018**, *7*, 1-5.

[16] A. D. Becke, *Physical review A* **1988**, *38*, 3098.

[17] J. P. Perdew, *Physical review B* **1986**, *33*, 8822.

[18] J. Tao, J. P. Perdew, V. N. Staroverov, G. E. Scuseria, *Physical review letters* **2003**, *91*, 146401.

[19] C. Lee, W. Yang, R. G. Parr, *Physical review B* **1988**, *37*, 785.

[20] C. Adamo, V. Barone, *The Journal of chemical physics* **1999**, *110*, 6158-6170.

[21] F. Weigend, R. Ahlrichs, *Physical Chemistry Chemical Physics* **2005**, *7*, 3297-3305.

[22] G. L. Stoychev, A. A. Auer, F. Neese, *Journal of chemical theory and computation* **2017**, *13*, 554-562.

[23] J. L. Whitten, *The Journal of Chemical Physics* **1973**, *58*, 4496-4501.

[24] E. J. Baerends, P. Ros, *Chemical Physics* **1975**, *8*, 412-418.

[25] B. I. Dunlap, J. W. D. Connolly, J. R. Sabin, *The Journal of Chemical Physics* **1979**, *71*, 3396-3402.

[26] C. Van Alsenoy, *Journal of computational chemistry* **1988**, *9*, 620-626.

[27] R. A. Kendall, H. A. Früchtl, *Theoretical Chemistry Accounts* **1997**, *97*, 158-163.

[28] K. Eichkorn, O. Treutler, H. Öhm, M. Häser, R. Ahlrichs, *Chemical physics letters* **1995**, *240*, 283-290.

[29] K. Eichkorn, F. Weigend, O. Treutler, R. Ahlrichs, *Theoretical Chemistry Accounts* **1997**, *97*, 119-124.

[30] F. Neese, F. Wennmohs, A. Hansen, U. Becker, *Chemical Physics* **2009**, *356*, 98-109.

[31] S. Grimme, J. Antony, S. Ehrlich, H. Krieg, *The Journal of chemical physics* **2010**, *132*.

[32] V. Barone, M. Cossi, *The Journal of Physical Chemistry A* **1998**, *102*, 1995-2001.

[33] C. Bannwarth, S. Ehlert, S. Grimme, *Journal of chemical theory and computation* **2019**, *15*, 1652-1671.
